# MicroRNA expression profiles in abdominal aortic aneurysms: A systematic review of potential diagnostic and prognostic biomarkers

**DOI:** 10.1016/j.ijcrp.2026.200651

**Published:** 2026-05-07

**Authors:** Pooya Eini, Peyman Eini, Homa serpoush, Mohammad Rezayee, Jason Tremblay

**Affiliations:** a Cardiovascular Research Center, Rajaie Cardiovascular Institute, Tehran, Iran; b Infectious Disease Research Center, Hamadan University of Medical Sciences, Hamadan, Iran; c Hamadan University of Medical Sciences, Hamadan, Iran; d College of Human Medicine, Michigan State University, East Lansing, MI, USA

**Keywords:** Abdominal aortic aneurysm (AAA), microRNA (miRNA), Vascular remodeling, Circulating miRNAs, Tissue miRNAs

## Abstract

**Background:**

MicroRNAs (miRNAs) are critical regulators of vascular biology and have been implicated in the pathogenesis of abdominal aortic aneurysm (AAA). However, the diversity of study designs and heterogeneous findings has limited their clinical translation. This study aimed to systematically review available evidence on miRNA expression in AAA, both in aortic tissue and circulating blood, and to explore their potential regulatory pathways.

**Methods:**

We conducted a comprehensive literature search across five databases —PubMed, Scopus, Embase, Web of Science (WoS), and EBSCO — up to June 2025, following the PRISMA guidelines. Eligible studies included those reporting differential miRNA expression in human AAA samples, whether tissue or blood, with validated results. Data on expression direction, sample type, pathways, and target genes were extracted and synthesized.

**Results:**

A total of 39 studies were included reported diagnostic performance varied substantially across studies. Circulating miRNAs in AAA patients exhibited distinct dysregulation patterns, reflecting disease-associated vascular and inflammatory processes. Key Up-regulated miRNAs included miR-21, miR-146a, miR-155, miR-1281, and miR-34a, while prominent Down-regulated candidates comprised miR-15a, miR-29, miR-150-5p, miR-27a-3p, and let-7 family members. These alterations were observed in plasma, serum, and specific cell types, suggesting possible utility as non-invasive biomarker candidates for AAA detection and disease monitoring, although external validation remains limited.

**Conclusion:**

Our systematic review highlights a panel of consistently dysregulated miRNAs in AAA, with roles in inflammation, extracellular matrix remodeling, and vascular cell regulation, and they may represent promising candidates for future screening and diagnostic evaluation, pending standardization and prospective validation.

## Highlights

• This systematic review synthesizes evidence from 39 studies to map consistent microRNA expression changes in abdominal aortic aneurysms across tissue and circulating samples.
• Distinct panels of up-regulated and down-regulated miRNAs were identified, linking AAA to inflammation, extracellular matrix remodeling, and vascular cell dysfunction.
• These consistently dysregulated miRNAs show strong potential as non-invasive diagnostic and prognostic biomarkers for AAA detection and disease monitoring.

## 1 Introduction

Aortic aneurysms, including abdominal aortic aneurysm (AAA) and thoracic aortic aneurysm (TAA), represent life-threatening conditions characterized by localized dilation of the aortic wall, often leading to rupture if untreated [1]. Aortic aneurysms are more prevalent in the abdominal aorta, with a higher incidence in older populations [2] and associations with risk factors such as smoking, hypertension, and atherosclerosis [3]. TAA, though less common, is often linked to genetic predispositions and connective tissue disorders, but can also occur in non-syndromic forms [4]. Both conditions share pathological features, including inflammation, extracellular matrix (ECM) degradation, and vascular smooth muscle cell (VSMCs) dysfunction [5,6]. Despite advances in imaging and surgical interventions, early detection and risk stratification remain challenging due to the asymptomatic nature of aneurysms and the lack of reliable non-invasive biomarkers.

MicroRNAs (miRNAs) are small, non-coding RNAs that regulate gene expression post-transcriptionally by targeting messenger RNAs (mRNAs) [7], influencing pathways critical to vascular biology, such as inflammation, angiogenesis, apoptosis, and ECM remodeling [8]. Recent evidence suggests that miRNAs are differentially expressed in aneurysmal tissues and plasma, offering potential as diagnostic and prognostic biomarkers [2,3]. Studies have identified miRNA signatures in AAA [6], with some miRNAs (e.g., miR-21, miR-155, miR-205) showing consistent dysregulation across cohorts, while others exhibit variability, possibly due to differences in sample type (tissue vs. plasma), aneurysm location, or study methodology [4]. These miRNAs are implicated in key pathogenic pathways, including transforming growth factor-beta (TGF-β) signaling, vascular endothelial growth factor (VEGF) pathways, and matrix metalloproteinase (MMP) regulation, which are central to aneurysm development.

Although tissue miRNAs provide direct insights into local pathogenic mechanisms within the aneurysmal aortic wall, circulating miRNAs (in plasma, serum, or whole blood) offer practical advantages for non-invasive clinical translation. Diagnostic biomarkers aim to improve early detection of AAA in asymptomatic individuals or high-risk populations (e.g., elderly smokers or those with family history), where current screening relies solely on imaging that is resource-intensive and not universally implemented. Prognostic biomarkers, in contrast, seek to stratify patients according to risk of rapid growth, rupture, or adverse outcomes post-intervention, potentially guiding surveillance intervals, timing of elective repair, or identification of those who may benefit from intensified medical management.

Previous reviews have largely focused on individual miRNAs, experimental mechanisms, or mixed aneurysm phenotypes. This systematic review aimed to consolidate evidence on miRNA expression profiles in abdominal aortic aneurysms, quantify their differential expression between aneurysmal and non-aneurysmal samples, and evaluate their potential as biomarkers for diagnosis and prognosis. The present study specifically synthesizes contemporary human evidence in AAA, separately evaluating tissue and circulating miRNA profiles. By addressing variability in study design, sample types, and miRNA expression, this study aimed to provide insights into the molecular mechanisms underlying aortic aneurysms and to inform future research and clinical applications.

## 2 Methods

### 2.1 Study design and registration

This systematic review was conducted in accordance with the Preferred Reporting Items for Systematic Reviews and Meta-Analyses (PRISMA) guidelines [9]. The protocol has been registered with PROSPERO (CRD420251102691) to ensure transparency and reproducibility.

### 2.2 Eligibility criteria

Studies were included if they met the following criteria:

1. **Study Type**: Observational human studies (case-control, cohort, or cross-sectional) reporting differential miRNA expression in AAA.
2. **Participants**: Adults (≥18 years) with non-syndromic degenerative AAA diagnosed by imaging (ultrasound, CT, or angiography), typically defined by infrarenal aortic diameter ≥30 mm or ≥50% enlargement relative to normal diameter.
3. **Sample Types**: Studies evaluating miRNA expression in aortic wall tissue, circulating blood (plasma, serum, whole blood, or cellular fractions such as PBMCs or RBCs), or exosomes derived therefrom. Studies reporting both tissue and circulating data were included and analyzed separately to avoid conflation of local versus systemic signals. When a single study presented mixed sample types, results were stratified by biospecimen (tissue vs. blood) during data extraction and synthesis.
4. **Outcomes**: Quantitative data on miRNA expression direction (up- or down-regulation), fold change, p-values, and, where available, diagnostic performance (AUC, sensitivity, specificity) or prognostic associations (e.g., correlation with diameter, growth rate, FDG uptake, or post-EVAR changes).
5. **Methodology**: Use of validated quantification methods (qRT-PCR, microarray, or next-generation sequencing) with reported normalization strategies.

**Exclusion criteria** included: animal or in vitro mechanistic studies without accompanying human expression data (to maintain clinical relevance and focus on translational potential); studies on syndromic, inflammatory, mycotic, or thoracic aortic aneurysms unless non-syndromic AAA cases were analyzed and reported separately; case reports, reviews, conference abstracts, and non-English publications.

**Rationale for exclusions**: Non-human and purely mechanistic studies, while valuable for pathway elucidation, were excluded because the primary objective of this review was to evaluate the clinical biomarker potential of miRNAs in humans. Inclusion of such studies would have introduced substantial heterogeneity in models and endpoints without directly informing diagnostic or prognostic utility in patients. Mixed aneurysm phenotypes were excluded to ensure specificity to abdominal aortic disease, given known differences in pathophysiology between thoracic and abdominal aneurysms.

### 2.3 Search strategy

A comprehensive literature search conducted across the PubMed, Embase, Web of Science, Ebsco, and Scopus databases to identify relevant studies. The search strategy combined Medical Subject Headings (MeSH) and Emtree terms, including:

● **Disease terms**: "aortic aneurysm," "abdominal aortic aneurysm, " "AAA,".
● **miRNA terms**: "microRNA," "miRNA," "miR," "non-coding RNA."
● **Outcome terms**: "expression," "biomarker," "diagnosis," "prognosis," "differential expression."

Full database-specific search strategies, including Boolean operators and controlled vocabulary terms, are provided in Supplementary Table 1. Only English-language peer-reviewed studies were included.

### 2.4 Study selection

Two independent reviewers screened titles and abstracts using predefined criteria, followed by full-text review. Discrepancies were resolved by consensus or consultation with a third reviewer. A PRISMA flow diagram documented the selection process, including reasons for exclusion.

### 2.5 Data extraction

Two reviewers independently extracted data using a standardized form, and disagreements were resolved through discussion with adjudication by a third reviewer when required.

● **Study Characteristics**: Author, year
● **Participant Details**: Age, sex, diagnostic criteria, control group definition, past medical history.
● **miRNA Data**: miRNA name, sample type (tissue, plasma, red blood cells), expression direction (Up-regulated/Down-regulated), fold change, p-value if reported.
● **Pathway and Clinical Relevance**: Associated molecular pathways (inflammation, ECM remodeling), diagnostic metrics (AUC, sensitivity, specificity), prognostic correlations (with aneurysm size, complications).

### 2.6 Quality assessment

The Newcastle-Ottawa Scale (NOS) was used to assess the quality of included studies, evaluating selection, comparability, and outcome domains. Studies scoring ≥7 were considered high quality [10]. Two reviewers independently assessed quality, with disagreements resolved by discussion. Study quality was additionally considered during interpretation, with greater emphasis placed on findings replicated across moderate-to high-quality studies.

## 3 Results

### 3.1 Study selection

**Fig. 1:**
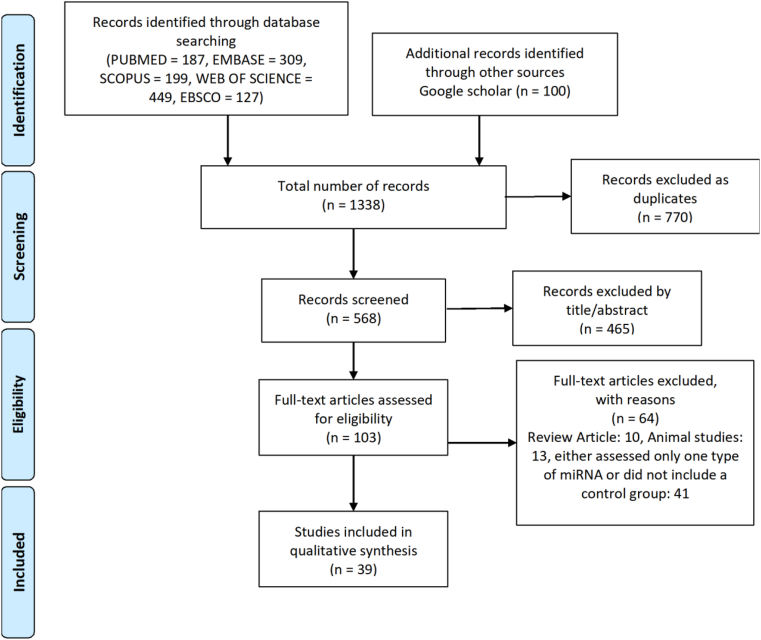
PRISMA flow diagram for study selection.

Records were identified through multiple databases, including MEDLINE (n = 187), EMBASE (n = 309), SCOPUS (n = 199), WEB OF SCIENCE (n = 449), and EBSCO (n = 127). Additional records were retrieved from Google Scholar (n = 100) to capture grey literature, bringing the total to 1338. Seven hundred seventy records were excluded as duplicates, leaving 568 records for title/abstract evaluation. After screening titles and abstracts, 102 articles were deemed eligible for full-text review. Ultimately, 39 studies were included in the analysis (Fig. 1). Data for 263 microRNAs assessed across 41 studies—either evaluated in only a single study or lacking a control group—are provided in Supplementary Table 2.

### 3.2 Baseline characteristics of included studies

A total of 39 studies evaluating miRNA expression in patients with AAA were included in this systematic review. The baseline characteristics are summarized in Table 1. Case definitions predominantly relied on imaging modalities such as computed tomography (CT), ultrasound, or angiography to confirm AAA, with diameter thresholds being the most common criterion. The majority of studies (n = 24) defined AAA as an aortic diameter ≥30 mm,> 5 cm, or> 50% of the normal diameter, often specifying an infrarenal location. Surgical eligibility or intervention (endovascular repair or open resection) was incorporated in 17 studies. At the same time, additional criteria, such as aneurysm growth rate (>10 mm/6 months) or Fluorodeoxyglucose (FDG) uptake (indicating inflammation), were used in select cases (n = 3). One study focused on AAA with post-diagnosis shrinkage (≥5 mm over 6 months), highlighting a subset with potential regression. Non-aneurysmal aortic tissue from organ donors or autopsies was the most frequent comparator (n = 14), providing histologically normal references. Healthy volunteers without AAA, confirmed by imaging or clinical examination, served as controls in 10 studies, sometimes matched for age, gender, or risk factors. Adjacent non-dilated aortic segments (AAA neck <30 mm or <45 mm) were used in 5 studies, which may better account for patient-specific factors but could include subclinical pathology. Unique controls included patients with peripheral arterial disease (PAD) and chronic venous disease, as well as preoperative baselines from the same AAA patients (n = 4), allowing intra-individual comparisons. Sample tissues for miRNA expression analysis were primarily aortic tissue (n = 22), often infrarenal or full-thickness samples, enabling direct assessment of local dysregulation in extracellular matrix remodeling and inflammation. Circulating biomarkers were explored in plasma or serum (n = 17), whole blood or peripheral venous blood (n = 5), and specialized cell types such as peripheral blood mononuclear cells (PBMCs), red blood cells, cultured smooth muscle cells (SMCs), human aortic SMCs (HASMCs), vascular SMCs (VSMCs), or laser-capture microdissected (LCM) macrophages (n = 7). Exosomes from tissue and plasma were analyzed in one study.

**Table 1:** Study characteristics.

Study	Case Definition	Control Definition	Past medical history of AAA patients	Sample Tissue
Maegdefessel_2012 [11]	AAA diagnosed by imaging or surgery	Non-aneurysmal aorta from organ donors	Not specified	Infrarenal aortic tissue
Maegdefessel_2013 [12]	Patients undergoing surgical repair for AAA (diameter 57–68 mm)	Organ donors without AAA (aortic tissue from heart or kidney explant)	Not specified	Infrarenal aortic tissue
Maegdefessel_2014 [13]	Abdominal aortic aneurysm tissue from patients undergoing surgery for AAA	Non-aneurysmal aortic tissue from organ donors or patients undergoing surgery for other vascular conditions	Not specified	Aortic tissue, plasma
Biros_2014 [14]	AAA diameter ≥50 mm by imaging	Non-dilated AAA neck, diameter <30 mm	Arterial Hypertension 5 (50%), Diabetes Mellitus 2 (20%), Dyslipidemia 6 (60%), CHD 3 (30%)	Aortic tissue, plasma
Spear_2015 [15]	AAA diameter >50 mm or growth >10 mm/6 months by ultrasound	Non-aneurysmal aorta from organ donors	Not specified	Aortic tissue, plasma
Stather_2015 [16]	AAA diameter >54 mm or 30-54 mm	Healthy, no AAA by ultrasound	Not specified	Whole blood
Zhang_2015 [17]	AAA diameter >50% normal by CT	Healthy, no AAA by ultrasound	Arterial Hypertension: 46 (76.7%), Dyslipidemia: 32 (53.3%), Diabetes Mellitus: 11 (18.3%), Coronary Artery Disease: 6 (10%), COPD: 5 (8.3%)	Plasma
Busch_2016 [18]	AAA diagnosed by imaging or surgery	Atherosclerotic non-aneurysmal aorta	Not specified	Aortic tissue
Wanhainen_2016 [19]	AAA ≥30 mm in diameter, with at least 6 months of follow-up, and ≥5 mm shrinkage during follow-up	Normal abdominal aorta at screening	Arterial Hypertension 62% (54–69%), Coronary Artery Disease 40% (33–48%), Cerebrovascular Disease 17% (12–24%), Diabetes Mellitus 12% (8–18%), Renal Insufficiency 6% (3–11%)	Plasma
Ni_2016 [20]	AAA diameter >50% normal by Doppler Ultrasound/CT/angiography	No AAA or severe chronic diseases	Diabetes Mellitus: 18(13%) Arterial Hypertension: 34 (26%) Coronary Artery Disease: 12 (9%) Chronic Renal Dysfunction: 4 (3%)	Red blood cells/plasma
Venkatesh_2017 [21]	AAA diameter >5.5 cm by imaging	Non-aneurysmal aorta <45 mm	Not specified	Aortic tissue
Gao_2017 [22]	Patients with AAA undergoing surgery	Aorta samples from autopsies without AAA or collagen disease	Not specified	Aortic tissue, plasma
Liang_2017 [23]	AAA diagnosed by imaging or surgery	Non-aneurysmal aorta	Not specified	Aortic tissue
Courtois_2017 [24]	AAA with positive uptake (A+)	AAA without FDG uptake (A0)	Arterial Hypertension (62%), COPD (45%), Diabetes Mellitus (8%), Hyperlipidemia (65%)	Aortic tissue, plasma
Ma_2018 [25]	AAA diagnosed by imaging or surgery	Non-aneurysmal aorta	Not specified	Blood
Riches_2018 [26]	AAA diagnosed by imaging or surgery	Non-aneurysmal saphenous vein or internal mammary artery	Not specified	SMCs
Tenorio_2018 [27]	Infrarenal AAA eligible for endovascular repair	Preoperative AAA patients (same patients, post-repair)	Arterial Hypertension: 20 (66.6%), Dyslipidemia: 8 (26.6%), Peripheral Arterial Disease: 5 (16.6%), Diabetes Mellitus: 5 (16.6%), COPD: 1 (3.3%), Coronary Artery Disease: 1 (3.3%), Stroke: 1 (3.3%)	Whole blood
Cerna_2019 [28]	Large AAA (>5 cm)	Non-aneurysmal aorta from cadaveric donors	Arterial hypertension: 12 (75%), Coronary Artery Disease: 9 (56%), Peripheral Arterial Disease: 6 (37%), Diabetes Mellitus: 3 (18%), Hyperlipidemia: 9 (54%), COPD: 5 (31%)	Aortic tissue
Spear_2019 [29]	AAA diagnosed by imaging or surgery	Non-aneurysmal aorta from organ donors	Hypercholesterolemia: 14 (58%), Arterial Hypertension: 15 (63%), Diabetes mellitus: 4 (17%)	Aortic tissue/M2 macrophages (LCM)
Araujo_2019 [30]	AAA diagnosed by imaging or surgery	Non-aneurysmal aorta from organ donors	Hypertension: 16 (88.9%), Dyslipidemia: 10 (55.5%), Diabetes: 3 (16.7%)	Aortic tissue
Zhao_2020 [31]	AAA diagnosed by imaging or surgery	Non-aneurysmal aortic tissue	Smoking: 15 (75%), Arterial Hypertension: 17 (85%), Peripheral artery Disease: 3 (15%), Dyslipidemia: 12 (60%), Diabetes Mellitus: 6 (30%)	Aortic tissue
Han_2020 [32]	AAA diagnosed by CTA (>5.5 cm)	Adjacent non-aneurysmal aortic tissue	Arterial Hypertension: 7 (33.3%)	Exosomes from aortic tissue and plasma
Plana_2020 [33]	AAA diagnosed by CT (>5.5 cm)	Organ donors (no AAA)	Arterial Hypertension: 17 (81.0%), Diabetes Mellitus: 10 (47.6%), Dyslipidemia: 13 (61.9%)	Aortic tissue, plasma
Torres-Do Rego_2020 [34]	AAA (aortic diameter ≥30 mm)	Normal aortic diameter (<25 mm)	Arterial Hypertension: 22 (100%), Dyslipidemia: 13 (59.1%), Coronary Artery Disease: 4 (18.2%), Cerebrovascular Disease: 1 (4.5%), Peripheral artery Disease: 4 (18.2%)	Plasma
Zhang_2020 [35]	Clinical diagnosis of AAA	Healthy individuals (medical examination)	Not specified	Serum
Zalewski_2020 [36]	AAA, confirmed by duplex ultrasonography and contrast-enhanced CT-Scan	Healthy, non-smoking volunteers	Arterial Hypertension 19 (67.9%), Coronary Artery Disease7 (25.0%), Diabetes Mellitus 6 (21.4%)	Peripheral blood mononuclear cells
Missae_2020 [37]	Degenerative infrarenal AAA eligible for endovascular repair	Pre-treatment AAA patients (baseline)	Arterial Hypertension: 20 (66.67%), Dyslipidemia: 8 (26.67%), Peripheral arterial disease: 5 (16.67%), Diabetes Mellitus: 5 (16.67%)	Peripheral venous blood
Lichołai_2021 [38]	AAA patients undergoing surgical stent graft implantation	Patients with peripheral arterial occlusive disease	Arterial Hypertension: 158 (77.07%), Coronary Artery Disease: 96 (46.83%), Cardiac failure: 21 (10.24%), Aortic stenosis: 7 (3.41%), Deep vein thrombosis: 6 (2.93%), Pulmonary embolism: 2 (0.98%), Stroke: 9 (4.39%), TIA: 2 (0.98%), Obstructive sleep apnea: 7 (3.41%), Gastric ulcer: 4 (1.95%), Analgetic therapy: 11 (5.37%), Nicotine: 130 (63.41%), Kidney failure: 3 (1.46%)	Serum
Zhou_2022 [39]	AAA patients undergoing infrarenal aorta replacement	Non-aneurysmatic aortic samples and healthy donor peripheral blood	Not specified	Aortic tissue and HASMCs
Wang_2022 [40]	AAA patients undergoing surgical resection	Adjacent normal aortic tissues	Not specified	Aortic tissue and HASMCs
Jing_2023 [41]	AAA patients diagnosed by CTA	Healthy individuals without AAA, frequency-matched by gender and age	Arterial Hypertension: 122 (61.0%), Diabetes: 28 (14.0%), Dyslipidemia: 130 (65.0%)	Serum
Thanigaimani_2023 [5]	AAA patients with infrarenal aortic diameter ≥30 mm	Healthy controls and PAD patients	Diabetes Mellitus: 19 (17.6%), Hypertension: 53 (49.1%), IHD: 43 (39.8%), TIA or Stroke: 15 (13.9%)	Serum
Li_2023 [42]	AAA patients diagnosed by CTA	Healthy controls without AAA	Smoking:133 (39.7%), Hypertension: 213 (63.6%), Diabetes Mellitus: 55 (16.4%), Dyslipidemia: 213 (63.6%)	Aortic tissue and blood leukocytes
Tian_2023 [43]	AAA patients	Normal aortic tissues from controls	Not specified	Aortic tissue
Cai_2024 [6]	AAA patients undergoing open repair	Normal aortas from organ donors	Not specified	Aortic tissue
Tasopoulou_2024 [4]	Patients with large AAAs (≥5.5 cm)	Volunteers with chronic venous disease (CEAP stages 0-2)	Arterial Hypertension: 50 (78.1%), Diabetes Mellitus: 16 (25.0%), Coronary Artery Disease: 16 (25.0%), Peripheral Artery Disease: 8 (12.5%), Chronic Kidney Disease: 8 (12.5%), Chronic Obstructive Pulmonary Disease: 6 (9.4%), Hyperlipidemia: 16 (25.0%)	Serum
Xiao_2024 [3]	Patients with AAA undergoing surgical repair and Ang II-induced AAA in NRF2ΔVSMC mice	Healthy human aortic tissue from donors and control mice	Not specified	Aortic tissue and VSMCs
Leite_2025 [2]	Patients with infrarenal AAA eligible for endovascular repair	Volunteers without AAA or risk factors	Not specified	Whole blood
Winski_2025 [44]	Patients diagnosed with AAA, defined as an aortic diameter ≥30 mm measured by ultrasound during the Swedish AAA screening program.	Age-matched male participants from the same screening program with an aortic diameter <30 mm and no diagnosis of AAA.	Arterial Hypertension: 109 (58%), Hyperlipidemia: 174 (93%), Coronary Artery Disease: 83 (44%), Diabetes Mellitus: 16 (9%)	Aortic tissue, plasma

Comorbidities among AAA patients were reported in 21 of the 39 studies, with hypertension being the most prevalent (ranging from 33.3% to 100% across cohorts), followed by dyslipidemia (26.6% to 93%), diabetes mellitus (3% to 47.6%), and coronary artery disease (3.3% to 56%). Other notable conditions included chronic obstructive pulmonary disease (COPD; 3.3% to 45%), peripheral arterial disease (PAD; 12.5% to 37%), and cerebrovascular events (stroke or transient ischemic attack; 0.98% to 17%). Smoking history was explicitly mentioned in a subset (63.4% to 75%), underscoring its role as a key risk factor. In studies without specified comorbidities (n = 18), potential selection biases or focus on molecular endpoints may limit generalizability.

Considerable heterogeneity was observed across the 39 included studies with respect to sample type (aortic tissue n = 22, circulating blood/exosomes n = 17, mixed or cell-specific n = 7), detection platforms (qRT-PCR predominant, with varying use of microarray or NGS), normalization strategies (e.g., endogenous controls such as U6 snRNA, miR-16, or global mean normalization), and control group selection (organ donor aorta, adjacent non-dilated segments, healthy volunteers, or patients with peripheral arterial disease). Patient cohorts also varied in AAA size thresholds (≥30 mm to ≥5.5 cm), surgical status, and comorbidity profiles (hypertension 33–100%, smoking history frequently >60%).

### 3.3 miRNAs in AAA aortic tissue and their functional implications

Differential expression analyses across studies have identified numerous dysregulated miRNAs in aortic tissues from patients with AAA. The expression of miR-1 showed inconsistent results: it remained unchanged compared with controls in one study [21], whereas others reported significant down-regulation (fold change −4.8)^42 33^. miR-1207-5p expression was unchanged, showing up-regulation in the qPCR array but lacking validation by qRT-PCR [30]. miR-125b-5p was consistently down-regulated, both in FDG-positive medial regions [24] and in AAA tissues overall [30]. miR-133a expression also varied, reported as unchanged in one study [21] but significantly down-regulated (fold change −4.4) in another [33]. miR-145 demonstrated context-dependent regulation: down-regulated in AAA tissues [21], yet up-regulated in AAA-derived SMCs [26] and in NRF2-positive VSMCs [3]. miR-146a was consistently up-regulated in AAA tissues [6,21,33], with one study reporting a 5.8-fold increase [33]. For miR-155, contrasting findings were observed: it was elevated in the aneurysm body compared with the neck (fold change 3.26) [14], yet decreased in other tissue analyses [21]. miR-15a-3p was down-regulated in adventitial tertiary lymphoid organs (fold change −3.32) [15], while miR-15a-5p and total miR-15a were Up-regulated in AAA cells and correlated positively with aneurysm diameter [22,44].

miR-204 and miR-204-5p were consistently down-regulated in AAA tissues and exosomes [21,24,32]. In contrast, miR-21 displayed a strong up-regulation in AAA tissues (6.7-fold in nonsmokers and 12.8-fold in smokers) [12], though its isoforms showed divergent results: miR-21-3p was down-regulated [18], miR-21-5p remained unchanged in one report [21], and was up-regulated (1.9-fold, in another [33].

miR-24-3p, miR-24, and miR-27a-3p were all down-regulated in large AAA tissues and exosomes [13,28,32]. miR-29 family members exhibited mixed patterns—miR-29a was unchanged, miR-29b was down-regulated, and miR-29c showed no significant difference [11]. Conversely, total miR-29 expression was increased in AAA tissue exosomes [32].

**Fig. 2:**
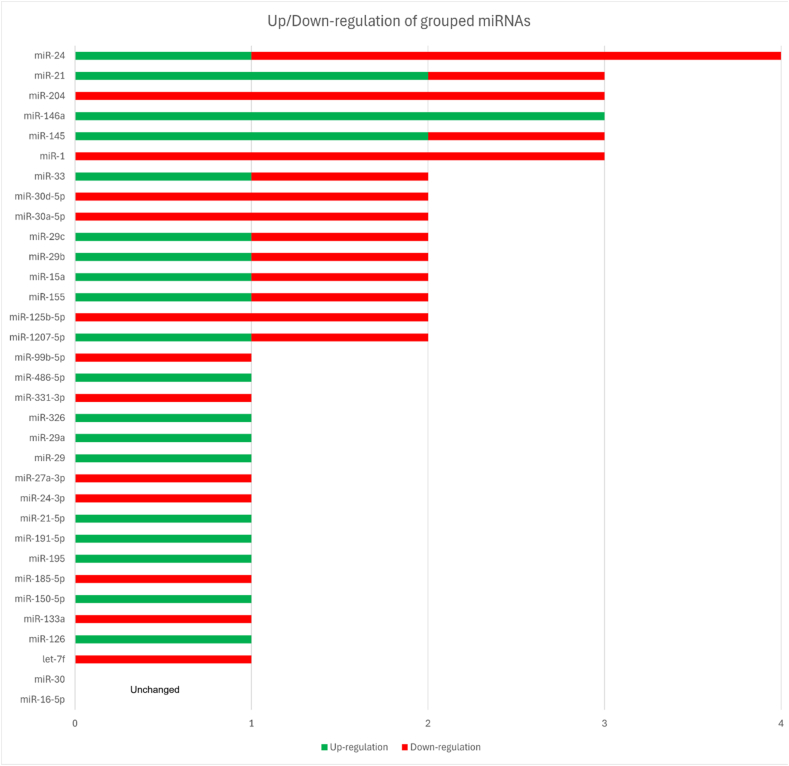
Stacked bar chart summarizing reported expression changes of grouped miRNAs in aortic tissue. Each bar represents one miRNA family (with variants such as -3p/-5p or a/b/c merged). Green segments indicate the number of studies reporting up-regulation, while red segments indicate the number of studies reporting down-regulation. The total bar length corresponds to the number of studies investigating each miRNA group, allowing quick comparison of consistency or conflicting findings across the literature. (For interpretation of the references to colour in this figure legend, the reader is referred to the Web version of this article.)

miR-30a-5p and miR-30d-5p were both down-regulated in adventitial and large AAA tissues [15,28]. miR-326 was up-regulated in both small and large AAAs [28]. miR-33 demonstrated spatial and compartmental differences, being down-regulated in aneurysmal body tissue [31] but up-regulated in AAA tissue exosomes [32]. (Table 2). These findings are visually represented in Fig. 2, which highlights the distribution of up- and down-regulation across miRNA families.

**Table 2:** miRNA Expression Profiles in Aortic Samples from AAA Patients.

miRNA	reference	Direction	Sample	Sample cells	Pathway	Keynote
let-7f	29	Down-regulation	Aortic tissue	M1/M2 macrophages	Not specified	Down-regulated in aneurysmal SMCs (FC = −2.3)
miR-1	21	Unchanged	Aortic tissue	SMCs	Not specified	Down-regulated in TAA, no change in AAA
miR-1	33	Down-regulation	Aortic tissue	Not specified	Not specified	Down-regulated in AAA tissue (−4.8 = FC)
miR-1-3p	42	Down-regulation	Aortic tissue	HASMCs	miR-1-3p/TLR4 axis	Down-regulated in AAA tissues and Ang II-induced HASMCs, negatively correlated with TLR4 expression and AAA diameter, the rs4591246 AA genotype reduces miR-1-3p expression, promotes TLR4 expression, and HASMC phenotypic switching.
miR-1207-5p	30	Unchanged	Aortic tissue	Infrarenal aorta cells	Not specified	Up-regulated in qPCR array but not validated by qRT-PCR
miR-1207-5p	29	Down-regulation/Up-regulation	Aortic tissue	M1/M2 macrophages	Not specified	Down-regulated in aneurysmal SMCs (FC = −0.74); Up-regulated in M1 (18-fold) and M2 (1.1-fold)
miR-125b-5p	24	Down-regulation	Aortic tissue	SMCs	MAPK signaling	Down-regulated in A+ positive media, targets MMP13
miR-125b-5p	30	Down-regulation	Aortic tissue	Infrarenal aorta cells	Eicosanoid synthesis, metalloprotease/TIMP	Down-regulated in AAA, paired interaction with ALOX5
miR-126	21	Up-regulation	Aortic tissue	SMCs	Angiogenesis	Up-regulated in TAA and AAA
miR-133a	21	Unchanged	Aortic tissue	SMCs	Not specified	Down-regulated in TAA, no change in AAA
miR-133a	33	Down-regulation	Aortic tissue	SMCs	Not specified	Down-regulated in AAA tissue (−4.4 = FC)
miR-145	21	Down-regulation	Aortic tissue	SMCs	VEGF signaling	Down-regulated in AAA
miR-145	26	Up-regulation	Aortic tissue	SMCs	Not specified	Up-regulated in AAA-SMCs compared to non-aneurysmal SMCs
miR-145	3	Up-regulation	Aortic tissue	VSMCs	NRF2/miR-145/MYOCD axis	Up-regulated in AAA group and NRF2-positive VSMCs, positively correlated with NRF2, promotes expression of contractile biomarker genes (α-SMA, CNN1, SM22α) via MYOCD, NRF2 silencing with miR-145 inhibition decreases these genes, while miR-145 overexpression up-regulates them
miR-146a	21	Up-regulation	Aortic tissue	SMCs	Inflammation	Up-regulated in AAA
miR-146a-5p	6	Up-regulation	Aortic tissue	SMCs	miR-146a-5p/TRAF6 axis	Up-regulated in AAA aortas and plasma, PTE treatment enhances expression, inhibits macrophage pyroptosis and AAA formation by targeting TRAF6, miR-146a-5p knockout reverses PTE effects
miR-146a-5p	33	Up-regulation	Aortic tissue	SMCs	Not specified	Up-regulated in AAA tissue (5.8-fold)
miR-150-5p	30	Up-regulation	Infrarenal aortic tissue	Infrarenal aorta close to the aortic bifurcation cells	Eicosanoid synthesis, metalloprotease/TIMP	Up-regulated in AAA, paired interaction with CX3CL1
miR-155	14	Up-regulation	Aortic tissue	AAA neck and body cells	CTLA4 and SMAD2, TGF-β signaling	miR-155 was up-regulated in the aneurysm body (3.26-fold) compared with the neck, accompanied by down-regulation of its target genes CTLA4 and SMAD2, involved in immune regulation and TGF-β signaling.
miR-155	21	Down-regulation	Aortic tissue	SMCs	Inflammation	Down-regulated in TAA and AAA
miR-15a-3p	15	Down-regulation	Aortic tissue	abdominal aorta cells, focusing on adventitial tertiary lymphoid organs	Angiogenesis	Down-regulated in ATLOs, regulates VEGF (FC = −3.32)
miR-15a-5p	22	Up-regulation	Aortic tissue	VSMCs	CDKN2B	Overexpressed in AAA cells; negatively regulates CDKN2B, promoting VSMCs apoptosis; potential therapeutic target for AAA.
miR-15a	44	Up-regulation	Aortic tissue	Medial layer SMCs	Tissue-localized AAA	Expression is significantly associated with AAA diameter; localized in smooth muscle cells of the medial layer and vasa vasorum
miR-16-5p	30	Unchanged	Infrarenal aortic tissue	Infrarenal aorta cells close to the aortic bifurcation	Not specified	Up-regulated in qPCR array but not validated by qRT-PCR
miR-185-5p	40	Down-regulation	Aortic tissue	HASMCs	GAS5/miR-185-5p/ADCY7 axis	Down-regulated in AAA tissues and ANGII-induced HASMCs, promotes proliferation and inhibits apoptosis and inflammatory response in HASMCs, targets ADCY7, sponged by lncRNA GAS5, negatively correlated with GAS5 and ADCY7 expression, regulates AKT signaling pathway.
miR-195	23	Up-regulation	Aortic tissue	Aortic media cells	TGF-β signaling	Up-regulated, negatively regulates Smad3, increases OPN/collagen III, inhibits VSMCs proliferation, promotes apoptosis
miR-191-5p	43	Up-regulation	Aortic tissue	VSMCs	MIR503HG/miR-191-5p/PLCD1 axis	Up-regulated miR-191-5p promotes VSMCs apoptosis, ECM disruption, and inflammation by targeting PLCD1
miR-204	21	Down-regulation	Aortic tissue	SMCs	Not specified	Down-regulated in TAA and AAA
miR-204	32	Down-regulation	Aortic tissue	Exosomes from aortic cells	Not specified	Down-regulated in AAA plasma and tissue exosomes
miR-204-5p	24	Down-regulation	Aortic tissue	SMCs	Not specified	Down-regulated in A+ positive media, targets RUNX2, HAPLN1
miR-21	12	Up-regulation	Aortic tissue	SMCs, endothelial cells, fibroblasts	miR-21/PTEN/NF-κB axis	miR-21 was significantly Up-regulated in AAA tissues (6.7-fold in nonsmokers; 12.8-fold in smokers) compared with controls, with corresponding PTEN down-regulation
miR-21-3p	18	Down-regulation	Aortic tissue	VSMCs	Inflammation	Down-regulated, inflammation-dependent
miR-21-5p	21	Unchanged	Aortic tissue	SMCs	Inflammation	Up-regulated in TAA, no change in AAA
miR-21-5p	33	Up-regulation	Aortic tissue	SMCs	Not specified	Up-regulated in AAA tissue (1.9-fold, p = 0.012)
miR-21	29	Up-regulation	Aortic tissue	M1/M2 macrophages	Not specified	Up-regulated in aneurysmal SMCs (2.15-fold), M1 (5.7-fold), and M2 (8.9-fold)
miR-24	29	Up-regulation	Aortic tissue	M1/M2 macrophages	Not specified	Up-regulated in aneurysmal SMCs (1.6-fold), M1 (2.6-fold), and M2 (4.1-fold).
miR-24-3p	28	Down-regulation	Aortic tissue	SMCs	Not specified	Down-regulated in large AAA
miR-24	32	Down-regulation	Aortic tissue	Exosomes from aortic cells	Not specified	Down-regulated in AAA plasma and tissue exosomes
miR-24	13	Down-regulation	Aortic tissue	SMCs, macrophages	Inflammation/Apoptosis/ECM remodeling/miR-23b-24-27b cluster regulation	Significantly decreased in human AAA tissue; expression inversely correlated with CHI3L1, which increases with disease severity.
miR-27a-3p	28	Down-regulation	Aortic tissue	SMCs	Not specified	Down-regulated in large AAA
miR-29	21	Unchanged	Aortic tissue	SMCs	Inflammation	Up-regulated in TAA, no change in AAA
miR-29	32	Up-regulation	Aortic tissue	Exosomes from aortic cells	Not specified	Up-regulated in AAA plasma and tissue exosomes
miR-29a	11	Unchanged	Infrarenal aortic tissue	HASMCs, hAFBs	Collagen and ECM regulation	No significant change in human AAA
miR-29b	11	Down-regulation	Infrarenal aortic tissue	HASMCs, hAFBs	Collagen and ECM regulation	Down-regulated in human AAA and murine models (PPE and AngII), negatively correlated with COL1A1, COL3A1, COL5A1, ELN; regulates MMP2, MMP9
miR-29c	11	Down-regulation	Infrarenal aortic tissue	HASMCs, hAFBs	Collagen and ECM regulation	Down-regulated at 7 days in the PPE model, not significantly altered in human AAA
miR-29a	29	Up-regulation	Aortic tissue	M1/M2 macrophages	Not specified	Up-regulated in aneurysmal SMCs (1.6-fold), M1 (2.2-fold), and M2 (17.3-fold)
miR-29b	29	Up-regulation	Aortic tissue	M1/M2 macrophages	Not specified	Up-regulated in aneurysmal SMCs (1.6-fold), M1 (2.6-fold), and M2 (4.2-fold); no significant correlation with AAA diameter in plasma
miR-29c	29	Up-regulation	Aortic tissue	M1/M2 macrophages	Not specified	Up-regulated in M1 (1.85-fold) and M2 (8.5-fold)
miR-30	21	Unchanged	Aortic tissue	Not specified	Not specified	No change in AAA
miR-30a-5p	15	Down-regulation	Aortic tissue	abdominal aorta, focusing on adventitial tertiary lymphoid cells	Inflammation	Down-regulated in ATLOs, linked to cytokine signaling (−2.3 = FC)
miR-30d-5p	28	Down-regulation	Aortic tissue	SMCs	Not specified	Down-regulated in large AAA
miR-30d-5p	39	Down-regulation	Aortic tissue	HVSMCs	NEAT1/miR-30d-5p/ADAM10 axis	Down-regulated in H2O2-treated HVSMCs, promotes proliferation and inhibits apoptosis in HVSMCs, targets ADAM10, sponged by lncRNA NEAT1, and is negatively correlated with NEAT1 and ADAM10 expression
miR-30a-5p	29	Down-regulation	Aortic tissue	M1/M2 macrophages	Inflammation	Potential biomarker in plasma
miR-326	28	Up-regulation	Aortic tissue	SMCs	Not specified	Up-regulated in small and large AAA
miR-33	31	Down-regulation	Aortic tissue	AAA body cells	Lipid metabolism	Exclusive to the AAA body, regulates cholesterol
miR-33	32	Up-regulation	Aortic tissue	Exosomes from aortic tissue cells	Not specified	Up-regulated in AAA plasma and tissue exosomes
miR-331-3p	21	Down-regulation	Aortic tissue	SMCs	Not specified	Down-regulated in AAA
miR-486-5p	21	Up-regulation	Aortic tissue	SMCs	Not specified	Up-regulated in TAA and AAA
miR-99b-5p	24	Down-regulation	Aortic tissue	SMCs	Not specified	Down-regulated in A+ positive media

Acronyms: AAA: Abdominal Aortic Aneurysm; A+: AAA with positive FDG uptake; AngII: Angiotensin II; ATLOs: Adventitial Tertiary Lymphoid Organs; CDKN2B: Cyclin Dependent Kinase Inhibitor 2B; CHI3L1: Chitinase-3-like protein 1; COL1A1: Collagen Type I Alpha 1 Chain; COL3A1: Collagen Type III Alpha 1 Chain; COL5A1: Collagen Type V Alpha 1 Chain; CTLA4: Cytotoxic T-Lymphocyte–Associated Protein 4; ECM: Extracellular Matrix; ELN: Elastin; FC: Fold Change; GAS5: Growth Arrest Specific 5 (lncRNA); HASMCs: Human Aortic Smooth Muscle Cells; hAFBs: Human Aortic Adventitial Fibroblasts; MMP: Matrix Metalloproteinase; MYOCD: Myocardin; NF-κB: Nuclear Factor kappa-light-chain-enhancer of activated B cells; NRF2: Nuclear Factor Erythroid 2–Related Factor 2; OPN: Osteopontin; PBMCs: Peripheral Blood Mononuclear Cells; PPE: Porcine Pancreatic Elastase (AAA model); PTEN: Phosphatase and Tensin Homolog; PTE: Puerarin; RUNX2: Runt-Related Transcription Factor 2; SIRT1: Sirtuin 1; SM22α: Smooth Muscle Protein 22 Alpha; SMAD2: Mothers Against Decapentaplegic Homolog 2; SMCs: Smooth Muscle Cells; TIMP: Tissue Inhibitor of Metalloproteinases; TAA: Thoracic Aortic Aneurysm; TGF-β: Transforming Growth Factor Beta; TLR4: Toll-Like Receptor 4; TRAF6: TNF Receptor–Associated Factor 6; VEGF: Vascular Endothelial Growth Factor; VSMCs: Vascular Smooth Muscle Cells; α-SMA: Alpha–Smooth Muscle Actin; HVSMCs: Human VSMCs.

While numerous miRNAs showed differential expression, consistency across studies was limited. The most reproducibly up-regulated miRNAs in aortic tissue included miR-21 (reported up-regulated in multiple studies with fold changes up to 12.8 in smokers, linked to PTEN/NF-κB axis and inflammation/ECM remodeling), miR-146a/miR-146a-5p (consistent up-regulation, 5.8-fold in one study, targeting TRAF6 and modulating macrophage pyroptosis), and members of the miR-15/16 family in specific contexts. Down-regulated candidates with reasonable reproducibility included miR-204/miR-204-5p, miR-24-3p/miR-24, miR-27a-3p, miR-125b-5p, and certain let-7 and miR-29 family members, often associated with impaired ECM homeostasis and VSMC dysfunction. Conflicting findings were common. miR-1, miR-133a, miR-145, miR-155, and miR-29 isoforms showed directionally opposite or null results depending on the study, possibly due to differences in tissue sampling site (aneurysmal body vs. neck), cell-type specificity (bulk tissue vs. LCM-isolated macrophages or SMCs), or patient comorbidities. Such inconsistencies highlight the context-dependent nature of miRNA regulation in AAA and underscore the importance of spatial and cellular resolution in future profiling efforts. Overall, miR-21 and miR-146a emerged as the most consistently dysregulated in tissue, with plausible mechanistic links to core AAA pathophysiology.

### 3.4 miRNAs differentially expressed in circulating blood of AAA patients and their potential biological roles

Members of the let-7 family exhibited marked heterogeneity across circulating blood compartments. let-7b-5p was down-regulated in plasma [34], but up-regulated in serum [5]. let-7g-3p was down-regulated in PBMCs [36], while let-7i-5p showed reduced levels in plasma [19]. let-7e was likewise decreased in whole-blood leukocytes [16].

On the ither hand, miR-1-3p was down-regulated in serum [41]. miR-125b showed consistent down-regulation across PBMCs [36], and plasma [24]. Conversely, miR-1260a and miR-1281 were up-regulated in plasma [17,33], with miR-1281 further elevated in peripheral venous blood before EVAR and markedly reduced after repair [37].

Several miRNAs demonstrated reproducible down-regulation in plasma, including miR-133b, miR-150-5p, miR-27a-3p, miR-29a-3p, and miR-331-3p [34]. Pro-inflammatory miR-142-5p was up-regulated in plasma, and miR-146a was increased in serum [24,35], with its isoform miR-146a-5p also elevated in plasma [33]. miR-155 was consistently up-regulated in both serum and RBC-enriched whole blood [14,20]. The miR-15/16 family displayed divergent patterns: miR-15a-3p was reduced in inflammatory and macrophage subsets [15]; miR-15a was down-regulated in whole-blood leukocytes [16] yet increased in plasma from patients with large AAAs [44]; and miR-16-5p was down-regulated in plasma [19].

miR-191 exhibited context-dependent regulation, being down-regulated in whole blood following repair [27] but up-regulated in serum [38], while miR-191-3p showed substantial elevation in plasma [17,43]. Strong up-regulation was also observed for miR-195 and miR-21 family members across serum, PBMCs and whole blood [2,25,36].

**Fig. 3:**
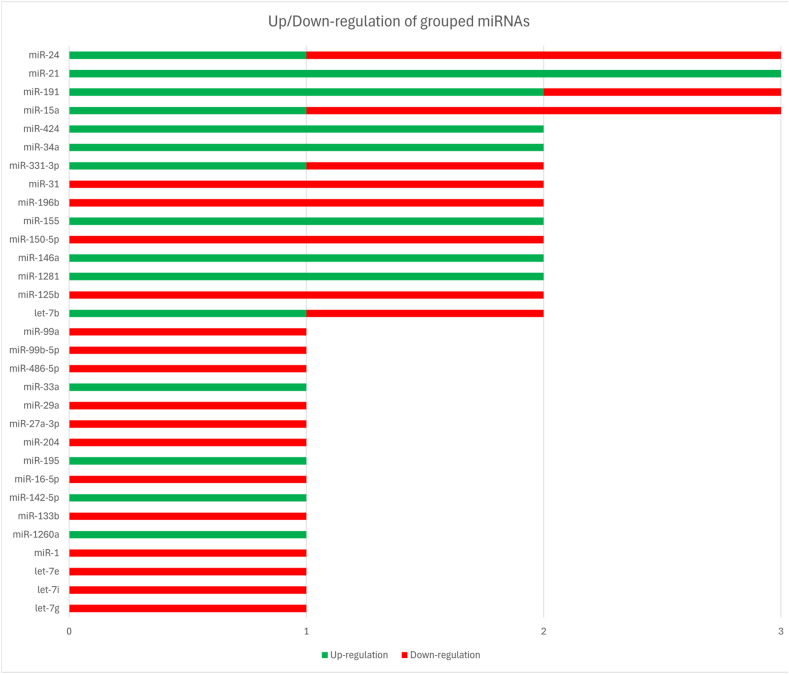
Stacked bar chart summarizing reported expression changes of grouped miRNAs in the blood/plasma. Variants (e.g., -3p/-5p, a/b/c) were merged into families. Green bars indicate the number of studies reporting up-regulation, while red bars represent down-regulation. The total bar length corresponds to the number of studies per miRNA group. (For interpretation of the references to colour in this figure legend, the reader is referred to the Web version of this article.)

miR-24 displayed compartment-specific behavior, being down-regulated in plasma [13]and serum [4], whereas miR-24-3p was modestly increased in PBMCs [36](Table 3). These findings are visually represented in Fig. 3, which highlights the distribution of up- and down-regulation across miRNA families.

**Table 3:** miRNA Expression Profiles in Blood Samples from AAA Patients.

miRNA	Reference	Direction	Sample	Sample cells	Pathway	Keynote
let-7b-5p	34]	Down-regulation	Plasma	-	Not specified	Down-regulated in SAD and AAA plasma, previously associated with aortic aneurysms
let-7b-5p	[5]	Up-regulation	Serum	-	Morphogenesis and cellular response to endogenous stimuli	Up-regulated in AAA serum, positively correlated with aortic diameter, associated with AAA diagnosis (OR 13.06), improves diagnostic AUC with miR-548n to 98.0%
let-7g-3p	36]	Down-regulation	Peripheral blood	PBMCs	Not specified	FC = −0.42, AUC = 0.887
Let-7i-5p	19]	Down-regulation	Plasma	-	Not specified	Fold Change = −1.3, AUC = 0.74, Specificity = 40%
let-7e	16]	Down-regulation	Peripheral blood	Whole blood leukocytes	Inflammation/adhesion molecule regulation	Down-regulated (FC = −1.80) let-7e Regulates VCAM1, ICAM1, and inflammatory pathways related to AAA and atherosclerosis
miR-1-3p	41]	Down-regulation	Serum	-	Negative regulation of apoptosis, sprouting angiogenesis, angiogenesis, positive regulation of endothelial cell migration and proliferation, and regulation of cell shape	Down-regulated in AAA serum, negatively correlated with WBC, CRP, and AAA image parameters (diameter, area, volume), low levels (≤0.62) associated with increased AAA risk, involved in the negative regulation of apoptosis, angiogenesis, and cell proliferation
miR-125b	36]	Down-regulation	Peripheral blood	PBMCs	Not specified	FC = −0.86, AUC = 0.868
miR-125b-5p	24]	Down-regulation	Plasma	-	MAPK signaling	Down-regulated in A+ plasma, Down-regulated in A+ positive media, targets MMP13
miR-1260a	33]	Up-regulation	Plasma	-	Not specified	Overexpressed in AAA plasma (fold change 1.49-2), poor expression in tissue
miR-1281	17]	Up-regulation	Plasma	-	Not specified	Significantly elevated (fold change >5); potential biomarker for AAA detection
miR-1281	37]	Up-regulation	Peripheral venous blood	-	Not specified	Significantly elevated in AAA before EVAR (1.66-fold) and reduced after EVAR (0.27-fold); independent of creatinine, cholesterol, and CRP.
miR-133b	34]	Down-regulation	Plasma	-	Not specified	Down-regulated in SAD and AAA plasma, previously associated with aortic aneurysms
miR-142-5p	24]	Up-regulation	Plasma	-	TGF-β signaling	Up-regulated in A+ plasma
miR-146a	35]	Up-regulation	Serum	-	NF-kB pathway via CARD10, SIRT1, p65, MMP-2, MMP-9	Up-regulated in AAA serum, PBMCs, and aortic tissue; negatively correlated with TNF-α, IFN-γ, CRP, CARD10 mRNA, MMP-9 mRNA, MMP-2 mRNA; positively correlated with IL-10; inhibits NF-kB pathway and MMP-2/MMP-9 expression; reduces TNF-α-induced HUVEC apoptosis
miR-146a-5p	33]	Up-regulation	Plasma	-	Not specified	Up-regulated in plasma (fold change 1.49-2)
miR-150-5p	34]	Down-regulation	Plasma	-	Not specified	Down-regulated in SAD and AAA plasma, negatively correlated with age, and related to AAA molecular mechanisms.
miR-150-5p	36]	Down-regulation	Peripheral blood	PBMCs	Not specified	FC = −0.78, AUC = 0.906
miR-155	14]	Up-regulation	Serum	Without residual cell debris	Inflammation	miR-155 was Up-regulated 2.67-fold in AAA patients compared with controls, though this was borderline significant.
miR-155	20]	Up-regulation	Blood	RBC	Inflammation	Up-regulated, correlated with age and tumor size, linked to prognosis
miR-15a-3p	15]	Down-regulation	Blood/Plasma	Inflammatory cells: neutrophils, B and T lymphocytes, and mast cells. Macrophages: M1 (proinflammatory) and M2 (anti-inflammatory).	Angiogenesis	Down-regulation of miR-15a-3p in M1 (FC = −1.0) and M2 (FC = −1.74) macrophages.
miR-15a	16]	Down-regulation	Peripheral blood	Whole blood leukocytes	Inflammatory and vascular remodeling pathways	Down-regulated (FC −2.24) Regulates CRP, IL-6, and TNF-α signaling involved in AAA progression.
miR-15a	44]	Up-regulation	Plasma	-	apoptosis, inflammation, and mesenchymal changes	Higher levels are associated with larger AAA; levels positively correlated with AAA diameter
miR-16-5p	19]	Down-regulation	Plasma	-	Not specified	Fold Change = −1.5, AUC = 0.71, Specificity = 47%
miR-191	27]	Down-regulation	Whole blood	-	MMPs, IL-6, TNF-α	Down-regulated post-repair, interacts with SATB1, CDK6, VCAM1, ICAM1, TIMP1, MMPs, IL-6, TNF-α, DAB2IP, SERPINA1, LDLR, IL-16
miR-191	38]	Up-regulation	Serum	-	Cell adhesion, metal-binding metallothioneins, immune activation, interleukin signaling, DNA methylation, extracellular matrix metabolism	Up-regulated in AAA patient serum, induces significant changes in 1492 protein-coding genes in endothelial cells, 17 Down-regulated genes identified as direct targets, and affects multiple biological pathways
miR-191-3p	17]	Up-regulation	Plasma	-	Not specified	Significantly elevated (fold change >5); potential diagnostic biomarker for AAA
miR-195	25]	Up-regulation	Blood	-	PI3K/Akt signaling	Up-regulated in AAA blood, promotes IL-1β, IL-6, MMP-2, MMP-9, TNF-α, NF-κB; suppresses VEGF, PI3K, p-Akt
miR-196b	16]	Down-regulation	Whole blood	-	A1AT, MTHFR, and OPG	Down-regulated (FC −2.26); linked to vascular inflammation and matrix regulation
miR-196b	16]	Down-regulation	Plasma	-	Inflammation	Down-regulated (FC −3.75) in plasma, not significant after regression
miR-204-5p	24]	Down-regulation	Plasma	-	Not specified	Down-regulated in A+ plasma, Down-regulated in A+ positive media, targets RUNX2, HAPLN1
miR-21-5p	36]	Up-regulation	Peripheral blood	PBMCs	Not specified	High expression (Fold Change = 1.356, AUC = 0.953)
miR-21-3p	36]	Up-regulation	Peripheral blood	PBMCs	Not specified	Fold Change = 1.704, AUC = 0.919
miR-21	[2]	Up-regulation	Whole blood	WBC, RBC	Not specified	Elevated 2.76-fold in AAA patients preoperatively compared to controls, reduced expression 6 months post-endovascular repair, no significant change in expression in patients with endoleaks
miR-24	13]	Down-regulation	Plasma	-	Not specified	Decreased circulating levels in AAA patients vs. controls; no difference between small and large AAA; inversely correlated with CHI3L1 plasma levels.
miR-24	[4]	Down-regulation	Serum	-	Not specified	Down-regulated in small AAAs (FC = −1.89) and large AAAs (FC = −2.74) compared to controls.
miR-24-3p	36]	Up-regulation	Peripheral blood	PBMCs	Not specified	Up-regulated (Fold Change = 1.143, AUC = 0.874)
miR-27a-3p	34]	Down-regulation	Plasma	-	Not specified	Down-regulated in SAD and AAA plasma, previously associated with aortic aneurysms
miR-29a-3p	34]	Down-regulation	Plasma	-	Not specified	Down-regulated in SAD and AAA plasma, previously associated with aorta aneurysms
miR-31-5p	36]	Down-regulation	Peripheral blood	PBMCs	Not specified	FC = −1.54, AUC = 0.981
miR-31-3p	36]	Down-regulation	Peripheral blood	PBMCs	Not specified	FC = −1.60, AUC = 0.970
miR-331-3p	34]	Down-regulation	Plasma	-	Not specified	Down-regulated in SAD and AAA plasma, previously associated with aortic aneurysms
miR-33a-5p	19]	Up-regulation	Plasma	-	Not specified	Fold Change = 1.8, AUC = 0.79, Specificity = 23%
miR-331-3p	19]	Up-regulation	Plasma	-	Not specified	Fold Change = 1.6, AUC = 0.77, Specificity = 19%
miR-34a-5p	36]	Up-regulation	Peripheral blood	PBMCs	Not specified	Fold Change = 2.188, AUC = 0.927
miR-34a-3p	36]	Up-regulation	Peripheral blood	PBMCs	Not specified	Fold Change = 2.357, AUC = 0.867
miR-424-3p	36]	Up-regulation	Peripheral blood	PBMCs	Not specified	Fold Change = 1.872, AUC = 0.861
miR-424-5p	36]	Up-regulation	Peripheral blood	PBMCs	Not specified	Fold Change = 1.579, AUC = 0.810
miR-486-5p	24]	Down-regulation	Plasma	-	Not specified	Down-regulated in A+ plasma
miR-99b-5p	24]	Down-regulation	Plasma	-	Not specified	Down-regulated in A+ plasma, also Down-regulated in A+ positive media
miR-99a-3p	36]	Down-regulation	Peripheral blood	PBMCs	Not specified	FC = −1.06, AUC = 0.853

Acronyms: AAA: Abdominal Aortic Aneurysm; A+: Aortic positive; AUC: Area Under the Curve; CRP: C-reactive protein; EVAR: Endovascular Aneurysm Repair; FC: Fold Change; HUVEC: Human Umbilical Vein Endothelial Cells; ICAM1: Intercellular Adhesion Molecule 1; M1/M2: Macrophage subtypes (M1 = pro-inflammatory, M2 = anti-inflammatory); MMP: Matrix Metalloproteinase; NF-κB: Nuclear Factor kappa-light-chain-enhancer of activated B cells; PBMCs: Peripheral Blood Mononuclear Cells; SMCs: Smooth Muscle Cells; SATB1: Special AT-rich sequence-binding protein 1; TGF-β: Transforming Growth Factor Beta; TIMP1: Tissue Inhibitor of Metalloproteinases 1; VCAM1: Vascular Cell Adhesion Molecule 1; WBC: White Blood Cells; RBC: Red Blood Cells; VSMCs: Vascular Smooth Muscle Cells; lncRNA: Long Non-coding RNA.

Reproducibility was generally lower in circulating compartments than in tissue, reflecting additional technical (biospecimen type, normalization) and biological (systemic vs. local spillover) variability. Promising diagnostic candidates with repeated up-regulation included miR-21, miR-146a, miR-155, miR-1281, and miR-34a, while miR-150-5p, miR-27a-3p, miR-29 family, miR-125b, and miR-331-3p were more frequently down-regulated. Dynamic changes post-EVAR (e.g., decreased miR-1281 and miR-191) suggest potential for monitoring treatment response. Notable reproducible signals with reported diagnostic metrics include miR-21 family members (AUC values > 0.9 in PBMCs in one study), miR-150-5p (AUC 0.906), and combinations such as let-7b-5p with miR-548n (AUC 0.98). However, many AUCs were derived from small cohorts without external validation, and specificity was often modest. The most clinically relevant candidates appear to be those with consistent directionality across independent studies and plausible correlation with AAA diameter or growth (e.g., miR-15a-5p, miR-1281).

### 3.5 Quality assessment

**Selection domain:** All studies (100%, 39/39) were rated low risk for case definition, indicating that AAA cases were consistently defined using established diagnostic criteria. In contrast, only 6 studies (15%) were considered low risk for representativeness, while the majority (85%, 33/39) were rated moderate risk, mainly due to single-center recruitment, non-consecutive enrollment, or unclear sampling strategies. The selection and definition of controls were uniformly judged low risk across all studies (100%, 39/39), reflecting clearly defined and appropriately matched control groups.

**Comparability domain:** Assessment of confounding showed that 27 studies (69%) adequately controlled for key variables, including age, sex, smoking, and comorbidities. Eight studies (21%) were rated moderate risk due to partial adjustment, and four studies (10%) were rated high risk for insufficient control of major confounders. These patterns suggest that residual confounding may have influenced some reported associations between miRNA expression and AAA.

**Fig. 4:**
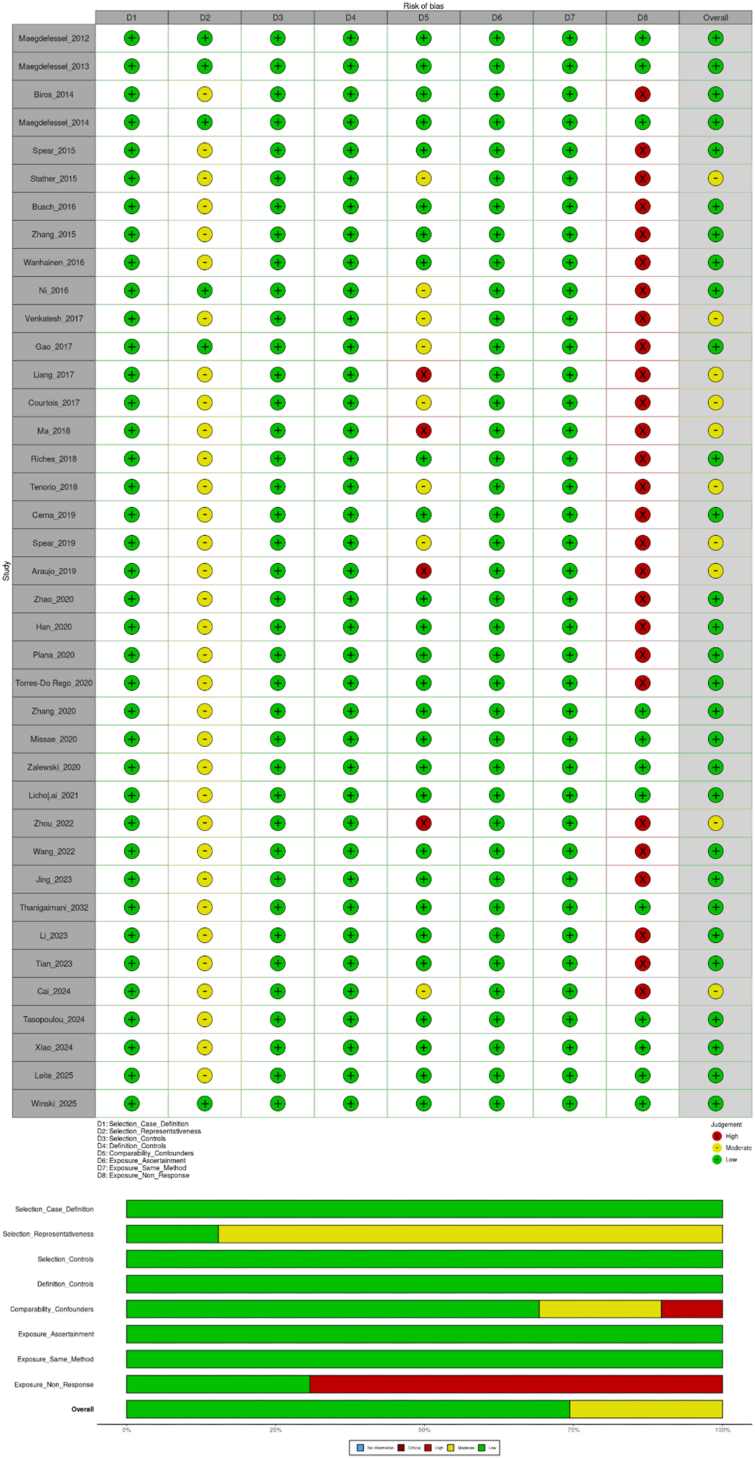
Quality assessment using NOS tool.

**Exposure domain:** All studies (100%, 39/39) were rated low risk for ascertainment of exposure and consistent application of measurement methods across cases and controls, indicating methodological uniformity in PCR-, microarray-, or sequencing-based assays. However, non-response bias remained a major limitation, with 27 studies (69%) rated high risk due to incomplete reporting of sample processing, attrition, or missing data. This highlights persistent gaps in transparency and reporting quality (Fig. 4). Supplementary Table 3 provides detailed NOS scoring for all included studies.

## 4 Discussion

This review identified several recurrent miRNA signals in AAA; however, interpretation should remain cautious because the available evidence is heterogeneous, largely observational, and frequently based on small single-center cohorts. The studies included in this review demonstrated substantial heterogeneity in sample sources, control definitions, screening platforms, and validation methods, highlighting the need for standardized approaches to improve comparability and reliability in miRNA research on aneurysm disease. Differences in plasma versus serum processing, hemolysis control, endogenous normalization references, and PCR platform calibration likely contributed substantially to between-study variability.

Several miRNAs, including miR-21, miR-133, miR-29b, and miR-155, were consistently reported to be differentially expressed in aortic tissue, with recurrent patterns of up-regulation or down-regulation linked to key pathological pathways such as extracellular matrix remodeling, inflammation, and modulation of smooth muscle cell phenotype. Circulating miRNAs showed greater variability, with only a subset overlapping with tissue findings, suggesting that some miRNAs may serve as systemic biomarkers of aneurysm-related pathology. In contrast, others act locally within the aneurysmal wall. These observations underscore the potential of specific miRNAs for diagnostic applications and highlight their mechanistic relevance to disease progression and therapeutic targeting.

### 4.1 Pathways modulated by miRNAs in AAA

MicroRNAs play pivotal roles in the pathogenesis of AAA by regulating key signaling pathways involved in inflammation, ECM remodeling, angiogenesis, modulation of SMCs phenotype, and other cellular processes. These pathways not only underpin AAA development and progression but also highlight miRNAs' potential as diagnostic and prognostic biomarkers.

Several miRNAs repeatedly identified in this systematic review have been implicated in key biological pathways central to AAA pathogenesis, including inflammation, ECM remodeling, VSMC phenotypic switching, and apoptosis. However, the majority of mechanistic insights derive from associative correlations observed in human AAA tissues and biofluids rather than direct causal evidence in patients. Nevertheless, these associations remain largely correlative. Direct causal relationships in humans are difficult to establish due to the observational nature of the included studies and ethical constraints on interventional designs. Most functional validation originates from in vitro experiments using HASMCs or ex vivo tissue explants. With this caveat in mind, the following sections synthesize the most consistently reported miRNA-pathway associations supported by human AAA samples.

#### 4.1.1 Inflammation-related pathways

Inflammation is a cornerstone of AAA pathology, characterized by immune cell infiltration, cytokine release, and activation of pro-inflammatory signaling cascades. Several miRNAs modulate these processes, often exhibiting dysregulated expression in AAA samples.

• **NF-κB Pathway**: miR-146a and miR-146a-5p are key regulators, up-regulated in both aortic tissue and circulating blood (serum, plasma, PBMCs). In tissue, miR-146a-5p targets TRAF6, inhibiting macrophage pyroptosis and attenuating AAA formation [6]. In circulation, miR-146a negatively correlates with pro-inflammatory cytokines (TNF-α, IFN-γ) and MMPs (MMP-2, MMP-9), while positively correlating with anti-inflammatory IL-10. This suggests a feedback mechanism to dampen NF-κB-mediated inflammation, with diagnostic implications (e.g., elevated levels as biomarkers of active inflammation) [17].
• **TGF-β Signaling**: Up-regulated miR-155 in serum/red blood cells promotes TGF-β-driven inflammation and T-cell infiltration [14]. Similarly, miR-195, up-regulated in tissue and serum, enhances TGF-β signaling, increasing pro-inflammatory cytokines (IL-1β, IL-6, TNF-α) and ECM-degrading enzymes [25]. In contrast, miR-142-5p, up-regulated in plasma, modulates TGF-β signaling, potentially exacerbating inflammatory cross-talk via interactions with IL6, MMP9, and CCL2 in ceRNA networks (ceRNA networks modulate gene expression by using lncRNAs and circRNAs to sponge miRNAs) [24].
• **General Inflammatory Modulation**: Down-regulated miR-196b in whole blood and plasma is linked to inflammation in AAA and PAD, indicating a protective role lost in disease states [16].

These inflammation-modulating miRNAs, with their consistent dysregulation patterns, offer prognostic value, as their levels correlate with AAA severity and could predict rupture risk through inflammatory burden assessment.

#### 4.1.2 ECM remodeling and matrix degradation pathways

ECM degradation, driven by metalloproteinases and the loss of structural proteins such as collagen and elastin, is central to aortic wall weakening in AAA. miRNAs frequently target genes involved in ECM homeostasis.

• **Collagen and ECM Regulation**: The miR-29 family (miR-29a, miR-29b, miR-29c) exhibits context-dependent regulation. In tissue, miR-29b and miR-29c are down-regulated in infrarenal AAA and murine models, negatively correlating with COL1A1, COL3A1, COL5A1, and ELN, while regulating MMP2 and MMP9 [11]. Up-regulation in exosomes and tissue suggests stage-specific roles, with potential as biomarkers for early ECM disruption [32]. miR-29b/c down-regulated in tissue links to ECM remodeling via regulation of collagens and MMPs [29].
• **MAPK Signaling and Metalloproteinase Regulation**: Down-regulated miR-125b-5p in tissue and plasma targets MMP13 and engages MAPK signaling, contributing to inflammatory matrix degradation via interactions with ALOX5. Similarly, miR-204-5p down-regulated in tissue and plasma, targets RUNX2 and HAPLN1, influencing ECM dynamics and vascular calcification [24].
• **Eicosanoid Synthesis and Metalloprotease/TIMP Balance**: Up-regulated miR-150-5p in tissue pairs with CX3CL1 [30], while down-regulated miR-125b-5p interacts with ALOX5, both affecting eicosanoid synthesis and metalloprotease activity, pivotal for ECM turnover [30].

Circulating levels of miRNAs like miR-29b/c are up-regulated in aortic tissue [29], may complement tissue changes [11], supporting their use in non-invasive monitoring of ECM integrity.

#### 4.1.3 Angiogenesis and vascular remodeling pathways

Angiogenesis contributes to AAA instability by promoting neovascularization and further inflammation. miRNAs regulate VEGF and related pathways.

• **VEGF Signaling**: Up-regulated miR-145 in tissue and cultured SMCs links to VEGF, with dual roles, protective in NRF2-positive VSMCs via the NRF2/miR-145/MYOCD axis, promoting contractile genes (α-SMA, CNN1, SM22α) [3], yet down-regulated in bulk AAA tissue, indicating lost homeostasis [21]. Down-regulated miR-15a-3p in tissue and plasma regulates VEGF and angiogenesis, positioning it as a potential plasma biomarker [15].
• miR-126 is up-regulated in aortic tissues of TAA and AAA, promoting angiogenesis and influencing vascular smooth muscle cell function [21].

#### 4.1.4 SMCs phenotype modulation and apoptosis pathways

SMCs phenotypic switching from contractile to synthetic states drives AAA progression, often accompanied by apoptosis.

• **TLR4 Axis**: Down-regulated miR-1-3p in tissue and HASMCs negatively correlates with TLR4 and AAA diameter, promoting HASMC switching and apoptosis regulation [42]. In serum, its down-regulation correlates with inflammation and AAA parameters, enhancing its prognostic utility [41].
• **NEAT1/miR-30d-5p/ADAM10 Axis**: Down-regulated miR-30d-5p in tissue and peripheral blood targets ADAM10, promoting SMCs proliferation and inhibiting apoptosis, sponged by lncRNA NEAT1 [28,39].
• **GAS5/miR-185-5p/ADCY7 Axis**: Down-regulated miR-185-5p in tissue and HASMCs targets ADCY7, regulating AKT signaling to influence proliferation, apoptosis, and inflammation [40].
• **Apoptosis and Contractility**: Both miR-185-5p and miR-30d-5p are down-regulated in AAA tissues, promoting vascular smooth muscle cell proliferation while inhibiting apoptosis and inflammation. miR-185-5p functions via the GAS5/miR-185-5p/ADCY7 axis and regulates the AKT pathway, whereas miR-30d-5p acts through the NEAT1/miR-30d-5p/ADAM10 axis, with both showing negative correlations with their respective lncRNAs and targets [39,40].

#### 4.1.5 Other pathways: Lipid Metabolism, morphogenesis, and immune activation

• **Lipid Metabolism**: Down-regulated miR-33 suppresses inflammatory pathways, contributing to vascular lipid accumulation and inflammation by ABCA1 [31].
• **Morphogenesis and Cellular Response**: Up-regulated let-7b-5p in serum associates with morphogenesis, positively correlating with AAA diameter, improving diagnostic AUC [5].
• **PI3K/Akt Signaling**: Up-regulated miR-195 in serum promotes this pathway, enhancing cytokine production and ECM degradation [25].
• **Cell Adhesion and Immune Pathways**: Up-regulated miR-191 in serum affects cell adhesion, metallothioneins, and interleukin signaling, inducing transcriptomic changes in endothelial cells [38].

### 4.2 Diagnostic utility

Circulating miRNAs hold considerable promise as non-invasive biomarkers for the early detection and monitoring of AAA. Several miRNAs, including let-7, miR-125b, miR-150-5p, miR-31, and miR-331-3p, were consistently down-regulated in AAA plasma or serum, suggesting impaired vascular integrity and altered immune signaling [5,34,36]. In contrast, up-regulated miRNAs such as miR-21, miR-146a, miR-195, miR-1281, and miR-34a were associated with inflammation, extracellular matrix remodeling, and vascular smooth muscle cell apoptosis, indicating active aneurysmal progression [17,25,29,33,36]. Significantly, dynamic expression changes, such as reduced miR-1281 and miR-191 levels following endovascular repair, demonstrate the potential of circulating miRNAs as markers of treatment response and disease stabilization [27,37]. Collectively, these findings underscore that specific circulating miRNA signatures showed preliminary ability to distinguish AAA patients from controls in individual studies, although pooled diagnostic accuracy cannot be inferred from the current evidence base [19,36].

### 4.3 Therapeutic implications

Therapeutic implications remain hypothetical at present and are derived mainly from preclinical rather than clinical human studies. Although preclinical studies are promising, translating them into human AAA treatment remains limited by gaps in clinical validation and delivery challenges. Animal models, such as the porcine pancreatic elastase (PPE) and angiotensin II (AngII) models, demonstrate miRNA-mediated regulation of AAA progression. For example, miR-146a-5p's inhibition of macrophage pyroptosis via the TRAF6 axis in response to PTE treatment suggests a therapeutic target for reducing AAA formation [6]. Similarly, miR-145's modulation of contractile VSMCs genes (α-SMA, CNN1, SM22α) via the NRF2/MYOCD axis indicates potential for stabilizing vascular integrity [3]. miR-185-5p and miR-30d-5p, through their respective GAS5/ADCY7 and NEAT1/ADAM10 axes, promote VSMCs proliferation and inhibit apoptosis, offering avenues for mitigating AAA progression [39,40]. However, these findings are primarily derived from in vitro and animal studies, with limited human data. Challenges in miRNA delivery, specificity, and off-target effects exacerbate the clinical gap. For instance, miR-146a-5p knockout reverses therapeutic benefits, highlighting the need for precise modulation [6]. Additionally, the dual roles of miR-29b (up-regulated in aortic tissue [29], down-regulated in collagen regulation [11]) complicate therapeutic design. Future research should focus on developing targeted delivery systems, such as nanoparticle-based miRNA mimics or inhibitors, and conducting clinical trials to bridge the gap between preclinical promise and human therapeutic application.

### 4.4 Sources of heterogeneity in miRNA expression studies

Several methodological and design-related factors contributed to the elevated risk of bias observed in the majority of the included studies according to the NOS. Moderate or high risk of bias was most commonly attributed to inadequate representativeness of cases as the majority of studies were single-center with highly selected surgical patients (Biros et al., 2014; Cerna et al., 2019; Liang et al., 2017) incomplete adjustment for key confounders such as smoking status, statin use, and cardiovascular comorbidities, and high non-response bias due to poor reporting of sample collection protocols, hemolysis assessment, RNA quality control, or participant flow. These limitations were particularly problematic in miRNA biomarker research, given the high sensitivity of circulating miRNA levels to pre-analytical variables (blood collection tubes, processing delays, and normalization strategies). For instance, studies using U6 snRNA as the sole normalization control (Liang et al., 2017, Ma et al., 2018, Tenorio et al., 2018, Zhao et al., 2020) or those with unclear normalization methods (Gao et al., 2017) were more prone to technical variability. Consequently, studies with higher risk of bias frequently reported exaggerated fold changes or inconsistent directionality of miRNA dysregulation (such as conflicting findings for miR-155 and miR-29 family members), potentially leading to overestimation of the diagnostic accuracy or prognostic value of candidate miRNAs and contributing to poor reproducibility across cohorts. Inadequate control for comorbidities, as seen in several studies that provided limited or no details on past medical history (Maegdefessel 2012, Venkatesh 2017, Riches 2018), further complicates interpretation, as it remains unclear whether observed miRNA changes primarily reflect AAA-specific pathology or broader systemic inflammation and atherosclerosis common in this patient population. To overcome these issues and strengthen the evidence base, future studies should adopt multicenter designs with consecutive patient enrollment, implement rigorous multivariable adjustment for confounders, adhere to standardized reporting guidelines (such as MIQE for qRT-PCR), and establish pre-specified protocols for biospecimen processing and normalization. Ultimately, prospective validation in large-scale, low risk-of-bias studies such as the population-based approach used by Winski et al. (2025) will be essential to distinguish robust miRNA biomarkers from methodological artifacts.

In peripheral blood samples, inconsistencies stem from the differential distribution of miRNAs across components such as plasma, serum, whole blood, red blood cells, white blood cells, and exosomes, with variations in anticoagulants, RNA isolation methods, and normalization strategies further exacerbating discrepancies; for instance, plasma-derived miRNAs often reflect systemic changes, while cellular components like macrophages (M1/M2) may capture localized inflammatory signals, leading to non-reproducible findings as seen in variable expression [15]. Tissue-derived profiles frequently differ from blood-derived ones, with tissue samples showing up-regulation of miRNAs like miR-21 ^12,33^ and miR-146a [21] linked to local ECM remodeling [12] and inflammation [21]. In contrast, blood profiles highlight systemic markers such as down-regulated miR-24^4,13^ and up-regulated miR-1281 ^17,37^, potentially due to the compartmentalized nature of miRNA regulation (local aneurysmal microenvironment versus circulating spillover). Studies employing diverse control groups, such as organ donors for tissue (often limited by small sample sizes and comorbidities) [30] versus adjacent non-aneurysmal tissue or healthy volunteers for blood [20], introduce systematic biases; organ donor controls may include underlying pathologies not present in adjacent tissue, resulting in altered baseline miRNA levels and inconsistent dysregulation patterns, as evidenced by age and hypertension imbalances requiring statistical adjustments. Pathological heterogeneity is further compounded by tissue sampling sites, with the maximally dilated aneurysmal body (e.g., infrarenal close to bifurcation or AAA body) exhibiting more pronounced inflammatory and degradative miRNA signatures compared to the transitional zone (e.g., AAA neck) [30], where protective or early-stage profiles may predominate, as spatial variations in hemodynamics and cellular composition influence expression [14]. Comorbidities, prevalent in AAA cohorts, along with medications like statins, add layers of variability by modulating miRNA pathways independently of aneurysm pathology. This multifaceted heterogeneity undermines the robustness of current conclusions, potentially over- or underestimating miRNA roles in AAA pathogenesis and the utility of biomarkers.

## 5 Limitations

Despite the comprehensive nature of this review, several limitations should be acknowledged. First, the included studies exhibited considerable methodological variability regarding sample types, patient selection, detection platforms, and normalization methods, which may have contributed to heterogeneity in results. Second, many studies were limited by small sample sizes and lacked adequate external validation cohorts, reducing the generalizability of findings. Third, most circulating miRNA studies did not control for confounding factors such as comorbidities, medication use, or demographic variables, potentially biasing the observed associations. Finally, publication bias cannot be excluded, as studies with positive findings are more likely to be published. Fourth, meta-analysis of diagnostic accuracy was not feasible because of inconsistent reporting of sensitivity, specificity, thresholds, and raw contingency data.

## 6 Future prospective

To fully realize the potential of miRNAs as diagnostic and prognostic biomarkers in abdominal aortic aneurysm, future efforts should focus on several key strategies. First, standardization of pre-analytical and analytical protocols, including sample collection, RNA isolation, and quantification methods, is essential to ensure reproducibility across studies and clinical settings. Second, large-scale, multi-center validation studies are needed to confirm the specificity and sensitivity of candidate miRNAs in diverse populations. Third, integrating multi-omics approaches (e.g., combining miRNA profiles with proteomics, metabolomics, and genomic data) can improve predictive accuracy and uncover mechanistic insights. Finally, the development of point-of-care, cost-effective detection platforms, such as microfluidic chips or nanoparticle-based assays, will facilitate clinical translation.

## 7 Conclusion

This systematic review summarizes current evidence regarding tissue and circulating miRNA dysregulation in AAA. Several candidates, including miR-21, miR-146a, miR-155, miR-125b, and miR-29 family members, were recurrently reported and are biologically linked to inflammation, extracellular matrix remodeling, and vascular smooth muscle dysfunction. However, substantial methodological heterogeneity, limited external validation, and inconsistent replication currently restrict immediate clinical application. Standardized multicenter prospective studies are required before miRNA-based biomarkers can be translated into routine AAA screening, prognostication, or therapeutic monitoring.

## Availability of data and materials

All data generated or analyzed during this study are included in this published article [and its supplementary information files].

## Ethics approval and consent to participate

Not applicable.

## Clinical Trial Number in the manuscript

Not applicable.

## Consent for publication

Not applicable.

## Generative ai

In preparing this article, the authors used the Grammarly application to enhance linguistic accuracy and clarity. The manuscript underwent meticulous double-checking to ensure precision, and the authors assume full responsibility for the integrity and originality of the content presented herein.

## Funding

None.

## CRediT authorship contribution statement

**Pooya Eini:** Conceptualization, Data curation, Formal analysis, Investigation, Methodology, Project administration, Resources, Software, Supervision, Validation, Visualization, Writing – original draft, Writing – review & editing. **Peyman Eini:** Writing – original draft, Writing – review & editing. **Homa serpoush:** Data curation, Investigation, Writing – original draft. **Mohammad Rezayee:** Formal analysis, Investigation. **Jason Tremblay:** Validation, Writing – review & editing.

## Declaration of competing interest

The authors declare that they have no competing interests.

## References

[1] Sajjadi S.M., Mohebbi A., Ehsani A. (2025). Identifying abdominal aortic aneurysm size and presence using Natural Language Processing of radiology reports: a systematic review and meta-analysis. Abdom. Radiol..

[2] Leite T.F.O., da Silva E.R., Evelyn K., Tirapelli D., Joviliano E.E. (2025). Expression of plasma levels of miRNA-181b and miRNA-21 in patients with abdominal aortic aneurysms and their effect on clinical outcome after endovascular treatment. J. Endovasc. Ther..

[3] Xiao X., Li C., Huang X. (2024). Single-cell RNA sequencing reveals that NRF2 regulates vascular smooth muscle cell phenotypic switching in abdominal aortic aneurysm. FASEB J..

[4] Tasopoulou K.M., Karakasiliotis I., Argyriou C. (2024). Next-generation sequencing of microRNAs in small abdominal aortic aneurysms: MiR-24 as a biomarker. Ann. Vasc. Surg..

[5] Thanigaimani S., Iyer V., Bingley J. (2023). Association between serum MicroRNAs and abdominal aortic aneurysm diagnosis and growth. Eur. J. Vasc. Endovasc. Surg..

[6] Cai H., Huang L., Wang M. (2024). Pterostilbene alleviates abdominal aortic aneurysm via inhibiting macrophage pyroptosis by activating the miR-146a-5p/TRAF6 axis. Food Funct..

[7] Vanan A.G., Ghorbaninezhad F., Nikeghbali G. (2025). CCAT2 role in gastrointestinal cancer progression and metastasis: a novel target for therapeutic strategies. Clin. Exp. Med..

[8] Ghorbani Vanan A., Nami M.T., Ghorbaninezhad F. (2025). Macrophage polarization in hepatocellular carcinoma: a lncRNA-centric perspective on tumor progression and metastasis. Clin. Exp. Med..

[9] Page M.J., McKenzie J.E., Bossuyt P.M. (2021). The PRISMA 2020 statement: an updated guideline for reporting systematic reviews. Br. Med. J..

[10] Wells G., Shea B., O'Connell D. (2000).

[11] Maegdefessel L., Azuma J., Toh R. (2012). Inhibition of microRNA-29b reduces murine abdominal aortic aneurysm development. J. Clin. Investig..

[12] Maegdefessel L., Azuma J., Toh R. (2012). MicroRNA-21 blocks abdominal aortic aneurysm development and nicotine-augmented expansion. Sci. Transl. Med..

[13] Maegdefessel L., Spin J.M., Raaz U. (2014). miR-24 limits aortic vascular inflammation and murine abdominal aneurysm development. Nat. Commun..

[14] Biros E., Moran C.S., Wang Y., Walker P.J., Cardinal J., Golledge J. (2014). microRNA profiling in patients with abdominal aortic aneurysms: the significance of miR-155. Clin. Sci. (Lond.).

[15] Spear R., Boytard L., Blervaque R. (2015). Adventitial tertiary lymphoid organs as potential source of MicroRNA biomarkers for abdominal aortic aneurysm. Int. J. Mol. Sci..

[16] Stather P.W., Sylvius N., Sidloff D.A. (2015). Identification of microRNAs associated with abdominal aortic aneurysms and peripheral arterial disease. Br. J. Surg..

[17] Zhang W., Shang T., Huang C. (2015). Plasma microRNAs serve as potential biomarkers for abdominal aortic aneurysm. Clin. Biochem..

[18] Busch A., Busch M., Scholz C.J. (2016). Aneurysm miRNA signature differs, depending on disease localization and morphology. Int. J. Mol. Sci..

[19] Wanhainen A., Mani K., Vorkapic E. (2017). Screening of circulating microRNA biomarkers for prevalence of abdominal aortic aneurysm and aneurysm growth. Atherosclerosis.

[20] Ni H., Lv H., Qiu Y. (2016). MicroRNA-155 as a potential plasma non-invasive biomarker for the diagnosis and prognosis of abdominal aortic aneurysm. Int. J. Clin. Exp. Pathol..

[21] Venkatesh P., Phillippi J., Chukkapalli S. (2017). Aneurysm-specific miR-221 and miR-146a participates in human thoracic and abdominal aortic aneurysms. Int. J. Mol. Sci..

[22] Gao P., Si J., Yang B., Yu J. (2017). Upregulation of MicroRNA-15a contributes to pathogenesis of Abdominal Aortic Aneurysm (AAA) by modulating the expression of cyclin-dependent kinase inhibitor 2B (CDKN2B). Med. Sci. Monit..

[23] Liang B., Che J., Zhao H., Zhang Z., Shi G. (2017). MiR-195 promotes abdominal aortic aneurysm media remodeling by targeting Smad3. Cardiovasc. Ther..

[24] Courtois A., Nusgens B., Garbacki N. (2018). Circulating microRNAs signature correlates with positive [(18)F]fluorodeoxyglucose-positron emission tomography in patients with abdominal aortic aneurysm. J. Vasc. Surg..

[25] Ma X., Yao H., Yang Y. (2018). miR-195 suppresses abdominal aortic aneurysm through the TNF-alpha/NF-kappaB and VEGF/PI3K/Akt pathway. Int. J. Mol. Med..

[26] Riches K., Clark E., Helliwell R.J. (2018). Progressive development of aberrant smooth muscle cell phenotype in abdominal aortic aneurysm disease. J. Vasc. Res..

[27] Tenorio E.J.R., Braga A.F.F., Tirapelli D., Ribeiro M.S., Piccinato C.E., Joviliano E.E. (2018). Expression in whole blood samples of miRNA-191 and miRNA-455-3p in patients with AAA and their relationship to clinical outcomes after endovascular repair. Ann. Vasc. Surg..

[28] Cerna V., Ostasov P., Pitule P., Molacek J., Treska V., Pesta M. (2019). The expression profile of MicroRNAs in small and large abdominal aortic aneurysms. Cardiol. Res. Pract..

[29] Spear R., Boytard L., Blervaque R. (2019). Let-7f: a new potential circulating biomarker identified by miRNA profiling of cells isolated from human abdominal aortic aneurysm. Int. J. Mol. Sci..

[30] Araujo N.N.F., Lin-Wang H.T., Germano J.F. (2019). Dysregulation of microRNAs and target genes networks in human abdominal aortic aneurysm tissues. PLoS One.

[31] Zhao L., Huang J., Zhu Y. (2020). miR-33-5p knockdown attenuates abdominal aortic aneurysm progression via promoting target adenosine triphosphate-binding cassette transporter A1 expression and activating the PI3K/Akt signaling pathway. Perfusion.

[32] Han Z.L., Wang H.Q., Zhang T.S., He Y.X., Zhou H. (2020). Up-regulation of exosomal miR-106a may play a significant role in abdominal aortic aneurysm by inducing vascular smooth muscle cell apoptosis and targeting TIMP-2, an inhibitor of metallopeptidases that suppresses extracellular matrix degradation. Eur. Rev. Med. Pharmacol. Sci..

[33] Plana E., Galvez L., Medina P. (2020). Identification of novel microRNA profiles dysregulated in plasma and tissue of abdominal aortic aneurysm patients. Int. J. Mol. Sci..

[34] Torres-Do Rego A., Barrientos M., Ortega-Hernandez A. (2020). Identification of a plasma microrna signature as biomarker of subaneurysmal aortic dilation in patients with high cardiovascular risk. J. Clin. Med..

[35] Zhang C., Wang H., Yang B. (2020). miR-146a regulates inflammation and development in patients with abdominal aortic aneurysms by targeting CARD10. Int. Angiol..

[36] Zalewski D.P., Ruszel K.P., Stepniewski A. (2020). Dysregulation of microRNA modulatory network in abdominal aortic aneurysm. J. Clin. Med..

[37] Missae L., Rossoni B., Tenorio E.J.R., Ribeiro M.S., Tirapelli D., Joviliano E.E. (2021). Expression of MicroRNA-1281, C-Reactive protein, and renal function in individuals with abdominal aortic aneurysm and their clinical correlation after endovascular repair. Braz. J. Cardiovasc. Surg..

[38] Licholai S., Studzinska D., Plutecka H., Gubala T., Szczeklik W., Sanak M. (2021). MiR-191 as a key molecule in aneurysmal aortic remodeling. Biomolecules.

[39] Zhou F., Zheng Z., Zha Z., Xiong T., Pan Y. (2022). Nuclear paraspeckle assembly transcript 1 enhances hydrogen peroxide-induced human vascular smooth muscle cell injury by regulating miR-30d-5p/A disintegrin and metalloprotease 10. Circ. J..

[40] Wang Y., Zhai S., Xing J., He Y., Li T. (2022). LncRNA GAS5 promotes abdominal aortic aneurysm formation through regulating the miR-185-5p/ADCY7 axis. Anti Cancer Drugs.

[41] Jing J., Chang M., Jiang S. (2023). Clinical value of serum miR-1-3p as a potential circulating biomarker for abdominal aortic aneurysm. Ann. Med..

[42] Li T., Jing J., Sun L. (2023). The SNP rs4591246 in pri-miR-1-3p is associated with abdominal aortic aneurysm risk by regulating cell phenotypic transformation via the miR-1-3p/TLR4 axis. Int. Immunopharmacol..

[43] Tian Y., Li X., Bai C. (2023). lncRNA MIR503HG targets miR-191-5p/PLCD1 axis and negatively modulates apoptosis, extracellular matrix disruption, and inflammation in abdominal aortic aneurysm. Mediat. Inflamm..

[44] Winski G., Chernogubova E., Busch A. (2025). MicroRNA-15a-5p mediates abdominal aortic aneurysm progression and serves as a potential diagnostic and prognostic circulating biomarker. Commun. Med. (Lond).

