# Supplementary material for: MicroRNA expression profiles in abdominal aortic aneurysms: A systematic review of potential diagnostic and prognostic biomarkers
Source: Int J Cardiol Cardiovasc Risk Prev. 2026 May 7;30:200651. doi: 10.1016/j.ijcrp.2026.200651 (PMC13194167; doi:10.1016/j.ijcrp.2026.200651)
Supplement: Multimedia component 1 [file mmc1.docx]

| Supplementary table 1. Search syntax for different databases. | | |
| --- | --- | --- |
| database | Search syntax | results |
| PUBMED | ("Abdominal Aortic Aneurysms"[tiab] OR "Abdominal Aortic Aneurysm"[tiab] OR "Abdominal Aorta Aneurysm"[tiab] OR "Abdominal Aorta Aneurysms"[tiab]) AND ("Micro RNA"[tiab] OR MicroRNA[tiab] OR miRNA[tiab] OR miRNAs[tiab] OR "Small Temporal RNA"[tiab] OR stRNA[tiab] OR "Primary MicroRNA"[tiab] OR "pri-miRNA"[tiab] OR "Primary miRNA"[tiab] OR "pre-miRNA"[tiab] OR "Untranslated RNA"[tiab] OR "Noncoding RNA"[tiab] OR npcRNA[tiab] OR "Non-Coding RNA"[tiab] OR "RNA, Non Coding"[tiab] OR "Non-Protein-Coding RNA"[tiab] OR "RNA, Noncoding"[tiab] OR "RNA, Nontranslated"[tiab] OR "Nontranslated RNA"[tiab]) | 187 |
| SCOPUS | ( TITLE-ABS ( "Abdominal Aortic Aneurysms" OR "Abdominal Aortic Aneurysm" OR "Abdominal Aorta Aneurysm" OR "Abdominal Aorta Aneurysms" ) AND TITLE-ABS ( "Micro RNA" OR MicroRNA OR miRNA OR miRNAs OR "Small Temporal RNA" OR stRNA OR "Primary MicroRNA" OR "pri-miRNA" OR "Primary miRNA" OR "pre-miRNA" OR "Untranslated RNA" OR "Noncoding RNA" OR npcRNA OR "Non-Coding RNA" OR "RNA, Non Coding" OR "Non-Protein-Coding RNA" OR "RNA, Noncoding" OR "RNA, Nontranslated" OR "Nontranslated RNA" ) ) | 199 |
| WEB OF SCIENCE | TS=("Abdominal Aortic Aneurysms" OR "Abdominal Aortic Aneurysm" OR "Abdominal Aorta Aneurysm" OR "Abdominal Aorta Aneurysms")  AND  TS=("Micro RNA" OR MicroRNA OR miRNA OR miRNAs OR "Small Temporal RNA" OR stRNA OR "Primary MicroRNA" OR "pri-miRNA" OR "Primary miRNA" OR "pre-miRNA" OR "Untranslated RNA" OR "Noncoding RNA" OR npcRNA OR "Non-Coding RNA" OR "RNA, Non Coding" OR "Non-Protein-Coding RNA" OR "RNA, Noncoding" OR "RNA, Nontranslated" OR "Nontranslated RNA") | 449 |
| EMBASE | 'abdominal aortic aneurysm'/de AND ('microrna'/de OR 'untranslated rna'/de) | 309 |
| EBSCO | (TI ("Abdominal Aortic Aneurysms" OR "Abdominal Aortic Aneurysm" OR "Abdominal Aorta Aneurysm" OR "Abdominal Aorta Aneurysms") OR AB ("Abdominal Aortic Aneurysms" OR "Abdominal Aortic Aneurysm" OR "Abdominal Aorta Aneurysm" OR "Abdominal Aorta Aneurysms")) AND (TI ("Micro RNA" OR MicroRNA OR miRNA OR miRNAs OR "Small Temporal RNA" OR stRNA OR "Primary MicroRNA" OR "pri-miRNA" OR "Primary miRNA" OR "pre-miRNA") OR AB ("Micro RNA" OR MicroRNA OR miRNA OR miRNAs OR "Small Temporal RNA" OR stRNA OR "Primary MicroRNA" OR "pri-miRNA" OR "Primary miRNA" OR "pre-miRNA" OR "Untranslated RNA" OR "Noncoding RNA" OR npcRNA OR "Non-Coding RNA" OR "RNA, Non Coding" OR "Non-Protein-Coding RNA" OR "RNA, Noncoding" OR "RNA, Nontranslated" OR "Nontranslated RNA") OR AB ("Untranslated RNA" OR "Noncoding RNA" OR npcRNA OR "Non-Coding RNA" OR "RNA, Non Coding" OR "Non-Protein-Coding RNA" OR "RNA, Noncoding" OR "RNA, Nontranslated" OR "Nontranslated RNA")) | 127 |
| GOOGLE SCHOLAR | "abdominal aortic aneurysm" AND ("microrna" OR "untranslated rna") | 100 |
| TOTAL |  | 1338 |

| Supplementary table 2. Study characteristics of reviewed studies. | | | | | | | | | | | | | | | |
| --- | --- | --- | --- | --- | --- | --- | --- | --- | --- | --- | --- | --- | --- | --- | --- |
| **Study** | **miRNA** | **P Value** | **FDR** | **Direction** | **Case Definition** | **Number of Cases** | **Control Definition** | **Number of Controls** | **Sample Tissue** | **Screening Method** | **Validation Method** | **Normalization Control** | **Differential Expression Definition** | **Pathway** | **Keynote** |
| Torres-Do Rego_2020 | let-7b-5p | 0.0042 | . | -1 | AAA (aortic diameter ≥30 mm) | 15 | Normal aortic diameter (<25 mm) | 32 | Plasma | qPCR array (miR CURYLNA Human panel I+II) | qRT-PCR | miR-451a | p<0.0042, fold change ≥2 | Not specified | Downregulated in SAD and AAA plasma, previously associated with aorta aneurysms |
| Thanigaimani_2032 | let-7b-5p | 0.05 | 0.05 | 1 | AAA patients with infrarenal aortic diameter ≥30 mm | 108 | Healthy controls and PAD patients | 24 | Serum | NanoString Human miRNA v3 assay | RT-qPCR | hsa-miR-24-3p and hsa-miR-484 | p<0.05, q<0.05 | Morphogenesis and cellular response to endogenous stimuli | Upregulated in AAA serum, positively correlated with aortic diameter, associated with AAA diagnosis (OR 13.06), improves diagnostic AUC with miR-548n to 98.0% |
| Licholai_2016 | miR-1 | 0.05 | . | 1 | TAA diameter >5.5 cm by echocardiography | 15 | Non-aneurysmal aortic arch from same patient | 15 | Aortic tissue | TaqMan OpenArray | qRT-PCR (LNA PCR) | miR-193b/miR-125b | p<0.05, fold change >2 | ECM receptor interaction | Upregulated, linked to ECM remodeling |
| Venkatesh_2017 | miR-1 | 0.05 | . | -1 | TAA diameter >5.5 cm by imaging | 7 | Non-aneurysmal aorta <45 mm | 8 | Aortic tissue | None | qRT-PCR | RNU44 | p<0.05 | Not specified | Downregulated in TAA, no change in AAA |
| Plana_2020 | miR-1 | 0.001 | . | -1 | AAA diagnosed by CT (>5.5 cm) | 21 | Organ donors (no AAA) | 8 | Aortic tissue | None | qRT-PCR | miR-423-5p | p<0.05, fold change >2 or <-2 | Not specified | Downregulated in AAA tissue (4.8-fold, p<0.001) |
| Jing_2023 | miR-1-3p | 0.05 | . | -1 | AAA patients diagnosed by CTA | 200 | Healthy individuals without AAA, frequency-matched by gender and age | 200 | Serum | Bioinformatic prediction (miRTarBase | TargetScan) | qRT-PCR, U6 | p<0.05 | Not applicable | Downregulated in AAA serum, negatively correlated with WBC, CRP, and AAA image parameters (diameter, area, volume), low levels (≤0.62) associated with increased AAA risk, involved in negative regulation of apoptosis, angiogenesis, and cell proliferation |
| Li_2023 | miR-1-3p | 0.05 | . | -1 | AAA patients diagnosed by CTA | 335 | Healthy controls without AAA | 335 | Aortic tissue and HASMCs | Bioinformatic prediction (RNAfold ) | qRT-PCR | dual-luciferase assay Western blot, ELISA, immunofluorescence, U6 and β-actin | p<0.05 | miR-1-3p/TLR4 axis | Downregulated in AAA tissues (0.36 ± 0.41 vs. 1.12 ± 0.59, p=0.003) and Ang II-induced HASMCs, negatively correlated with TLR4 expression (r=-0.618, p=0.014) and AAA diameter (r=-0.563, p=0.029), rs4591246 AA genotype reduces miR-1-3p expression, promotes TLR4 expression and HASMC phenotypic switching |
| Spear_2019 | miR-1207-5p | 0.05 | . | 0 | AAA diagnosed by imaging or surgery | 6 | Non-aneurysmal aorta from organ donors | 6 | SMCs and macrophages (LCM) | Microarray | qRT-PCR | RNU6-2 | p<0.05 | Not specified | Downregulated in aneurysmal SMCs (0.6 fold); upregulated in M1 (18 fold) and M2 (1.1 fold) |
| Araujo_2019 | miR-1207-5p | 0.05 | . | 0 | AAA diagnosed by imaging or surgery | 18 | Non-aneurysmal aorta from organ donors | 6 | Aortic tissue | qPCR array | qRT-PCR | SNORD61/SNORD68/RNU6-6p | p<0.05, fold change ≥2 | Not specified | Upregulated in qPCR array but not validated by qRT-PCR |
| Venkatesh_2017 | miR-125b-5p | 0.05 | . | -1 | TAA diameter >5.5 cm by imaging | 5 | Non-aneurysmal aorta <45 mm | 3 | Aortic tissue | Microarray | None | RNU44 | p<0.05, fold change >1.5 | Not specified | Downregulated in TAA |
| Courtois_2017 | miR-125b-5p | 0.05 | . | -1 | AAA with positive FDG uptake (A+) | 22 | AAA without FDG uptake (A0) | 35 | Plasma | miRNA PCR array | qRT-PCR | cel-miR-39 | p<0.05, fold change >1.5 | MAPK signaling | Downregulated in A+ plasma, downregulated in A+pos media, targets MMP13 |
| Courtois_2017 | miR-125b-5p | 0.05 | . | -1 | AAA with positive FDG uptake (A+pos media) | 9 | AAA without FDG uptake (A0) or A+neg media | 12 | Aortic tissue | None | qRT-PCR | miR-16/SNORD95 | p<0.05 | MAPK signaling | Downregulated in A+pos media, targets MMP13 |
| Araujo_2019 | miR-125b-5p | 0.05 | . | -1 | AAA diagnosed by imaging or surgery | 18 | Non-aneurysmal aorta from organ donors | 6 | Aortic tissue | qPCR array | qRT-PCR | SNORD61/SNORD68/RNU6-6p | p<0.05, fold change ≥2 | Eicosanoid synthesis, metalloprotease/TIMP | Downregulated in AAA, paired interaction with ALOX5 |
| Venkatesh_2017 | miR-126 | 0.05 | . | 1 | TAA/AAA diameter >5.5 cm or >50% normal | 10 | Non-aneurysmal aorta <45 mm | 8 | Aortic tissue | Microarray | qRT-PCR | RNU44 | p<0.05 | Angiogenesis | Upregulated in TAA and AAA |
| Gan_2018 | miR-126 | 0.05 | 0.05 | 1 | AAA diagnosed by imaging or surgery | 8 | Non-aneurysmal aorta from organ donors | 2 | Aortic tissue | Microarray | None | Not specified | \|logFC\|>1, FDR<0.05 | Ubiquitin-mediated proteolysis | Upregulated in AAA, regulates hub gene CDC16 |
| Venkatesh_2017 | miR-1260a | 0.05 | . | 1 | TAA diameter >5.5 cm by imaging | 5 | Non-aneurysmal aorta <45 mm | 3 | Aortic tissue | Microarray | None | RNU44 | p<0.05, fold change >1.5 | Not specified | Upregulated in TAA |
| Plana_2020 | miR-1260a | 0.05 | . | 1 | AAA diagnosed by CT (>5.5 cm) | 30 | Healthy volunteers (ultrasound-confirmed no AAA) | 16 | Plasma | qPCR array | qRT-PCR | miR-191-5p | p<0.05, fold change 1.49-2 | Not specified | Overexpressed in AAA plasma (fold change 1.49-2), poor expression in tissue |
| Zhang_2015 | miR-1281 | 0.001 | . | 1 | AAA diameter >50% normal by CT | 60 | Healthy, no AAA by ultrasound | 60 | Plasma | Microarray | qRT-PCR | cel-miR-39 | p<0.05, fold change >5 | Not specified | Upregulated, AUC 0.9206, potential biomarker |
| Missae_2020 | miR-1281 | 0.0001 | . | 1 | Degenerative infrarenal AAA eligible for endovascular repair | 30 | Pre-treatment AAA patients (baseline) | 30 | Peripheral venous blood | qRT-PCR | qRT-PCR | U6 | p<0.05 | Not specified | Upregulated before endovascular treatment (median 1.66-fold, range 0.21-8.14), significantly reduced post-treatment (median 0.27, p<0.0001), no correlation with total cholesterol (p=0.22) or triglycerides (p=0.8) |
| Licholai_2016 | miR-133a | 0.05 | . | 1 | TAA diameter >5.5 cm by echocardiography | 15 | Non-aneurysmal aortic arch from same patient | 15 | Aortic tissue | TaqMan OpenArray | qRT-PCR (LNA PCR) | miR-193b/miR-125b | p<0.05, fold change >2 | Apoptosis | Upregulated, linked to cell death pathways |
| Venkatesh_2017 | miR-133a | 0.05 | . | -1 | TAA diameter >5.5 cm by imaging | 7 | Non-aneurysmal aorta <45 mm | 8 | Aortic tissue | None | qRT-PCR | RNU44 | p<0.05 | Not specified | Downregulated in TAA, no change in AAA |
| Plana_2020 | miR-133a | 0.001 | . | -1 | AAA diagnosed by CT (>5.5 cm) | 21 | Organ donors (no AAA) | 8 | Aortic tissue | None | qRT-PCR | miR-423-5p | p<0.05, fold change >2 or <-2 | Not specified | Downregulated in AAA tissue (4.4-fold, p<0.001) |
| Plana_2020 | miR-133b | 0.001 | . | -1 | AAA diagnosed by CT (>5.5 cm) | 21 | Organ donors (no AAA) | 8 | Aortic tissue | None | qRT-PCR | miR-423-5p | p<0.05, fold change >2 or <-2 | Not specified | Downregulated in AAA tissue (4.6-fold, p<0.001) |
| Torres-Do Rego_2020 | miR-133b | 0.0042 | . | -1 | AAA (aortic diameter ≥30 mm) | 15 | Normal aortic diameter (<25 mm) | 32 | Plasma | qPCR array (miR CURYLNA Human panel I+II) | qRT-PCR | miR-451a | p<0.0042, fold change ≥2 | Not specified | Downregulated in SAD and AAA plasma, previously associated with aorta aneurysms |
| Courtois_2017 | miR-142-5p | 0.05 | . | 1 | AAA with positive FDG uptake (A+) | 22 | AAA without FDG uptake (A0) | 35 | Plasma | miRNA PCR array | qRT-PCR | cel-miR-39 | p<0.05, fold change >1.5 | TGF-β signaling | Upregulated in A+ plasma |
| Si_2021 | miR-142-5p | 0.01 | . | Not specified | AAA tissue samples | 97 | Normal artery tissue from brain-dead patients | 23 | Aortic tissue | Microarray (GSE47472, GSE52093, GSE57691, GSE98278 , GSE144431) | TargetScan and Circinteractome databases | Not specified | p<0.01, \|log2FC\|>2 | circRNA-miRNA-mRNA network | Identified as common miRNA interacting with hub genes (IL6, RPS27A, JUN, UBC, UBA52, FOS, IL1B, MMP9, SPP1, CCL2) and circRNAs, involved in AAA-related ceRNA network |
| Cao_2017 | miR-144-3p | 0.05 | 0.01 | 1 | AAA diagnosed by imaging or surgery | 60 | Non-aneurysmal aortic tissue | 60 | Aortic tissue | Microarray | qRT-PCR | U6 | p<0.05, fold change >2 | Not specified | Upregulated in AAA |
| Plana_2020 | miR-144-3p | 0.001 | . | 1 | AAA diagnosed by CT (>5.5 cm) | 30 | Healthy volunteers (ultrasound-confirmed no AAA) | 16 | Plasma and aortic tissue | qPCR array | qRT-PCR | miR-191-5p (plasma); miR-423-5p (tissue) | p<0.05, fold change >2 or <-2 | Not specified | Upregulated in AAA tissue (7.2-fold, p<0.001) and plasma (fold change 1.49-2) |
| Licholai_2016 | miR-145 | 0.05 | . | 1 | TAA diameter >5.5 cm by echocardiography | 15 | Non-aneurysmal aortic arch from same patient | 15 | Aortic tissue | TaqMan OpenArray | qRT-PCR (LNA PCR) | miR-193b/miR-125b | p<0.05, fold change >2 | VEGF signaling | Upregulated, linked to angiogenesis |
| Venkatesh_2017 | miR-145 | 0.01 | . | -1 | AAA diameter >50% normal | 3 | Non-aneurysmal aorta <45 mm | 8 | Aortic tissue | None | qRT-PCR | RNU44 | p<0.01 | VEGF signaling | Downregulated in AAA |
| Riches_2018 | miR-145 | 0.05 | . | 1 | AAA diagnosed by imaging or surgery | 31 | Non-aneurysmal saphenous vein or internal mammary artery | 57 | Cultured SMCs | None | qRT-PCR | U6 | p<0.05, fold change >2 | Not specified | Upregulated in AAA-SMC compared to non-aneurysmal SMC |
| Xiao_2024 | miR-145 | 0.05 | . | 1 | Patients with AAA undergoing surgical repair and Ang II-induced AAA in NRF2ΔVSMC mice | 3 | Healthy human aortic tissue from donors and control mice | 3 | Aortic tissue and VSMCs | scRNA-seq (GSE166676,GSE152583) | qRT-PCR | Western blotting, Immunohistochemistry, β-actin | p<0.05 | NRF2/miR-145/MYOCD axis | Upregulated in AAA group and NRF2-positive VSMCs, positively correlated with NRF2, promotes expression of contractile biomarker genes (α-SMA, CNN1, SM22α) via MYOCD, NRF2 silencing with miR-145 inhibition decreases these genes, while miR-145 overexpression upregulates them |
| Venkatesh_2017 | miR-146a | 0.05 | . | 1 | AAA diameter >50% normal | 3 | Non-aneurysmal aorta <45 mm | 8 | Aortic tissue | None | qRT-PCR | RNU44 | p<0.05 | Inflammation | Upregulated in AAA |
| Zhang_2020 | miR-146a | 0.001 | . | 1 | AAA (clinical diagnosis) | 62 | Healthy individuals (medical examination) | 42 | Serum | PBMCs and aortic tissue | RT-qPCR | U6 | p<0.05 | NF-kB pathway via CARD10, SIRT1, p65, MMP-2, MMP-9 | Upregulated in AAA serum (p<0.001), PBMCs (p<0.001), and aortic tissue (p=0.037); negatively correlated with TNF-α (r=-0.584, p<0.001), IFN-γ (r=-0.537, p<0.001), CRP (r=-0.549, p<0.001), CARD10 mRNA (r=-0.477, p<0.001 in PBMCs; r=-0.372, p<0.001 in tissue), MMP-9 mRNA (r=-0.529, p=0.02), MMP-2 mRNA (r=-0.478, p=0.0383); positively correlated with IL-10 (r=0.403, p=0.0012); inhibits NF-kB pathway and MMP-2/MMP-9 expression; reduces TNF-α-induced HUVEC apoptosis |
| Plana_2020 | miR-146a-5p | 0.001 | . | 1 | AAA diagnosed by CT (>5.5 cm) | 30 | Healthy volunteers (ultrasound-confirmed no AAA) | 16 | Plasma and aortic tissue | qPCR array | qRT-PCR | miR-191-5p (plasma); miR-423-5p (tissue) | p<0.05, fold change >2 or <-2 | Not specified | Upregulated in AAA tissue (5.8-fold, p<0.001) and plasma (fold change 1.49-2) |
| Cai_2024 | miR-146a-5p | 0.01 | . | 1 | AAA patients undergoing open repair and PPE/Ang II-induced AAA mouse models | 10 | Normal aortas from organ donors and control mice | 8 | Aortic tissue | plasma and macrophages, mRNA-seq (GSE221735), bioinformatic prediction (PITA, microT, TargetScan, miRm) | qRT-PCR | Western blot, ELISA, luciferase assay, YO-PRO-1 staining, Calcein-AM/PI staining, GAPDH, U6 | p<0.05 | miR-146a-5p/TRAF6 axis | Upregulated in AAA aortas and plasma, PTE treatment enhances expression, inhibits macrophage pyroptosis and AAA formation by targeting TRAF6, miR-146a-5p knockout reverses PTE effects |
| Araujo_2019 | miR-150-5p | 0.05 | . | 1 | AAA diagnosed by imaging or surgery | 18 | Non-aneurysmal aorta from organ donors | 6 | Aortic tissue | qPCR array | qRT-PCR | SNORD61/SNORD68/RNU6-6p | p<0.05, fold change ≥2 | Eicosanoid synthesis, metalloprotease/TIMP | Upregulated in AAA, paired interaction with CX3CL1 |
| Torres-Do Rego_2020 | miR-150-5p | 0.0042 | . | -1 | AAA (aortic diameter ≥30 mm) | 15 | Normal aortic diameter (<25 mm) | 32 | Plasma | qPCR array (miR CURYLNA Human panel I+II) | qRT-PCR | miR-451a | p<0.0042, fold change ≥2 | Not specified | Downregulated in SAD and AAA plasma, negatively correlated with age (r=-0.353, p=0.005), related to AAA molecular mechanisms |
| Biros_2014 | miR-155 | 0.031 | . | 1 | AAA diameter ≥50 mm by imaging | 6 | Non-dilated AAA neck, diameter <30 mm | 6 | Aortic tissue | Luminex FlexmiR | qRT-PCR | U6 | p<0.05 | Inflammation | Validated by qPCR, linked to T-cell infiltration |
| Biros_2014 | miR-155 | 0.554 | 0.554 | 1 | AAA diameter ≥50 mm by imaging | 10 | No AAA, diameter <30 mm, peripheral artery disease | 10 | Serum | Luminex FlexmiR | None | snoRNA | >2-fold, FDR<0.5 | Inflammation | Borderline significant in serum |
| Licholai_2016 | miR-155 | 0.05 | . | 1 | TAA diameter >5.5 cm by echocardiography | 15 | Non-aneurysmal aortic arch from same patient | 15 | Aortic tissue | TaqMan OpenArray | qRT-PCR (LNA PCR) | miR-193b/miR-125b | p<0.05, fold change >2 | TGF-β signaling | Upregulated, linked to inflammatory signaling |
| Ni_2016 | miR-155 | 0.05 | . | 1 | AAA diameter >50% normal by Doppler Ultrasound/CT/angiography | 116 | No AAA or severe chronic diseases | 130 | Red blood cells | None | qRT-PCR | U6 | p<0.05 | Inflammation | Upregulated, correlated with age and tumor size, linked to prognosis |
| Venkatesh_2017 | miR-155 | 0.05 | . | -1 | TAA/AAA diameter >5.5 cm or >50% normal | 10 | Non-aneurysmal aorta <45 mm | 8 | Aortic tissue | None | qRT-PCR | RNU44 | p<0.05 | Inflammation | Downregulated in TAA and AAA |
| Spear_2015 | miR-15a-3p | 0.05 | . | -1 | AAA diameter >50 mm or growth >10 mm/6 months by ultrasound | 20 | Non-aneurysmal aorta from organ donors | 14 | Aortic tissue | Microarray | qRT-PCR | RNU6-2 | p<0.05 | Angiogenesis | Downregulated in ATLOs, regulates VEGF |
| Spear_2015 | miR-15a-3p | 0.05 | . | -1 | AAA diameter >50 mm or growth >10 mm/6 months by ultrasound | 24 | PAD, no AAA by ultrasound | 18 | Plasma | Microarray | qRT-PCR | cel-miR-39 | p<0.05 | Angiogenesis | Potential biomarker in plasma |
| Venkatesh_2017 | miR-16-5p | 0.05 | . | 1 | TAA diameter >5.5 cm by imaging | 5 | Non-aneurysmal aorta <45 mm | 3 | Aortic tissue | Microarray | None | RNU44 | p<0.05, fold change >1.5 | Not specified | Upregulated in TAA |
| Araujo_2019 | miR-16-5p | 0.05 | . | 0 | AAA diagnosed by imaging or surgery | 18 | Non-aneurysmal aorta from organ donors | 6 | Aortic tissue | qPCR array | qRT-PCR | SNORD61/SNORD68/RNU6-6p | p<0.05, fold change ≥2 | Not specified | Upregulated in qPCR array but not validated by qRT-PCR |
| Venkatesh_2017 | miR-185-5p | 0.05 | . | 1 | TAA diameter >5.5 cm by imaging | 5 | Non-aneurysmal aorta <45 mm | 3 | Aortic tissue | Microarray | None | RNU44 | p<0.05, fold change >1.5 | Not specified | Upregulated in TAA |
| Wang_2022 | miR-185-5p | 0.05 | . | -1 | AAA patients undergoing surgical resection | 40 | Adjacent normal aortic tissues | 40 | Aortic tissue and HASMCs | Bioinformatic prediction (StarBase) | qRT-PCR | dual-luciferase assay and Western blot U6 and GAPDH | p<0.05 | GAS5/miR-185-5p/ADCY7 axis | Downregulated in AAA tissues and ANGII-induced HASMCs, promotes proliferation and inhibits apoptosis and inflammatory response in HASMCs, targets ADCY7, sponged by lncRNA GAS5, negatively correlated with GAS5 and ADCY7 expression, regulates AKT signaling pathway |
| Tenorio_2018 | miR-191 | 0.0001 | . | -1 | Infrarenal AAA eligible for endovascular repair | 30 | Preoperative AAA patients (same patients, post-repair) | 30 | Whole blood | None | qRT-PCR | U6 | p<0.0001 | MMPs, IL-6, TNF-α | Downregulated post-repair, interacts with SATB1, CDK6, VCAM1, ICAM1, TIMP1, MMPs, IL-6, TNF-α, DAB2IP, SERPINA1, LDLR, IL-16 |
| Lichołai_2021 | miR-191 | 0.05 | . | 1 | AAA patients undergoing surgical stent graft implantation | 205 | Patients with peripheral arterial occlusive disease | 180 | Serum | RT-qPCR | RT-qPCR using TaqMan probes | miR-16 and dme-miR-7 | p<0.05, fold change >2 or <0.5 | Cell adhesion, metal-binding metallothioneins, immune activation, interleukin signaling, DNA methylation, extracellular matrix metabolism | Upregulated in AAA patient serum, induces significant changes in 1492 protein-coding genes in endothelial cells, 17 downregulated genes identified as direct targets, affects multiple biological pathways |
| Liang_2017 | miR-195 | 0.01 | . | 1 | AAA diagnosed by imaging or surgery | 15 | Non-aneurysmal aorta | 15 | Aortic tissue | None | qRT-PCR | U6 | p<0.01 | TGF-β signaling | Upregulated, negatively regulates Smad3, increases OPN/collagen III, inhibits VSMC proliferation, promotes apoptosis |
| Ma_2018 | miR-195 | 0.05 | . | 1 | AAA diagnosed by imaging or surgery | 6 | Non-aneurysmal aorta | 6 | Serum | None | qRT-PCR | U6 | p<0.05 | PI3K/Akt signaling | Upregulated in AAA serum, promotes IL-1β, IL-6, MMP-2, MMP-9, TNF-α, NF-κB; suppresses VEGF, PI3K, p-Akt |
| Goliopoulou_2023 | miR-195 | 0.05 | . | 1 | Patients with acute Stanford type A aortic dissection | 11 | Patients with normal thoracic aortic diameter undergoing CABG or valve replacement | 18 | Aortic tissue | Not applicable | qRT-PCR | U6 | p<0.05 | Not specified | Upregulated in aortic tissue of dissection group (2.72-fold, p<0.001), downregulated in aneurysm group (2.00-fold, p=0.08), not detected in plasma |
| Stather_2015 | miR-196b | 0.001 | . | -1 | AAA diameter >54 mm | 80 | Healthy, no AAA by ultrasound | 40 | Whole blood | qPCR (TaqMan Cards) | Digital PCR (OpenArray) | RNU48/MammU6 | FC>1.5, p<0.1 | Inflammation | Significant in AAA and PAD |
| Stather_2015 | miR-196b | 0.029 | . | -1 | AAA diameter >54 mm or 30-54 mm | 72 | Healthy, no AAA by ultrasound | 28 | Plasma | qPCR (TaqMan Cards) | Digital PCR (OpenArray) | RNU48/MammU6 | FC>1.5, p<0.1 | Inflammation | Downregulated in plasma, not significant after regression |
| Venkatesh_2017 | miR-204 | 0.01 | . | -1 | TAA/AAA diameter >5.5 cm or >50% normal | 10 | Non-aneurysmal aorta <45 mm | 8 | Aortic tissue | None | qRT-PCR | RNU44 | p<0.01 | Not specified | Downregulated in TAA and AAA |
| Han_2020 | miR-204 | 0.01 | . | -1 | AAA diagnosed by CTA (>5.5 cm) | 21 | Adjacent non-aneurysmal aortic tissue | 21 | Exosomes from aortic tissue and plasma | None | qRT-PCR | U6 | p<0.05 | Not specified | Downregulated in AAA plasma (p<0.01) and tissue exosomes (p<0.01) |
| Courtois_2017 | miR-204-5p | 0.05 | . | -1 | AAA with positive FDG uptake (A+) | 22 | AAA without FDG uptake (A0) | 35 | Plasma | miRNA PCR array | qRT-PCR | cel-miR-39 | p<0.05, fold change >1.5 | Not specified | Downregulated in A+ plasma, downregulated in A+pos media, targets RUNX2, HAPLN1 |
| Courtois_2017 | miR-204-5p | 0.05 | . | -1 | AAA with positive FDG uptake (A+pos media) | 9 | AAA without FDG uptake (A0) or A+neg media | 12 | Aortic tissue | None | qRT-PCR | miR-16/SNORD95 | p<0.05 | Not specified | Downregulated in A+pos media, targets RUNX2, HAPLN1 |
| Spear_2019 | miR-21 | 0.05 | . | 1 | AAA diagnosed by imaging or surgery | 6 | Non-aneurysmal aorta from organ donors | 6 | SMCs and M2 macrophages (LCM) | Microarray | qRT-PCR | RNU6-2 | p<0.05 | Not specified | Upregulated in aneurysmal SMCs (2.15 fold), M1 (5.7 fold), and M2 (8.9 fold) |
| Leite_2025 | miR-21 | 0.05 | . | 1 | Patients with infrarenal AAA eligible for endovascular repair | 47 | Volunteers without AAA or risk factors | 10 | Whole blood | Not specified | RQ-PCR (TaqMan Assay-on-demand) | U6 | p<0.05 | Not specified | Elevated 2.76-fold in AAA patients preoperatively compared to controls, reduced expression 6 months post-endovascular repair, no significant change in expression in patients with endoleaks |
| Busch_2016 | miR-21-3p | 0.05 | . | -1 | AAA diagnosed by imaging or surgery | 19 | Atherosclerotic non-aneurysmal aorta | 11 | Aortic tissue | TaqMan Array | qRT-PCR | U6/RNU48 | p<0.05 | Inflammation | Downregulated, inflammation-dependent |
| Venkatesh_2017 | miR-21-3p | 0.05 | . | 1 | TAA diameter >5.5 cm by imaging | 5 | Non-aneurysmal aorta <45 mm | 3 | Aortic tissue | Microarray | None | RNU44 | p<0.05, fold change >1.5 | Inflammation | Upregulated in TAA |
| Licholai_2016 | miR-21-5p | 0.05 | . | 1 | TAA diameter >5.5 cm by echocardiography | 15 | Non-aneurysmal aortic arch from same patient | 15 | Aortic tissue | TaqMan OpenArray | qRT-PCR (LNA PCR) | miR-193b/miR-125b | p<0.05, fold change >2 | VEGF/TGF-β/Akt-PI3K | Upregulated, regulates NOS3/PTEN |
| Venkatesh_2017 | miR-21-5p | 0.05 | . | 1 | TAA diameter >5.5 cm by imaging | 5 | Non-aneurysmal aorta <45 mm | 3 | Aortic tissue | Microarray | None | RNU44 | p<0.05, fold change >1.5 | Inflammation | Upregulated in TAA |
| Plana_2020 | miR-21-5p | 0.012 | . | 1 | AAA diagnosed by CT (>5.5 cm) | 21 | Organ donors (no AAA) | 8 | Aortic tissue | None | qRT-PCR | miR-423-5p | p<0.05, fold change >2 or <-2 | Not specified | Upregulated in AAA tissue (1.9-fold, p=0.012) |
| Spear_2019 | miR-24 | 0.01 | . | -1 | AAA diagnosed by imaging or surgery | 6 | Non-aneurysmal aorta from organ donors | 6 | SMCs and macrophages (LCM) | Microarray | qRT-PCR | RNU6-2 | p<0.05 | Not specified | Upregulated in aneurysmal SMCs (1.6 fold), M1 (2.6 fold), and M2 (4.1 fold); downregulated in whole AAA tissue (0.3 fold, p=0.01) |
| Han_2020 | miR-24 | 0.05 | . | -1 | AAA diagnosed by CTA (>5.5 cm) | 21 | Adjacent non-aneurysmal aortic tissue | 21 | Exosomes from aortic tissue and plasma | None | qRT-PCR | U6 | p<0.05 | Not specified | Downregulated in AAA plasma (p<0.05) and tissue exosomes (p<0.01) |
| Tasopoulou_2024 | miR-24 | 0.015 | . | -1 | Patients with small AAAs (dmax 3-5.4 cm) | 39 | Volunteers with chronic venous disease (CEAP stages 0-2) | 13 | Plasma | Next-Generation Sequencing (NGS) | Real-time PCR | Not specified | p<0.05 | Not specified | Downregulated in small AAAs (fold-change 0.27, p=0.015) and large AAAs (fold-change 0.15, p=0.005) compared to controls, no significant difference between small and large AAAs (fold-change 1.78, p=0.42) |
| Tasopoulou_2024 | miR-24 | 0.005 | . | -1 | Patients with large AAAs (dmax ≥5.5 cm) | 10 | Volunteers with chronic venous disease (CEAP stages 0-2) | 13 | Plasma | Next-Generation Sequencing (NGS) | Real-time PCR | Not specified | p<0.05 | Not specified | Downregulated in small AAAs (fold-change 0.27, p=0.015) and large AAAs (fold-change 0.15, p=0.005) compared to controls, no significant difference between small and large AAAs (fold-change 1.78, p=0.42) |
| Peng_2018 | miR-26a | 0.05 | 0.05 | -1 | AAA diagnosed by imaging or surgery | 30 | Non-vascular disease donors | 30 | Peripheral blood | Microarray | qRT-PCR | U6 | p<0.05 | Akt/mTOR signaling | Downregulated in AAA, protects VSMCs from H2O2-induced injury by targeting PTEN |
| Peng_2018 | miR-26a | 0.05 | 0.05 | -1 | AAA diagnosed by imaging or surgery | 30 | Non-vascular disease donors | 30 | Peripheral blood | Microarray | qRT-PCR | U6 | p<0.05 | Akt/mTOR signaling | Downregulated in AAA, protects VSMCs from H2O2-induced injury by targeting PTEN |
| Cerna_2019 | miR-27a-3p | 0.05 | 0.05 | -1 | Large AAA (>5 cm) | 6 | Non-aneurysmal aorta from cadaveric donors | 5 | Aortic tissue | Microarray | None | Not specified | FDR<0.05, fold change >2 | Not specified | Downregulated in large AAA |
| Torres-Do Rego_2020 | miR-27a-3p | 0.0042 | . | -1 | AAA (aortic diameter ≥30 mm) | 15 | Normal aortic diameter (<25 mm) | 32 | Plasma | qPCR array (miR CURYLNA Human panel I+II) | qRT-PCR | miR-451a | p<0.0042, fold change ≥2 | Not specified | Downregulated in SAD and AAA plasma, previously associated with aorta aneurysms |
| Han_2020 | miR-29 | 0.05 | . | 1 | AAA diagnosed by CTA (>5.5 cm) | 21 | Adjacent non-aneurysmal aortic tissue | 21 | Exosomes from aortic tissue and plasma | None | qRT-PCR | U6 | p<0.05 | Not specified | Upregulated in AAA plasma and tissue exosomes |
| Goliopoulou_2023 | miR-29 | 0.05 | . | 1 | Patients with acute Stanford type A aortic dissection or ascending aorta aneurysm | 32 | Patients with normal thoracic aortic diameter undergoing CABG or valve replacement | 18 | Aortic tissue | Not applicable | qRT-PCR | U6 | p<0.05 | Not specified | Upregulated in aortic tissue of dissection (2.11-fold, p=0.001) and aneurysm (2.99-fold, p<0.001) groups, not detected in plasma |
| Maegdefessel_2012 | miR-29a | 0.05 | . | 0 | AAA diagnosed by imaging or surgery | 15 | Non-aneurysmal aorta from organ donors | 5 | Infrarenal aortic tissue | None | qRT-PCR | RNU44 | p>0.05 | Collagen and ECM regulation | No significant change in human AAA or murine models |
| Spear_2019 | miR-29a | 0.02 | . | -1 | AAA diagnosed by imaging or surgery | 6 | Non-aneurysmal aorta from organ donors | 6 | SMCs and macrophages (LCM) | Microarray | qRT-PCR | RNU6-2 | p<0.05 | Not specified | Upregulated in aneurysmal SMCs (1.6 fold), M1 (2.2 fold), and M2 (17.3 fold); downregulated in whole AAA tissue (0.5 fold, p=0.02) |
| Maegdefessel_2012 | miR-29b | 0.05 | . | -1 | AAA diagnosed by imaging or surgery | 15 | Non-aneurysmal aorta from organ donors | 5 | Infrarenal aortic tissue | None | qRT-PCR | RNU44 | p<0.05, fold change ~2.3 | Collagen and ECM regulation | Downregulated in human AAA and murine models (PPE and AngII), negatively correlated with COL1A1, COL3A1, COL5A1, ELN; regulates MMP2, MMP9 |
| Licholai_2016 | miR-29b | 0.05 | . | 1 | TAA diameter >5.5 cm by echocardiography | 15 | Non-aneurysmal aortic arch from same patient | 15 | Aortic tissue | TaqMan OpenArray | qRT-PCR (LNA PCR) | miR-193b/miR-125b | p<0.05, fold change >2 | ECM receptor interaction | Upregulated, linked to ECM remodeling |
| Spear_2019 | miR-29b | 0.05 | . | 1 | AAA diagnosed by imaging or surgery | 6 | Non-aneurysmal aorta from organ donors | 6 | SMCs (LCM) | Microarray | qRT-PCR | RNU6-2 | p<0.05 | Not specified | Upregulated in aneurysmal SMCs (1.6 fold), M1 (2.6 fold), and M2 (4.2 fold); no significant correlation with AAA diameter in plasma |
| Maegdefessel_2012 | miR-29c | 0.05 | . | -1 | AAA diagnosed by imaging or surgery | 15 | Non-aneurysmal aorta from organ donors | 5 | Infrarenal aortic tissue | None | qRT-PCR | RNU44 | p<0.05 at 7 days in PPE model | Collagen and ECM regulation | Downregulated at 7 days in PPE model, not significantly altered in human AAA or AngII model |
| Licholai_2016 | miR-29c | 0.05 | . | 1 | TAA diameter >5.5 cm by echocardiography | 15 | Non-aneurysmal aortic arch from same patient | 15 | Aortic tissue | TaqMan OpenArray | qRT-PCR (LNA PCR) | miR-193b/miR-125b | p<0.05, fold change >2 | ECM receptor interaction | Upregulated, linked to ECM remodeling |
| Spear_2019 | miR-29c | 0.05 | . | 0 | AAA diagnosed by imaging or surgery | 6 | Non-aneurysmal aorta from organ donors | 6 | M2 macrophages (LCM) | Microarray | qRT-PCR | RNU6-2 | p<0.05 | Not specified | Downregulated in aneurysmal SMCs (0.9 fold); upregulated in M1 (1.85 fold) and M2 (8.5 fold) |
| Spear_2015 | miR-30a-5p | 0.05 | . | -1 | AAA diameter >50 mm or growth >10 mm/6 months by ultrasound | 20 | Non-aneurysmal aorta from organ donors | 14 | Aortic tissue | Microarray | qRT-PCR | RNU6-2 | p<0.05 | Inflammation | Downregulated in ATLOs, linked to cytokine signaling |
| Spear_2015 | miR-30a-5p | 0.05 | . | -1 | AAA diameter >50 mm or growth >10 mm/6 months by ultrasound | 24 | PAD, no AAA by ultrasound | 18 | Plasma | Microarray | qRT-PCR | cel-miR-39 | p<0.05 | Inflammation | Potential biomarker in plasma |
| Cerna_2019 | miR-30d-5p | 0.05 | 0.05 | -1 | Large AAA (>5 cm) | 6 | Non-aneurysmal aorta from cadaveric donors | 5 | Aortic tissue | Microarray | None | Not specified | FDR<0.05, fold change >2 | Not specified | Downregulated in large AAA |
| Zhou_2022 | miR-30d-5p | 0.05 | . | -1 | AAA patients undergoing infrarenal aorta replacement | 20 | Non-aneurysmatic aortic samples and healthy donor peripheral blood | 20 | Peripheral blood and aortic tissue | Bioinformatic prediction (Encori) | qRT-PCR | dual-luciferase assay and RIP assay U6 and GAPDH | p<0.05 | NEAT1/miR-30d-5p/ADAM10 axis | Downregulated in AAA peripheral blood and H2O2-treated HVSMCs, promotes proliferation and inhibits apoptosis in HVSMCs, targets ADAM10, sponged by lncRNA NEAT1, negatively correlated with NEAT1 and ADAM10 expression |
| Cerna_2019 | miR-326 | 0.05 | 0.05 | 1 | AAA diagnosed by imaging or surgery | 16 | Non-aneurysmal aorta from cadaveric donors | 5 | Aortic tissue | Microarray | None | Not specified | FDR<0.05 | Not specified | Upregulated in small and large AAA |
| Li_2021 | miR-326 | 0.05 | . | 1 | AAA patients undergoing open surgical repair | 4 | Non-aneurysmal abdominal aortas from organ donors | 4 | Aortic tissue | Microarray (GSE144431, GSE7084 and GSE57691) | RT-qPCR | β-actin | p<0.05, \|log2FC\|>1.5 | hsa_circ_0061482/0011450/0008351/0004121/miR-326/CD8A axis | Upregulated in AAA, targets CD8A, involved in ceRNA network with upregulated circRNAs, associated with immune response and chemokine activity |
| Biros_2014 | miR-33 | 0.05 | . | 1 | AAA diameter ≥50 mm by imaging | 10 | Non-dilated AAA neck, diameter <30 mm | 10 | Aortic tissue | Luminex FlexmiR | qRT-PCR | snoRNA | >2-fold, FDR<0.5 | Lipid metabolism | Exclusive to AAA body, regulates cholesterol |
| Han_2020 | miR-33 | 0.05 | . | 1 | AAA diagnosed by CTA (>5.5 cm) | 21 | Adjacent non-aneurysmal aortic tissue | 21 | Exosomes from aortic tissue and plasma | None | qRT-PCR | U6 | p<0.05 | Not specified | Upregulated in AAA plasma and tissue exosomes |
| Venkatesh_2017 | miR-331-3p | 0.05 | . | -1 | AAA diameter >50% normal | 3 | Non-aneurysmal aorta <45 mm | 8 | Aortic tissue | None | qRT-PCR | RNU44 | p<0.05 | Not specified | Downregulated in AAA |
| Torres-Do Rego_2020 | miR-331-3p | 0.0042 | . | -1 | AAA (aortic diameter ≥30 mm) | 15 | Normal aortic diameter (<25 mm) | 32 | Plasma | qPCR array (miR CURYLNA Human panel I+II) | qRT-PCR | miR-451a | p<0.0042, fold change ≥2 | Not specified | Downregulated in SAD and AAA plasma, previously associated with aorta aneurysms |
| Venkatesh_2017 | miR-486-5p | 0.05 | . | 1 | TAA/AAA diameter >5.5 cm or >50% normal | 10 | Non-aneurysmal aorta <45 mm | 8 | Aortic tissue | Microarray | qRT-PCR | RNU44 | p<0.05 | Not specified | Upregulated in TAA and AAA |
| Courtois_2017 | miR-486-5p | 0.05 | . | -1 | AAA with positive FDG uptake (A+) | 22 | AAA without FDG uptake (A0) | 35 | Plasma | miRNA PCR array | qRT-PCR | cel-miR-39 | p<0.05, fold change >1.5 | Not specified | Downregulated in A+ plasma |
| Courtois_2017 | miR-99b-5p | 0.05 | . | -1 | AAA with positive FDG uptake (A+) | 22 | AAA without FDG uptake (A0) | 35 | Plasma | miRNA PCR array | qRT-PCR | cel-miR-39 | p<0.05, fold change >1.5 | Not specified | Downregulated in A+ plasma, also downregulated in A+pos media |
| Courtois_2017 | miR-99b-5p | 0.05 | . | -1 | AAA with positive FDG uptake (A+pos media) | 9 | AAA without FDG uptake (A0) or A+neg media | 12 | Aortic tissue | None | qRT-PCR | miR-16/SNORD95 | p<0.05 | Not specified | Downregulated in A+pos media |
| Venkatesh_2017 | miR-106b-5p | 0.05 | . | 1 | TAA diameter >5.5 cm by imaging | 5 | Non-aneurysmal aorta <45 mm | 3 | Aortic tissue | Microarray | None | RNU44 | p<0.05, fold change >1.5 | Not specified | Upregulated in TAA |
| Tao_2021 | miR-199a-5p | 0.05 | . | 1 | AAA patients undergoing surgical repair | 8 | Healthy donors | 7 | VSMCs and serum | miRNA sequencing | qRT-PCR | U6 | p<0.05, fold change ≥2 | miR-199a-5p/Sirt1 axis | Upregulated in AAA patient serum and VSMCs, targets Sirt1, promotes VSMC senescence and ROS generation, upregulated by Ang II treatment in a time-dependent manner, enhances p-p53 and p21 expression |
| Cerna_2019 | miR-27b-3p | 0.05 | 0.05 | -1 | Large AAA (>5 cm) | 6 | Non-aneurysmal aorta from cadaveric donors | 5 | Aortic tissue | Microarray | None | Not specified | FDR<0.05, fold change >2 | Not specified | Downregulated in large AAA |
| Wu_2024 | miR-28-5p | 0.001 | . | 1 | AAA patients undergoing EVAR with tumor diameter >5 cm | 120 | Non-AAA individuals with no statistical difference in comorbidities and Framingham risk score | 100 | Serum | Not specified | RT-qPCR | U6 | p<0.001 | Not specified | Upregulated in AAA serum pre-EVAR, decreased post-EVAR, positively correlated with AAA diameter (r=0.4499, p<0.0001) and diameter change (r=0.4984, p<0.0001), high post-EVAR expression predicts mortality (AUC=0.900, sensitivity=79.82%, specificity=90.91%), independent risk factor for post-EVAR death |
| Wanhainen_2017 | miR-335-5p | 0.05 | . | 1 | Fast-growing AAA (≥5 mm growth/year) | 84 | Slow-growing AAA (<5 mm growth/year) | 85 | Plasma | Microarray | qRT-PCR | Global mean | p<0.05, fold change >1.2 | Not specified | Upregulated in fast-growing AAA, AUC 0.60-0.65 |
| Biros_2014 | miR-150 | 0.45 | 0.45 | 1 | AAA diameter ≥50 mm by imaging | 10 | Non-dilated AAA neck, diameter <30 mm | 10 | Aortic tissue | Luminex FlexmiR | qRT-PCR | snoRNA | >2-fold, FDR<0.5 | Inflammation | Regulates immune cell function |
| Biros_2014 | miR-302b | 0.403 | 0.403 | 1 | AAA diameter ≥50 mm by imaging | 10 | Non-dilated AAA neck, diameter <30 mm | 10 | Aortic tissue | Luminex FlexmiR | qRT-PCR | snoRNA | >2-fold, FDR<0.5 | Not specified | Upregulated in AAA tissue |
| Biros_2014 | miR-93 | 0.45 | 0.45 | 1 | AAA diameter ≥50 mm by imaging | 10 | Non-dilated AAA neck, diameter <30 mm | 10 | Aortic tissue | Luminex FlexmiR | qRT-PCR | snoRNA | >2-fold, FDR<0.5 | Cell proliferation | Regulates cell cycle in vascular cells |
| Biros_2014 | miR-99a | 0.403 | 0.403 | 1 | AAA diameter ≥50 mm by imaging | 10 | Non-dilated AAA neck, diameter <30 mm | 10 | Aortic tissue | Luminex FlexmiR | qRT-PCR | snoRNA | >2-fold, FDR<0.5 | Not specified | Upregulated in AAA tissue |
| Biros_2014 | miR-96 | 0.05 | . | 1 | AAA diameter ≥50 mm by imaging | 10 | Non-dilated AAA neck, diameter <30 mm | 10 | Aortic tissue | Luminex FlexmiR | qRT-PCR | snoRNA | >2-fold, FDR<0.5 | Not specified | Exclusive to AAA body |
| Biros_2014 | miR-9 | 0.05 | . | 1 | AAA diameter ≥50 mm by imaging | 10 | Non-dilated AAA neck, diameter <30 mm | 10 | Aortic tissue | Luminex FlexmiR | qRT-PCR | snoRNA | >2-fold, FDR<0.5 | Inflammation | Exclusive to AAA body |
| Biros_2014 | miR-105 | 0.05 | . | 1 | AAA diameter ≥50 mm by imaging | 10 | Non-dilated AAA neck, diameter <30 mm | 10 | Aortic tissue | Luminex FlexmiR | qRT-PCR | snoRNA | >2-fold, FDR<0.5 | Not specified | Exclusive to AAA body |
| Biros_2014 | miR-220 | 0.5 | . | 0 | AAA diameter ≥50 mm by imaging | 10 | No AAA, diameter <30 mm, peripheral artery disease | 10 | Serum | Luminex FlexmiR | None | snoRNA | >2-fold, FDR<0.5 | Not specified | Non-significant in serum |
| Biros_2014 | miR-10a | 0.5 | . | 0 | AAA diameter ≥50 mm by imaging | 10 | No AAA, diameter <30 mm, peripheral artery disease | 10 | Serum | Luminex FlexmiR | None | snoRNA | >2-fold, FDR<0.5 | Inflammation | Non-significant in serum |
| Biros_2014 | miR-23b | 0.5 | . | 0 | AAA diameter ≥50 mm by imaging | 10 | No AAA, diameter <30 mm, peripheral artery disease | 10 | Serum | Luminex FlexmiR | None | snoRNA | >2-fold, FDR<0.5 | Inflammation | Non-significant in serum |
| Spear_2015 | miR-489-3p | 0.05 | . | 1 | AAA diameter >50 mm or growth >10 mm/6 months by ultrasound | 20 | Non-aneurysmal aorta from organ donors | 14 | Aortic tissue | Microarray | qRT-PCR | RNU6-2 | p<0.05 | Not specified | Upregulated in ATLOs, role unclear |
| Stather_2015 | let-7e | 0.001 | . | -1 | AAA diameter >54 mm | 80 | Healthy, no AAA by ultrasound | 40 | Whole blood | qPCR (TaqMan Cards) | Digital PCR (OpenArray) | RNU48/MammU6 | FC>1.5, p<0.1 | Inflammation | Correlated with aortic diameter |
| Stather_2015 | miR-15a | 0.001 | . | -1 | AAA diameter >54 mm | 80 | Healthy, no AAA by ultrasound | 40 | Whole blood | qPCR (TaqMan Cards) | Digital PCR (OpenArray) | RNU48/MammU6 | FC>1.5, p<0.1 | Angiogenesis | Significant in AAA and PAD |
| Stather_2015 | miR-411 | 0.001 | . | 1 | AAA diameter >54 mm | 80 | Healthy, no AAA by ultrasound | 40 | Whole blood | qPCR (TaqMan Cards) | Digital PCR (OpenArray) | RNU48/MammU6 | FC>1.5, p<0.1 | Not specified | Upregulated, not significant after regression |
| Busch_2016 | miR-550a-5p | 0.05 | . | -1 | AAA diagnosed by imaging or surgery | 19 | Atherosclerotic non-aneurysmal aorta | 11 | Aortic tissue | TaqMan Array | qRT-PCR | U6/RNU48 | p<0.05 | Not specified | Downregulated, inflammation-independent |
| Busch_2016 | miR-769-5p | 0.05 | . | -1 | AAA diagnosed by imaging or surgery | 19 | Atherosclerotic non-aneurysmal aorta | 11 | Aortic tissue | TaqMan Array | qRT-PCR | U6/RNU48 | p<0.05 | Not specified | Downregulated, inflammation-independent |
| Busch_2016 | miR-194-5p | 0.05 | . | -1 | AAA diagnosed by imaging or surgery | 19 | Atherosclerotic non-aneurysmal aorta | 11 | Aortic tissue | TaqMan Array | qRT-PCR | U6/RNU48 | p<0.05 | Angiogenesis | Downregulated, inflammation-dependent |
| Busch_2016 | miR-19b-1-5p | 0.05 | . | -1 | AAA diagnosed by imaging or surgery | 19 | Atherosclerotic non-aneurysmal aorta | 11 | Aortic tissue | TaqMan Array | qRT-PCR | U6/RNU48 | p<0.05 | Apoptosis/Angiogenesis | Downregulated, targets Bcl2/PTEN |
| Busch_2016 | miR-362-3p | 0.05 | . | -1 | AAA diagnosed by imaging or surgery | 19 | Atherosclerotic non-aneurysmal aorta | 11 | Aortic tissue | TaqMan Array | qRT-PCR | U6/RNU48 | p<0.05 | Not specified | Downregulated, inflammation-independent |
| Zhang_2015 | miR-191-3p | 0.001 | . | 1 | AAA diameter >50% normal by CT | 60 | Healthy, no AAA by ultrasound | 60 | Plasma | Microarray | qRT-PCR | cel-miR-39 | p<0.05, fold change >5 | Not specified | Upregulated, AUC 0.9700, potential biomarker |
| Zhang_2015 | miR-455-3p | 0.001 | . | 1 | AAA diameter >50% normal by CT | 60 | Healthy, no AAA by ultrasound | 60 | Plasma | Microarray | qRT-PCR | cel-miR-39 | p<0.05, fold change >5 | Not specified | Upregulated, AUC 0.9825, potential biomarker |
| Licholai_2016 | miR-143 | 0.05 | . | 1 | TAA diameter >5.5 cm by echocardiography | 15 | Non-aneurysmal aortic arch from same patient | 15 | Aortic tissue | TaqMan OpenArray | qRT-PCR (LNA PCR) | miR-193b/miR-125b | p<0.05, fold change >2 | VEGF signaling | Upregulated, linked to angiogenesis |
| Licholai_2016 | miR-19b | 0.05 | . | 1 | TAA diameter >5.5 cm by echocardiography | 15 | Non-aneurysmal aortic arch from same patient | 15 | Aortic tissue | TaqMan OpenArray | qRT-PCR (LNA PCR) | miR-193b/miR-125b | p<0.05, fold change >2 | Apoptosis | Upregulated, linked to cell death pathways |
| Licholai_2016 | miR-30c | 0.05 | . | 1 | TAA diameter >5.5 cm by echocardiography | 15 | Non-aneurysmal aortic arch from same patient | 15 | Aortic tissue | TaqMan OpenArray | qRT-PCR (LNA PCR) | miR-193b/miR-125b | p<0.05, fold change >2 | Focal adhesion | Upregulated, linked to cell adhesion |
| Chan_2016 | miR-205 | 0.001 | . | 1 | AAA diagnosed by imaging or surgery | 8 | Non-aneurysmal aorta from organ donors | 8 | Aortic tissue | None | qRT-PCR | RNU6-2 | p<0.05 | ECM remodeling | Upregulated, negatively correlated with LRP1 protein, regulates MMP-9 clearance |
| Chan_2016 | miR-338-5p | 0.61 | . | 0 | AAA diagnosed by imaging or surgery | 8 | Non-aneurysmal aorta from organ donors | 8 | Aortic tissue | None | qRT-PCR | RNU6-2 | p<0.05 | Not specified | No significant difference |
| Chan_2016 | miR-545-3p | 0.73 | . | 0 | AAA diagnosed by imaging or surgery | 8 | Non-aneurysmal aorta from organ donors | 8 | Aortic tissue | None | qRT-PCR | RNU6-2 | p<0.05 | Not specified | No significant difference |
| Gao_2017 | miR-15a-5p | 0.05 | . | 1 | AAA diagnosed by imaging or surgery | 32 | Non-aneurysmal aorta from autopsies | 29 | Aortic tissue | None | qRT-PCR | U6 | p<0.05 | Cell cycle regulation | Upregulated, negatively regulates CDKN2B, promotes SMC viability |
| Chan_2017 | miR-516a-5p | 0.05 | . | 1 | Non-aneurysmal aorta from organ donors | 6 | Scramble control VSMCs | 6 | Cultured VSMCs | None | qRT-PCR | RNU6-2 | p<0.05 | ECM remodeling | Upregulated, downregulates MTHFR/TIMP-1, upregulates MMP-2 |
| Venkatesh_2017 | miR-30c-2 | 0.05 | . | -1 | TAA/AAA diameter >5.5 cm or >50% normal | 10 | Non-aneurysmal aorta <45 mm | 8 | Aortic tissue | None | qRT-PCR | RNU44 | p<0.05 | Not specified | Downregulated in TAA and AAA |
| Venkatesh_2017 | miR-221 | 0.05 | . | 1 | TAA diameter >5.5 cm by imaging | 7 | Non-aneurysmal aorta <45 mm | 8 | Aortic tissue | None | qRT-PCR | RNU44 | p<0.05 | Not specified | Upregulated in TAA, no change in AAA |
| Venkatesh_2017 | miR-15b-5p | 0.05 | . | 1 | TAA diameter >5.5 cm by imaging | 5 | Non-aneurysmal aorta <45 mm | 3 | Aortic tissue | Microarray | None | RNU44 | p<0.05, fold change >1.5 | Not specified | Upregulated in TAA |
| Venkatesh_2017 | miR-25-3p | 0.05 | . | 1 | TAA diameter >5.5 cm by imaging | 5 | Non-aneurysmal aorta <45 mm | 3 | Aortic tissue | Microarray | None | RNU44 | p<0.05, fold change >1.5 | Not specified | Upregulated in TAA |
| Venkatesh_2017 | miR-126-3p | 0.05 | . | 1 | TAA diameter >5.5 cm by imaging | 5 | Non-aneurysmal aorta <45 mm | 3 | Aortic tissue | Microarray | None | RNU44 | p<0.05, fold change >1.5 | Angiogenesis | Upregulated in TAA |
| Venkatesh_2017 | miR-146b-5p | 0.05 | . | 1 | TAA diameter >5.5 cm by imaging | 5 | Non-aneurysmal aorta <45 mm | 3 | Aortic tissue | Microarray | None | RNU44 | p<0.05, fold change >1.5 | Inflammation | Upregulated in TAA |
| Venkatesh_2017 | miR-223-3p | 0.05 | . | 1 | TAA diameter >5.5 cm by imaging | 5 | Non-aneurysmal aorta <45 mm | 3 | Aortic tissue | Microarray | None | RNU44 | p<0.05, fold change >1.5 | Inflammation | Upregulated in TAA |
| Venkatesh_2017 | miR-451a | 0.05 | . | 1 | TAA diameter >5.5 cm by imaging | 5 | Non-aneurysmal aorta <45 mm | 3 | Aortic tissue | Microarray | None | RNU44 | p<0.05, fold change >1.5 | Not specified | Upregulated in TAA |
| Venkatesh_2017 | miR-494 | 0.05 | . | 1 | TAA diameter >5.5 cm by imaging | 5 | Non-aneurysmal aorta <45 mm | 3 | Aortic tissue | Microarray | None | RNU44 | p<0.05, fold change >1.5 | Not specified | Upregulated in TAA |
| Venkatesh_2017 | miR-642a-3p | 0.05 | . | 1 | TAA diameter >5.5 cm by imaging | 5 | Non-aneurysmal aorta <45 mm | 3 | Aortic tissue | Microarray | None | RNU44 | p<0.05, fold change >1.5 | Not specified | Upregulated in TAA |
| Venkatesh_2017 | miR-1260b | 0.05 | . | 1 | TAA diameter >5.5 cm by imaging | 5 | Non-aneurysmal aorta <45 mm | 3 | Aortic tissue | Microarray | None | RNU44 | p<0.05, fold change >1.5 | Not specified | Upregulated in TAA |
| Venkatesh_2017 | miR-1268a | 0.05 | . | 1 | TAA diameter >5.5 cm by imaging | 5 | Non-aneurysmal aorta <45 mm | 3 | Aortic tissue | Microarray | None | RNU44 | p<0.05, fold change >1.5 | Not specified | Upregulated in TAA |
| Venkatesh_2017 | miR-4284 | 0.05 | . | 1 | TAA diameter >5.5 cm by imaging | 5 | Non-aneurysmal aorta <45 mm | 3 | Aortic tissue | Microarray | None | RNU44 | p<0.05, fold change >1.5 | Not specified | Upregulated in TAA |
| Venkatesh_2017 | miR-4286 | 0.05 | . | 1 | TAA diameter >5.5 cm by imaging | 5 | Non-aneurysmal aorta <45 mm | 3 | Aortic tissue | Microarray | None | RNU44 | p<0.05, fold change >1.5 | Not specified | Upregulated in TAA |
| Venkatesh_2017 | miR-4306 | 0.05 | . | 1 | TAA diameter >5.5 cm by imaging | 5 | Non-aneurysmal aorta <45 mm | 3 | Aortic tissue | Microarray | None | RNU44 | p<0.05, fold change >1.5 | Not specified | Upregulated in TAA |
| Venkatesh_2017 | miR-4454 | 0.05 | . | 1 | TAA diameter >5.5 cm by imaging | 5 | Non-aneurysmal aorta <45 mm | 3 | Aortic tissue | Microarray | None | RNU44 | p<0.05, fold change >1.5 | Not specified | Upregulated in TAA |
| Venkatesh_2017 | miR-4459 | 0.05 | . | 1 | TAA diameter >5.5 cm by imaging | 5 | Non-aneurysmal aorta <45 mm | 3 | Aortic tissue | Microarray | None | RNU44 | p<0.05, fold change >1.5 | Not specified | Upregulated in TAA |
| Venkatesh_2017 | miR-4763-3p | 0.05 | . | 1 | TAA diameter >5.5 cm by imaging | 5 | Non-aneurysmal aorta <45 mm | 3 | Aortic tissue | Microarray | None | RNU44 | p<0.05, fold change >1.5 | Not specified | Upregulated in TAA |
| Venkatesh_2017 | miR-6090 | 0.05 | . | 1 | TAA diameter >5.5 cm by imaging | 5 | Non-aneurysmal aorta <45 mm | 3 | Aortic tissue | Microarray | None | RNU44 | p<0.05, fold change >1.5 | Not specified | Upregulated in TAA |
| Venkatesh_2017 | miR-371b-5p | 0.05 | . | -1 | TAA diameter >5.5 cm by imaging | 5 | Non-aneurysmal aorta <45 mm | 3 | Aortic tissue | Microarray | None | RNU44 | p<0.05, fold change >1.5 | Not specified | Downregulated in TAA |
| Venkatesh_2017 | miR-572 | 0.05 | . | -1 | TAA diameter >5.5 cm by imaging | 5 | Non-aneurysmal aorta <45 mm | 3 | Aortic tissue | Microarray | None | RNU44 | p<0.05, fold change >1.5 | Not specified | Downregulated in TAA |
| Venkatesh_2017 | miR-638 | 0.05 | . | -1 | TAA diameter >5.5 cm by imaging | 5 | Non-aneurysmal aorta <45 mm | 3 | Aortic tissue | Microarray | None | RNU44 | p<0.05, fold change >1.5 | Not specified | Downregulated in TAA |
| Venkatesh_2017 | miR-1227-5p | 0.05 | . | -1 | TAA diameter >5.5 cm by imaging | 5 | Non-aneurysmal aorta <45 mm | 3 | Aortic tissue | Microarray | None | RNU44 | p<0.05, fold change >1.5 | Not specified | Downregulated in TAA |
| Venkatesh_2017 | miR-1273g-3p | 0.05 | . | -1 | TAA diameter >5.5 cm by imaging | 5 | Non-aneurysmal aorta <45 mm | 3 | Aortic tissue | Microarray | None | RNU44 | p<0.05, fold change >1.5 | Not specified | Downregulated in TAA |
| Venkatesh_2017 | miR-1273f | 0.05 | . | -1 | TAA diameter >5.5 cm by imaging | 5 | Non-aneurysmal aorta <45 mm | 3 | Aortic tissue | Microarray | None | RNU44 | p<0.05, fold change >1.5 | Not specified | Downregulated in TAA |
| Venkatesh_2017 | miR-2861 | 0.05 | . | -1 | TAA diameter >5.5 cm by imaging | 5 | Non-aneurysmal aorta <45 mm | 3 | Aortic tissue | Microarray | None | RNU44 | p<0.05, fold change >1.5 | Not specified | Downregulated in TAA |
| Venkatesh_2017 | miR-3135b | 0.05 | . | -1 | TAA diameter >5.5 cm by imaging | 5 | Non-aneurysmal aorta <45 mm | 3 | Aortic tissue | Microarray | None | RNU44 | p<0.05, fold change >1.5 | Not specified | Downregulated in TAA |
| Venkatesh_2017 | miR-3652 | 0.05 | . | -1 | TAA diameter >5.5 cm by imaging | 5 | Non-aneurysmal aorta <45 mm | 3 | Aortic tissue | Microarray | None | RNU44 | p<0.05, fold change >1.5 | Not specified | Downregulated in TAA |
| Venkatesh_2017 | miR-3665 | 0.05 | . | -1 | TAA diameter >5.5 cm by imaging | 5 | Non-aneurysmal aorta <45 mm | 3 | Aortic tissue | Microarray | None | RNU44 | p<0.05, fold change >1.5 | Not specified | Downregulated in TAA |
| Venkatesh_2017 | miR-3960 | 0.05 | . | -1 | TAA diameter >5.5 cm by imaging | 5 | Non-aneurysmal aorta <45 mm | 3 | Aortic tissue | Microarray | None | RNU44 | p<0.05, fold change >1.5 | Not specified | Downregulated in TAA |
| Venkatesh_2017 | miR-4324 | 0.05 | . | -1 | TAA diameter >5.5 cm by imaging | 5 | Non-aneurysmal aorta <45 mm | 3 | Aortic tissue | Microarray | None | RNU44 | p<0.05, fold change >1.5 | Not specified | Downregulated in TAA |
| Venkatesh_2017 | miR-4507 | 0.05 | . | -1 | TAA diameter >5.5 cm by imaging | 5 | Non-aneurysmal aorta <45 mm | 3 | Aortic tissue | Microarray | None | RNU44 | p<0.05, fold change >1.5 | Not specified | Downregulated in TAA |
| Venkatesh_2017 | miR-4787-5p | 0.05 | . | -1 | TAA diameter >5.5 cm by imaging | 5 | Non-aneurysmal aorta <45 mm | 3 | Aortic tissue | Microarray | None | RNU44 | p<0.05, fold change >1.5 | Not specified | Downregulated in TAA |
| Venkatesh_2017 | miR-5001-5p | 0.05 | . | -1 | TAA diameter >5.5 cm by imaging | 5 | Non-aneurysmal aorta <45 mm | 3 | Aortic tissue | Microarray | None | RNU44 | p<0.05, fold change >1.5 | Not specified | Downregulated in TAA |
| Venkatesh_2017 | miR-6068 | 0.05 | . | -1 | TAA diameter >5.5 cm by imaging | 5 | Non-aneurysmal aorta <45 mm | 3 | Aortic tissue | Microarray | None | RNU44 | p<0.05, fold change >1.5 | Not specified | Downregulated in TAA |
| Venkatesh_2017 | miR-6089 | 0.05 | . | -1 | TAA diameter >5.5 cm by imaging | 5 | Non-aneurysmal aorta <45 mm | 3 | Aortic tissue | Microarray | None | RNU44 | p<0.05, fold change >1.5 | Not specified | Downregulated in TAA |
| Venkatesh_2017 | miR-6125 | 0.05 | . | -1 | TAA diameter >5.5 cm by imaging | 5 | Non-aneurysmal aorta <45 mm | 3 | Aortic tissue | Microarray | None | RNU44 | p<0.05, fold change >1.5 | Not specified | Downregulated in TAA |
| Venkatesh_2017 | miR-6724-5p | 0.05 | . | -1 | TAA diameter >5.5 cm by imaging | 5 | Non-aneurysmal aorta <45 mm | 3 | Aortic tissue | Microarray | None | RNU44 | p<0.05, fold change >1.5 | Not specified | Downregulated in TAA |
| Wanhainen_2017 | miR-10b-5p | 0.001 | . | 1 | AAA diameter ≥30 mm by imaging | 169 | Non-aneurysmal aorta | 48 | Plasma | Microarray | qRT-PCR | Global mean | p<0.05, AUC>0.7 | Not specified | Upregulated in AAA, AUC 0.75, specificity 70%, sensitivity 60% |
| Wanhainen_2017 | let-7i-5p | 0.05 | . | 1 | AAA diameter ≥30 mm by imaging | 169 | Non-aneurysmal aorta | 48 | Plasma | Microarray | qRT-PCR | Global mean | p<0.05 | Not specified | Upregulated in AAA, combined with miR-10b-5p, AUC 0.94 |
| Wanhainen_2017 | miR-125a-5p | 0.05 | . | 1 | Fast-growing AAA (≥5 mm growth/year) | 84 | Slow-growing AAA (<5 mm growth/year) | 85 | Plasma | Microarray | qRT-PCR | Global mean | p<0.05, fold change >1.2 | Not specified | Upregulated in fast-growing AAA, combined with miR-335-5p, AUC 0.84 |
| Cao_2017 | miR-504 | 0.01 | 0.01 | 1 | AAA diagnosed by imaging or surgery | 60 | Non-aneurysmal aortic tissue | 60 | Aortic tissue | Microarray | qRT-PCR | U6 | p<0.01, fold change >2 | p53 signaling | Upregulated, promotes SMC proliferation, inhibits apoptosis via p53 |
| Cao_2017 | let-7c | 0.05 | 0.01 | 1 | AAA diagnosed by imaging or surgery | 60 | Non-aneurysmal aortic tissue | 60 | Aortic tissue | Microarray | qRT-PCR | U6 | p<0.05, fold change >2 | Not specified | Upregulated in AAA |
| Cao_2017 | miR-449a | 0.05 | 0.01 | 1 | AAA diagnosed by imaging or surgery | 60 | Non-aneurysmal aortic tissue | 60 | Aortic tissue | Microarray | qRT-PCR | U6 | p<0.05, fold change >2 | Not specified | Upregulated in AAA |
| Cao_2017 | miR-192-5p | 0.05 | 0.01 | 1 | AAA diagnosed by imaging or surgery | 60 | Non-aneurysmal aortic tissue | 60 | Aortic tissue | Microarray | qRT-PCR | U6 | p<0.05, fold change >2 | Not specified | Upregulated in AAA |
| Cao_2017 | miR-542-3p | 0.05 | 0.01 | 1 | AAA diagnosed by imaging or surgery | 60 | Non-aneurysmal aortic tissue | 60 | Aortic tissue | Microarray | qRT-PCR | U6 | p<0.05, fold change >2 | Not specified | Upregulated in AAA |
| Courtois_2017 | miR-33a-5p | 0.05 | . | 1 | AAA with positive FDG uptake (A+) | 22 | AAA without FDG uptake (A0) | 35 | Plasma | miRNA PCR array | qRT-PCR | cel-miR-39 | p<0.05, fold change >1.5 | TGF-β signaling | Upregulated in A+ plasma |
| Meng_2018 | miR-183 | 0.05 | . | -1 | Inflammatory AAA diagnosed by CT/MRI | 36 | Adjacent non-aneurysmal tissue | 36 | Aortic tissue | None | qRT-PCR | U6 | p<0.05 | Not specified | Downregulated in IAAA, associated with smoking history and aneurysm size, prognostic factor (HR=3.587) |
| Meng_2018 | miR-141 | 0.05 | . | -1 | Inflammatory AAA diagnosed by CT/MRI | 36 | Adjacent non-aneurysmal tissue | 36 | Aortic tissue | None | qRT-PCR | U6 | p<0.05 | Not specified | Downregulated in IAAA, associated with smoking history and aneurysm size, prognostic factor (HR=3.841) |
| Shen_2018 | miR-320d | 0.0005 | . | -1 | Aortic dissection diagnosed by imaging or surgery | 10 | Non-vascular disease donors | 10 | Aortic tissue | NGS | qRT-PCR | U6 | p<0.0005, fold change >1.2 | Apoptotic pathways | Downregulated in AD, enhances apoptosis, regulates TRIAP1 |
| Shen_2018 | miR-582 | 0.05 | . | -1 | Aortic dissection diagnosed by imaging or surgery | 10 | Non-vascular disease donors | 10 | Aortic tissue | NGS | qRT-PCR | U6 | p<0.05, fold change >1.2 | Apoptotic pathways | Downregulated in AD, enhances apoptosis, regulates NET1 |
| Shen_2018 | miR-15 | 0.005 | . | -1 | Aortic dissection diagnosed by imaging or surgery | 10 | Non-vascular disease donors | 10 | Aortic tissue | NGS | qRT-PCR | U6 | p<0.005, fold change >1.2 | Not specified | Downregulated in AD, nearly undetectable |
| Tenorio_2018 | miR-455-3p | 0.0003 | . | -1 | Infrarenal AAA eligible for endovascular repair | 30 | Preoperative AAA patients (same patients, post-repair) | 30 | Whole blood | None | qRT-PCR | U6 | p<0.0003 | MMPs, IL-6, TNF-α | Downregulated post-repair, interacts with ICAM1, TIMP1, MMPs, TNF-α, DAB2IP, SERPINA1, LDLR, IL-16, MTHFR |
| Su_2019 | let-7a | 0.0003 | . | -1 | AAA diagnosed by imaging or surgery | 25 | Normal aortic tissue | 25 | Aortic tissue | None | qRT-PCR | GAPDH | p<0.0003 | IL-6 signaling | Downregulated in AAA, inversely correlated with IL-6, regulated by LINC00265 via ceRNA mechanism |
| Gan_2018 | miR-15b | 0.05 | 0.05 | 1 | AAA diagnosed by imaging or surgery | 8 | Non-aneurysmal aorta from organ donors | 2 | Aortic tissue | Microarray | None | Not specified | \|logFC\|>1, FDR<0.05 | Lipid biosynthetic process | Upregulated in AAA, regulates hub gene ACSS2 |
| Gan_2018 | miR-30a | 0.05 | 0.05 | -1 | AAA diagnosed by imaging or surgery | 8 | Non-aneurysmal aorta from organ donors | 2 | Aortic tissue | Microarray | None | Not specified | \|logFC\|>1, FDR<0.05 | Chemokine signaling pathway | Downregulated in AAA, regulates hub gene GNG2 |
| Legaki_2019 | miR-363-3p | 0.0001 | . | -1 | AAA diagnosed by imaging or surgery | 18 | Non-aneurysmal aortic neck | 15 | Aortic tissue | None | qRT-PCR | U6 | p<0.0001, fold change ~3.28 | DAB2IP regulation | Downregulated in AAA, negatively correlated with DAB2IP (r=-0.40) |
| Cerna_2019 | miR-7158-5p | 0.05 | 0.05 | -1 | AAA diagnosed by imaging or surgery | 16 | Non-aneurysmal aorta from cadaveric donors | 5 | Aortic tissue | Microarray | None | Not specified | FDR<0.05 | Not specified | Downregulated in small and large AAA |
| Cerna_2019 | miR-658 | 0.05 | 0.05 | -1 | AAA diagnosed by imaging or surgery | 16 | Non-aneurysmal aorta from cadaveric donors | 5 | Aortic tissue | Microarray | None | Not specified | FDR<0.05 | Not specified | Downregulated in small and large AAA |
| Cerna_2019 | miR-517-5p | 0.05 | 0.05 | -1 | AAA diagnosed by imaging or surgery | 16 | Non-aneurysmal aorta from cadaveric donors | 5 | Aortic tissue | Microarray | None | Not specified | FDR<0.05 | Not specified | Downregulated in small and large AAA |
| Cerna_2019 | miR-122-5p | 0.05 | 0.05 | -1 | AAA diagnosed by imaging or surgery | 16 | Non-aneurysmal aorta from cadaveric donors | 5 | Aortic tissue | Microarray | None | Not specified | FDR<0.05 | Not specified | Downregulated in small and large AAA |
| Cerna_2019 | miR-3180 | 0.05 | 0.05 | -1 | AAA diagnosed by imaging or surgery | 16 | Non-aneurysmal aorta from cadaveric donors | 5 | Aortic tissue | Microarray | None | Not specified | FDR<0.05 | Not specified | Downregulated in small and large AAA |
| Cerna_2019 | miR-23a-3p | 0.05 | 0.05 | -1 | Large AAA (>5 cm) | 6 | Non-aneurysmal aorta from cadaveric donors | 5 | Aortic tissue | Microarray | None | Not specified | FDR<0.05, fold change >2 | Not specified | Downregulated in large AAA |
| Cerna_2019 | miR-24-3p | 0.05 | 0.05 | -1 | Large AAA (>5 cm) | 6 | Non-aneurysmal aorta from cadaveric donors | 5 | Aortic tissue | Microarray | None | Not specified | FDR<0.05, fold change >2 | Not specified | Downregulated in large AAA |
| Cerna_2019 | miR-193a-3p | 0.05 | 0.05 | -1 | Large AAA (>5 cm) | 6 | Non-aneurysmal aorta from cadaveric donors | 5 | Aortic tissue | Microarray | None | Not specified | FDR<0.05, fold change >2 | Not specified | Downregulated in large AAA |
| Cerna_2019 | miR-203a-3p | 0.05 | 0.05 | -1 | Large AAA (>5 cm) | 6 | Non-aneurysmal aorta from cadaveric donors | 5 | Aortic tissue | Microarray | None | Not specified | FDR<0.05, fold change >2 | Not specified | Downregulated in large AAA |
| Cerna_2019 | miR-365a-3p | 0.05 | 0.05 | -1 | Large AAA (>5 cm) | 6 | Non-aneurysmal aorta from cadaveric donors | 5 | Aortic tissue | Microarray | None | Not specified | FDR<0.05, fold change >2 | Not specified | Downregulated in large AAA |
| Cerna_2019 | miR-4291 | 0.05 | 0.05 | -1 | Large AAA (>5 cm) | 6 | Non-aneurysmal aorta from cadaveric donors | 5 | Aortic tissue | Microarray | None | Not specified | FDR<0.05, fold change >2 | Not specified | Downregulated in large AAA |
| Cerna_2019 | miR-3663-3p | 0.05 | 0.05 | 1 | Large AAA (>5 cm) | 6 | Non-aneurysmal aorta from cadaveric donors | 5 | Aortic tissue | Microarray | None | Not specified | FDR<0.05, fold change >2 | Not specified | Upregulated in large AAA |
| Spear_2019 | let-7f | 0.03 | . | -1 | AAA diagnosed by imaging or surgery | 6 | Non-aneurysmal aorta from organ donors | 6 | SMCs (LCM) | Microarray | qRT-PCR | RNU6-2 | p<0.05 | Not specified | Downregulated in aneurysmal SMCs (0.08 fold), M1 (0.08 fold), and M2 (0.75 fold); downregulated in whole AAA tissue (0.2 fold, p=0.03) |
| Spear_2019 | miR-34a | 0.03 | . | -1 | AAA diagnosed by imaging or surgery | 6 | Non-aneurysmal aorta from organ donors | 6 | SMCs and macrophages (LCM) | Microarray | qRT-PCR | RNU6-2 | p<0.05 | Not specified | Upregulated in aneurysmal SMCs (2.7 fold), M1 (563 fold), and M2 (10 fold); downregulated in whole AAA tissue (0.4 fold, p=0.03) |
| Spear_2019 | miR-451 | 0.03 | . | 1 | AAA diagnosed by imaging or surgery | 6 | Non-aneurysmal aorta from organ donors | 6 | SMCs and macrophages (LCM) | Microarray | qRT-PCR | RNU6-2 | p<0.05 | Not specified | Upregulated in aneurysmal SMCs (17 fold, p=0.02), M1 (105 fold, p=0.02), and M2 (434 fold, p=0.03) |
| Spear_2019 | miR-199a-3p | 0.05 | . | 0 | AAA diagnosed by imaging or surgery | 6 | Non-aneurysmal aorta from organ donors | 6 | SMCs and macrophages (LCM) | Microarray | qRT-PCR | RNU6-2 | p<0.05 | Not specified | Upregulated in aneurysmal SMCs (15 fold); downregulated in M1 (0.6 fold) and M2 (0.3 fold); slightly upregulated in whole AAA tissue (1.1 fold) |
| Araujo_2019 | miR-193b-3p | 0.05 | . | -1 | AAA diagnosed by imaging or surgery | 18 | Non-aneurysmal aorta from organ donors | 6 | Aortic tissue | qPCR array | qRT-PCR | SNORD61/SNORD68/RNU6-6p | p<0.05, fold change ≥2 | Eicosanoid synthesis, metalloprotease/TIMP | Downregulated in AAA, paired interaction with ALOX5 |
| Araujo_2019 | miR-34c-5p | 0.05 | . | 0 | AAA diagnosed by imaging or surgery | 18 | Non-aneurysmal aorta from organ donors | 6 | Aortic tissue | qPCR array | qRT-PCR | SNORD61/SNORD68/RNU6-6p | p<0.05, fold change ≥2 | Not specified | No detectable expression (Ct>35) in validation cohort |
| Araujo_2019 | miR-182-5p | 0.05 | . | 0 | AAA diagnosed by imaging or surgery | 18 | Non-aneurysmal aorta from organ donors | 6 | Aortic tissue | qPCR array | qRT-PCR | SNORD61/SNORD68/RNU6-6p | p<0.05, fold change ≥2 | Not specified | Not validated due to loss of statistical relevance of paired gene TIMP1 |
| Araujo_2019 | miR-200c-3p | 0.05 | . | 0 | AAA diagnosed by imaging or surgery | 18 | Non-aneurysmal aorta from organ donors | 6 | Aortic tissue | qPCR array | qRT-PCR | SNORD61/SNORD68/RNU6-6p | p<0.05, fold change ≥2 | Not specified | Not validated due to loss of statistical relevance of paired gene FN1 |
| Araujo_2019 | miR-34b-3p | 0.05 | . | 0 | AAA diagnosed by imaging or surgery | 18 | Non-aneurysmal aorta from organ donors | 6 | Aortic tissue | qPCR array | qRT-PCR | SNORD61/SNORD68/RNU6-6p | p<0.05, fold change ≥2 | Not specified | Not validated due to loss of statistical relevance of paired gene AGTR1 |
| Han_2020 | miR-106a | 0.01 | . | 1 | AAA diagnosed by CTA (>5.5 cm) | 21 | Adjacent non-aneurysmal aortic tissue | 21 | Exosomes from aortic tissue and plasma | None | qRT-PCR | U6 | p<0.05 | TIMP-2/MMP regulation | Upregulated in AAA plasma (p<0.01) and tissue exosomes (p<0.05), promotes VSMC apoptosis, inhibits viability, targets TIMP-2, upregulates MMP-2 and MMP-12 |
| Han_2020 | miR-133 | 0.05 | . | -1 | AAA diagnosed by CTA (>5.5 cm) | 21 | Adjacent non-aneurysmal aortic tissue | 21 | Exosomes from aortic tissue and plasma | None | qRT-PCR | U6 | p<0.05 | Not specified | Downregulated in AAA tissue exosomes |
| Plana_2020 | miR-27b-3p | 0.001 | . | -1 | AAA diagnosed by CT (>5.5 cm) | 30 | Healthy volunteers (ultrasound-confirmed no AAA) | 16 | Plasma and aortic tissue | qPCR array | qRT-PCR | miR-191-5p (plasma); miR-423-5p (tissue) | p<0.05, fold change >2 or <-2 | Not specified | Downregulated in AAA tissue (2-fold, p<0.001), top variable in Random Forest for plasma |
| Plana_2020 | miR-152-3p | 0.05 | . | 1 | AAA diagnosed by CT (>5.5 cm) | 30 | Healthy volunteers (ultrasound-confirmed no AAA) | 16 | Plasma | qPCR array | qRT-PCR | miR-191-5p | p<0.05, fold change 1.49-2 | Not specified | Overexpressed in AAA plasma (fold change 1.49-2) |
| Plana_2020 | miR-103a-3p | 0.05 | . | 1 | AAA diagnosed by CT (>5.5 cm) | 30 | Healthy volunteers (ultrasound-confirmed no AAA) | 16 | Plasma and aortic tissue | qPCR array | qRT-PCR | miR-191-5p (plasma); miR-423-5p (tissue) | p<0.05, fold change >2 or <-2 | Not specified | Upregulated in AAA tissue (1.3-fold, p=0.05) and plasma (fold change 1.49-2) |
| Plana_2020 | miR-221-3p | 0.05 | . | 1 | AAA diagnosed by CT (>5.5 cm) | 30 | Healthy volunteers (ultrasound-confirmed no AAA) | 16 | Plasma and aortic tissue | qPCR array | qRT-PCR | miR-191-5p (plasma); miR-423-5p (tissue) | p<0.05, fold change 1.49-2 | Not specified | Overexpressed in AAA plasma (fold change 1.49-2) |
| Plana_2020 | miR-99a-5p | 0.05 | . | 1 | AAA diagnosed by CT (>5.5 cm) | 30 | Healthy volunteers (ultrasound-confirmed no AAA) | 16 | Plasma | qPCR array | qRT-PCR | miR-191-5p | p<0.05, fold change 1.49-2 | Not specified | Overexpressed in AAA plasma (fold change 1.49-2) |
| Plana_2020 | miR-29b-3p | 0.018 | . | -1 | AAA diagnosed by CT (>5.5 cm) | 21 | Organ donors (no AAA) | 8 | Aortic tissue | None | qRT-PCR | miR-423-5p | p<0.05, fold change >2 or <-2 | Not specified | Downregulated in AAA tissue (1.4-fold, p=0.018) |
| Plana_2020 | miR-155-5p | 0.05 | . | 0 | AAA diagnosed by CT (>5.5 cm) | 21 | Organ donors (no AAA) | 8 | Aortic tissue | None | qRT-PCR | miR-423-5p | p>0.05 | Not specified | No significant change in AAA tissue |
| Plana_2020 | miR-195-5p | 0.023 | . | -1 | AAA diagnosed by CT (>5.5 cm) | 21 | Organ donors (no AAA) | 8 | Aortic tissue | qPCR array | qRT-PCR | miR-423-5p | p<0.05, fold change >2 or <-2 | THBS2 regulation | Downregulated in AAA tissue (1.6-fold, p=0.023), negatively correlated with THBS2 (ρ=-0.665, p=0.0026) |
| Plana_2020 | miR-7-1-3p | 0.05 | . | 0 | AAA diagnosed by CT (>5.5 cm) | 30 | Healthy volunteers (ultrasound-confirmed no AAA) | 16 | Plasma | qPCR array | qRT-PCR | miR-191-5p | p>0.05 | Not specified | Undetectable in plasma (CT>35), poor expression in tissue |
| Li_2020 | miR-4685-5p | 0.05 | . | -1 | AAA in VSMCs and AngII-induced mouse model | 9 | Sham-operated saline-treated mice | 9 | VSMCs and aortic tissue | RegRNA 2.0/DIANA | qRT-PCR | U6 | p<0.05 | LBX2-AS1/LBX2 ceRNA network | Downregulated in AAA, sponged by LBX2-AS1, targets LBX2, promotes VSMC proliferation, inhibits apoptosis |
| Torres-Do Rego_2020 | let-7e-5p | 0.0042 | . | -1 | AAA (aortic diameter ≥30 mm) | 15 | Normal aortic diameter (<25 mm) | 32 | Plasma | qPCR array (miR CURYLNA Human panel I+II) | qRT-PCR | miR-451a | p<0.0042, fold change ≥2 | Not specified | Downregulated in SAD and AAA plasma, related to AAA molecular mechanisms |
| Torres-Do Rego_2020 | miR-28-3p | 0.0042 | . | -1 | AAA (aortic diameter ≥30 mm) | 15 | Normal aortic diameter (<25 mm) | 32 | Plasma | qPCR array (miR CURYLNA Human panel I+II) | qRT-PCR | miR-451a | p<0.0042, fold change ≥2 | Not specified | Downregulated in SAD and AAA plasma, high fold decrease |
| Torres-Do Rego_2020 | miR-29a-3p | 0.0042 | . | -1 | AAA (aortic diameter ≥30 mm) | 15 | Normal aortic diameter (<25 mm) | 32 | Plasma | qPCR array (miR CURYLNA Human panel I+II) | qRT-PCR | miR-451a | p<0.0042, fold change ≥2 | Not specified | Downregulated in SAD and AAA plasma, negatively correlated with age (r=-0.341, p=0.008), previously associated with aorta aneurysms |
| Torres-Do Rego_2020 | miR-93-3p | 0.0042 | . | -1 | AAA (aortic diameter ≥30 mm) | 15 | Normal aortic diameter (<25 mm) | 32 | Plasma | qPCR array (miR CURYLNA Human panel I+II) | qRT-PCR | miR-451a | p<0.0042, fold change ≥2 | Not specified | Downregulated in SAD and AAA plasma, negatively correlated with systolic blood pressure (r=-0.272, p=0.039), previously associated with aorta aneurysms |
| Torres-Do Rego_2020 | miR-338-3p | 0.0042 | . | -1 | AAA (aortic diameter ≥30 mm) | 15 | Normal aortic diameter (<25 mm) | 32 | Plasma | qPCR array (miR CURYLNA Human panel I+II) | qRT-PCR | miR-451a | p<0.0042, fold change ≥2 | Not specified | Downregulated in SAD and AAA plasma, high fold decrease |
| Torres-Do Rego_2020 | miR-339-3p | 0.0042 | . | -1 | AAA (aortic diameter ≥30 mm) | 15 | Normal aortic diameter (<25 mm) | 32 | Plasma | qPCR array (miR CURYLNA Human panel I+II) | qRT-PCR | miR-451a | p<0.0042, fold change ≥2 | ATP5, KLHL15, WDTC1, PIPNM3, MEX3D, NFKB1, MCL1, FOXO1, PHLDA2, IGF2, RPL10A, SHOX2, AKIRIN1, HEYL | Downregulated in SAD and AAA plasma, highest AUC for SAD diagnosis (sensitivity 80.77%, specificity 77.27%), OR=8.78 (p=0.006), high fold decrease |
| Torres-Do Rego_2020 | miR-378a-3p | 0.0042 | . | -1 | AAA (aortic diameter ≥30 mm) | 15 | Normal aortic diameter (<25 mm) | 32 | Plasma | qPCR array (miR CURYLNA Human panel I+II) | qRT-PCR | miR-451a | p<0.0042, fold change ≥2 | Not specified | Downregulated in SAD and AAA plasma, related to AAA molecular mechanisms |
| Zalewski_2020 | hsa-miR-34a-5p | 0.0001 | Benjamini-Hochberg | 1 | Intrarenal true AAA (duplex ultrasonography and CT) | 28 | Healthy, non-smoking, no AAA (duplex ultrasound) | 19 | PBMCs | Small RNA sequencing (Ion S5 XL) | DESeq2 and UVE-PLS | None | p<0.0001, adjusted by Benjamini-Hochberg | Cardiovascular diseases, aging | Upregulated, positively correlated with maximum aneurysm diameter (R=0.42, p=0.025), AUC=0.981-0.795 |
| Zalewski_2020 | hsa-miR-574-5p | 0.0001 | Benjamini-Hochberg | 1 | Intrarenal true AAA (duplex ultrasonography and CT) | 28 | Healthy, non-smoking, no AAA (duplex ultrasound) | 19 | PBMCs | Small RNA sequencing (Ion S5 XL) | DESeq2 and UVE-PLS | None | p<0.0001, adjusted by Benjamini-Hochberg | Not specified | Upregulated, positively correlated with thrombus volume, AUC=0.981-0.795 |
| Zalewski_2020 | hsa-miR-769-5p | 0.0001 | Benjamini-Hochberg | 1 | Intrarenal true AAA (duplex ultrasonography and CT) | 28 | Healthy, non-smoking, no AAA (duplex ultrasound) | 19 | PBMCs | Small RNA sequencing (Ion S5 XL) | DESeq2 and UVE-PLS | None | p<0.0001, adjusted by Benjamini-Hochberg | Not specified | Upregulated, associated with age, AUC=0.981-0.795 |
| Zalewski_2020 | hsa-miR-7847-3p | 0.0001 | Benjamini-Hochberg | 1 | Intrarenal true AAA (duplex ultrasonography and CT) | 28 | Healthy, non-smoking, no AAA (duplex ultrasound) | 19 | PBMCs | Small RNA sequencing (Ion S5 XL) | DESeq2 and UVE-PLS | None | p<0.0001, adjusted by Benjamini-Hochberg | Not specified | Upregulated, associated with age, AUC=0.981-0.795 |
| Zhao_2020 | miR-7 | 0.05 | . | 1 | Ascending aortic aneurysm (surgical patients) | 10 | Non-aneurysmal aortas from coronary artery bypass graft patients | 10 | Aortic tissue and primary VSMCs | RT-qPCR | RT-qPCR | U6 | p<0.05 | CDR1as/miR-7/CKAP4 axis | Upregulated in AAA tissues, promotes VSMC apoptosis, inhibits proliferation, sponged by CDR1as, targets CKAP4 |
| Tian_2020 | miR-212-5p | 0.05 | . | -1 | AAA (surgical patients) | 20 | Normal abdominal aortic tissues from trauma patients | 16 | Aortic tissue and VSMCs | RT-qPCR | RT-qPCR | GAPDH and U6 | p<0.05 | LINC00473/miR-212-5p/BASP1 axis | Downregulated in AAA tissues and H2O2-treated VSMCs, inhibits VSMC apoptosis, promotes proliferation, sponged by LINC00473, targets BASP1, negatively correlated with LINC00473 and BASP1 expression in AAA tissues |
| Zhao_2020 | miR-33-5p | 0.01 | . | 1 | AAA (pathologically confirmed) | 20 | Non-AAA tissues | 20 | AAA tissue | qRT-PCR | RT-qPCR and Western blot | U6 | p<0.05 | miR-33-5p/ABCA1/PI3K/Akt axis | Upregulated in AAA tissues (p<0.01), negatively correlated with ABCA1 mRNA (r=-0.6922, p=0.0007), targets ABCA1, inhibits PI3K/Akt pathway, promotes foam cell formation, increases MMP-2, MMP-9, and TNF-α expression, inhibits cholesterol efflux |
| Zou_2021 | miR-1264 | 0.05 | . | -1 | AAA (not treated) | 24 | Healthy volunteers | 24 | Serum and HA-VSMCs | qRT-PCR | RT-qPCR | U6 | p<0.05 | XIST/miR-1264/WNT5A axis | Downregulated in AAA patient serum and AAA mouse model tissues, promotes HA-VSMC proliferation, inhibits apoptosis, sponged by XIST, targets WNT5A, negatively correlated with XIST and WNT5A expression, regulates WNT/β-catenin signaling |
| Liu_2021 | miR-143-3p | 0.05 | . | -1 | AAA patients undergoing open surgery | 25 | Patients undergoing coronary artery bypass graft | 21 | Aortic tissue and VSMCs | qRT-PCR | qRT-PCR and RIP assay | U6 and β-actin | p<0.05 | circ-FNDC3B/miR-143-3p/ADAM10 axis | Downregulated in AAA tissues and VSMCs, promotes VSMC viability, proliferation, and reduces apoptosis, inflammation, and oxidative stress, sponged by circ-FNDC3B, targets ADAM10, negatively correlated with circ-FNDC3B and ADAM10 expression |
| Li_2021 | miR-330-5p | 0.05 | . | -1 | AAA patients undergoing open surgical repair | 4 | Non-aneurysmal abdominal aortas from organ donors | 4 | Aortic tissue | Microarray (GSE144431,GSE7084 and GSE57691) | RT-qPCR | β-actin | p<0.05, \|log2FC\|>1.5 | hsa_circ_0057691/0092108/0006845/0082182/miR-330-5p/CNN1 axis | Downregulated in AAA, targets CNN1, involved in ceRNA network with downregulated circRNAs, associated with vascular smooth muscle contraction and actin binding |
| Si_2021 | miR-635 | 0.01 | . | Not specified | AAA tissue samples | 97 | Normal artery tissue from brain-dead patients | 23 | Aortic tissue | Microarray (GSE47472, GSE52093, GSE57691, GSE98278 , GSE144431) | TargetScan and Circinteractome databases | Not specified | p<0.01, \|log2FC\|>2 | circRNA-miRNA-mRNA network | Identified as common miRNA interacting with hub genes and circRNAs, involved in AAA-related ceRNA network |
| Si_2021 | miR-527 | 0.01 | . | Not specified | AAA tissue samples | 97 | Normal artery tissue from brain-dead patients | 23 | Aortic tissue | Microarray (GSE47472, GSE52093, GSE57691, GSE98278 , GSE144431) | TargetScan and Circinteractome databases | Not specified | p<0.01, \|log2FC\|>2 | circRNA-miRNA-mRNA network | Identified as common miRNA interacting with hub genes and circRNAs, involved in AAA-related ceRNA network |
| Si_2021 | miR-520h | 0.01 | . | Not specified | AAA tissue samples | 97 | Normal artery tissue from brain-dead patients | 23 | Aortic tissue | Microarray (GSE47472, GSE52093, GSE57691, GSE98278 , GSE144431) | TargetScan and Circinteractome databases | Not specified | p<0.01, \|log2FC\|>2 | circRNA-miRNA-mRNA network | Identified as common miRNA interacting with hub genes and circRNAs, involved in AAA-related ceRNA network |
| Si_2021 | miR-938 | 0.01 | . | Not specified | AAA tissue samples | 97 | Normal artery tissue from brain-dead patients | 23 | Aortic tissue | Microarray (GSE47472, GSE52093, GSE57691, GSE98278 , GSE144431) | TargetScan and Circinteractome databases | Not specified | p<0.01, \|log2FC\|>2 | circRNA-miRNA-mRNA network | Identified as common miRNA interacting with hub genes and circRNAs, involved in AAA-related ceRNA network |
| Si_2021 | miR-518a-5p | 0.01 | . | Not specified | AAA tissue samples | 97 | Normal artery tissue from brain-dead patients | 23 | Aortic tissue | Microarray (GSE47472, GSE52093, GSE57691, GSE98278 , GSE144431) | TargetScan and Circinteractome databases | Not specified | p<0.01, \|log2FC\|>2 | circRNA-miRNA-mRNA network | Identified as common miRNA interacting with hub genes and circRNAs, involved in AAA-related ceRNA network |
| Si_2021 | miR-1206 | 0.01 | . | Not specified | AAA tissue samples | 97 | Normal artery tissue from brain-dead patients | 23 | Aortic tissue | Microarray (GSE47472, GSE52093, GSE57691, GSE98278 , GSE144431) | TargetScan and Circinteractome databases | Not specified | p<0.01, \|log2FC\|>2 | circRNA-miRNA-mRNA network | Identified as common miRNA interacting with hub genes and circRNAs, involved in AAA-related ceRNA network |
| Nie_2021 | miR-205-5p | 0.05 | 0.05 | 1 | AAA patients undergoing surgical resection | 8 | Normal aortic tissues | 10 | Aortic tissue and VSMCs | Microarray (GSE57691) | qRT-PCR, luciferase assay and RIP assay | U6 | p<0.05, \|FC\|≥1 | SNHG5/miR-205-5p/SMAD4 axis | Upregulated in AAA tissues, promotes VSMC apoptosis, inhibits proliferation and migration, sponged by lncRNA SNHG5, targets SMAD4, involved in ceRNA network |
| Ma_2022 | miR-626 | 0.05 | . | 1 | AAA patients | 32 | Normal volunteers | 17 | Serum and VSMCs | Bioinformatic prediction (GSE144431 Circinteractome TargetScan) | RT-qPCR | dual-luciferase assay RIP assay and RNA pull-down assay U6 and β-actin | p<0.05 | circ_0092291/miR-626/COL4A1 axis | Upregulated in AAA serum and Ang II-treated VSMCs, promotes inflammation and apoptosis, inhibits angiopoiesis, targets COL4A1, sponged by circ_0092291, negatively correlated with circ_0092291 and COL4A1 expression |
| Ma_2022 | miR-149-5p | 0.05 | 0.1 | -1 | AAA patients | 15 | Non-AAA patients undergoing aorta-bifemoral bypass surgery | 19 | Aortic tissue | Bioinformatic prediction (GSE144431 Circbank Encori TargetScan) | qRT-PCR | dual-luciferase assay RNA pull-down assay and FISH U6 and GAPDH | p<0.05, FC>1.5, FDR<0.1 | hsa_circ_0087352/miR-149-5p/IL-6 axis | Downregulated in LPS-stimulated THP-1 macrophages, targets IL-6 and TNF-α, sponged by hsa_circ_0087352, involved in ERK/NF-κB-mediated inflammatory response in macrophages, promotes VSMC apoptosis |
| Li_2023 | miR-143-5p | 0.05 | . | -1 | AAA patients undergoing surgical treatment | 18 | Adjacent normal aortic tissues | 18 | Aortic tissue and murine macrophages | Bioinformatic prediction (JASPAR, miRanda) | RT-qPCR | dual-luciferase assay ChIP assay, Western blot and ELISA , U6 and β-actin | p<0.05 | TCF3/miR-143-5p/CCL20 axis | Downregulated in AAA tissues and Ang II-induced macrophages, promotes M2 macrophage polarization, inhibits MMP secretion and inflammatory response, targets CCL20, transcriptionally activated by TCF3, reduces AAA progression |
| Ren_2023 | miR-599 | 0.05 | . | -1 | AAA patients | 24 | Normal abdominal aortic samples from coronary artery bypass graft patients | 24 | Aortic tissue and VSMCs | Bioinformatic prediction | RT-qPCR | dual-luciferase assay and Western blot U6 and GAPDH | p<0.05 | circ_0000285/miR-599/RGS17 axis | Downregulated in AAA tissues and H2O2-treated VSMCs, promotes proliferation and inhibits apoptosis in VSMCs, targets RGS17, sponged by circ_0000285, negatively correlated with circ_0000285 and RGS17 expression |
| Thanigaimani_2032 | miR-548n | 0.05 | 0.05 | -1 | AAA patients with infrarenal aortic diameter ≥30 mm | 108 | Healthy controls and PAD patients | 24 | Serum | NanoString Human miRNA v3 assay | Not validated | hsa-miR-24-3p and hsa-miR-484 | p<0.05, q<0.05 | Regulation of cellular components, metabolic processes, mRNA stability, and protein localization | Downregulated in AAA serum, associated with AAA diagnosis (OR 0.21 vs. healthy controls, OR 0.23 vs. PAD), improves diagnostic AUC with let-7b-5p to 98.0% |
| Tian_2023 | miR-191-5p | 0.05 | . | 1 | AAA patients | 43 | Normal aortic tissues | 43 | Aortic tissue and VSMCs | Bioinformatic prediction (starBase microT, miRanda, miRmap, PicTar, TargetScan) | qRT-PCR | dual-luciferase assay RNA pull-down, Western blot, ELISA, U6 and GAPDH | p<0.05 | MIR503HG/miR-191-5p/PLCD1 axis | Upregulated in AAA tissues and VSMCs, promotes apoptosis, ECM degradation, and inflammation, targets PLCD1, sponged by lncRNA MIR503HG, negatively correlated with MIR503HG and PLCD1 expression |
| Li_2023 | miR-125b | 0.05 | . | -1 | AAA patients undergoing EVAR | 4 | Healthy controls and AAA patients post-EVAR | 3 | Peripheral blood mononuclear cells (PBMCs) | RNA sequencing (Illumina Hiseq2000/2500) | Not validated | Not specified | p<0.05 | Regulation of transcription, nervous system development, cell cycle, PI3K-Akt signaling pathway | Downregulated in AAA PBMCs compared to healthy controls (40/77 miRNAs) and post-EVAR (19/66 miRNAs), part of ceRNA network with lncRNAs and mRNAs |
| Li_2023 | miR-181 | 0.05 | . | -1 | AAA patients undergoing EVAR | 4 | Healthy controls and AAA patients post-EVAR | 3 | Peripheral blood mononuclear cells (PBMCs) | RNA sequencing (Illumina Hiseq2000/2500) | Not validated | Not specified | p<0.05 | Regulation of transcription, nervous system development, cell cycle, PI3K-Akt signaling pathway | Downregulated in AAA PBMCs compared to healthy controls (40/77 miRNAs) and post-EVAR (19/66 miRNAs), part of ceRNA network with lncRNAs and mRNAs |
| Li_2023 | miR-296 | 0.05 | . | -1 | AAA patients undergoing EVAR | 4 | Healthy controls and AAA patients post-EVAR | 3 | Peripheral blood mononuclear cells (PBMCs) | RNA sequencing (Illumina Hiseq2000/2500) | Not validated | Not specified | p<0.05 | Regulation of transcription, nervous system development, cell cycle, PI3K-Akt signaling pathway | Downregulated in AAA PBMCs compared to healthy controls (40/77 miRNAs) and post-EVAR (19/66 miRNAs), part of ceRNA network with lncRNAs and mRNAs |
| Li_2023 | miR-542 | 0.05 | . | -1 | AAA patients undergoing EVAR | 4 | Healthy controls and AAA patients post-EVAR | 3 | Peripheral blood mononuclear cells (PBMCs) | RNA sequencing (Illumina Hiseq2000/2500) | Not validated | Not specified | p<0.05 | Wnt signaling pathway, RNA transport, neurotrophin signaling pathway | Downregulated in AAA PBMCs, part of lncRNA-miRNA-mRNA ceRNA network with SATB1-AS1, TTN-AS1, and mRNAs like REL, ZNB, MAK16 |
| Li_2023 | miR-3130 | 0.05 | . | -1 | AAA patients undergoing EVAR | 4 | Healthy controls and AAA patients post-EVAR | 3 | Peripheral blood mononuclear cells (PBMCs) | RNA sequencing (Illumina Hiseq2000/2500) | Not validated | Not specified | p<0.05 | Wnt signaling pathway, RNA transport, neurotrophin signaling pathway | Downregulated in AAA PBMCs, part of lncRNA-miRNA-mRNA ceRNA network with SATB1-AS1, TTN-AS1, and mRNAs like REL, ZNB, MAK16 |
| Li_2023 | miR-320 | 0.05 | . | -1 | AAA patients undergoing EVAR | 4 | Healthy controls and AAA patients post-EVAR | 3 | Peripheral blood mononuclear cells (PBMCs) | RNA sequencing (Illumina Hiseq2000/2500) | Not validated | Not specified | p<0.05 | Wnt signaling pathway, RNA transport, neurotrophin signaling pathway, T cell receptor signaling pathway, osteoclast differentiation, C-type lectin receptor signaling pathway | Downregulated in AAA PBMCs, part of both lncRNA-miRNA-mRNA and circRNA-miRNA-mRNA ceRNA networks with circRNAs like hsa-circ-0129245, hsa-circ-0138746, and mRNAs like MAK16, NBPF11, REL |
| Wang_2024 | miRNA-325 | 0.05 | . | -1 | Ang II-induced AAA in ApoE-KO mice | 10 | Control ApoE-KO mice without Ang II treatment | 10 | Aortic tissue | Not specified | RT-PCR | Immunoblotting | p<0.05 | miRNA-325/SNAI1/MMP-2/MMP-9 axis | Downregulated in Ang II-treated aortic tissues, miRNA-325 mimics reduce AAA severity (maximal aortic diameter: 0.95 mm vs. 2.2 mm in Ang II group), decrease AAA incidence (60% vs. 90%), suppress SNAI1, MMP-2, and MMP-9 expression, and mitigate elastin degradation |
| Leite_2025 | miR-181-b | 0.05 | . | 1 | Patients with infrarenal AAA eligible for endovascular repair | 47 | Volunteers without AAA or risk factors | 10 | Whole blood | Not specified | RQ-PCR (TaqMan Assay-on-demand) | U6 | p<0.05 | Not specified | Elevated 3.86-fold in AAA patients preoperatively compared to controls, reduced expression 6 months post-endovascular repair, no significant change in expression in patients with endoleaks |

| Supplementary table 3. Quality assessment using NOS tool. | | | | | | | | | |
| --- | --- | --- | --- | --- | --- | --- | --- | --- | --- |
| Study | Selection Case Definition | Selection Representativeness | Selection Controls | Definition Controls | Comparability Confounders | Exposure Ascertainment | Exposure Same Method | Exposure non-response | Total Score |
| Maegdefessel_2012 | 1 | 0 | 1 | 1 | 2 | 1 | 1 | 0 | 7 |
| Maegdefessel_2013 | 1 | 0 | 1 | 1 | 2 | 1 | 1 | 0 | 7 |
| Biros_2014 | 1 | 0 | 1 | 1 | 2 | 1 | 1 | 0 | 7 |
| Maegdefessel_2014 | 1 | 0 | 1 | 1 | 2 | 1 | 1 | 0 | 7 |
| Spear_2015 | 1 | 0 | 1 | 1 | 2 | 1 | 1 | 0 | 7 |
| Stather_2015 | 1 | 0 | 1 | 1 | 1 | 1 | 1 | 0 | 6 |
| Busch_2016 | 1 | 0 | 1 | 1 | 2 | 1 | 1 | 0 | 7 |
| Zhang_2015 | 1 | 0 | 1 | 1 | 2 | 1 | 1 | 0 | 7 |
| Wanhainen_2016 | 1 | 0 | 1 | 1 | 2 | 1 | 1 | 0 | 7 |
| Ni_2016 | 1 | 1 | 1 | 1 | 1 | 1 | 1 | 0 | 7 |
| Venkatesh_2017 | 1 | 0 | 1 | 1 | 1 | 1 | 1 | 0 | 6 |
| Gao_2017 | 1 | 1 | 1 | 1 | 0 | 1 | 1 | 1 | 7 |
| Liang_2017 | 1 | 0 | 1 | 1 | 1 | 1 | 1 | 0 | 6 |
| Courtois_2017 | 1 | 0 | 1 | 1 | 1 | 1 | 1 | 0 | 6 |
| Ma_2018 | 1 | 0 | 1 | 1 | 0 | 1 | 1 | 0 | 5 |
| Riches_2018 | 1 | 0 | 1 | 1 | 2 | 1 | 1 | 0 | 7 |
| Tenorio_2018 | 1 | 0 | 1 | 1 | 2 | 1 | 1 | 0 | 7 |
| Cerna_2019 | 1 | 0 | 1 | 1 | 1 | 1 | 1 | 0 | 6 |
| Spear_2019 | 1 | 0 | 1 | 1 | 1 | 1 | 1 | 0 | 6 |
| Araujo_2019 | 1 | 0 | 1 | 1 | 2 | 1 | 1 | 0 | 7 |
| Zhao_2020 | 1 | 0 | 1 | 1 | 1 | 1 | 1 | 0 | 6 |
| Han_2020 | 1 | 0 | 1 | 1 | 0 | 1 | 1 | 0 | 5 |
| Plana_2020 | 1 | 0 | 1 | 1 | 0 | 1 | 1 | 0 | 5 |
| Torres-Do Rego_2020 | 1 | 0 | 1 | 1 | 2 | 1 | 1 | 0 | 7 |
| Zhang_2020 | 1 | 0 | 1 | 1 | 2 | 1 | 1 | 0 | 7 |
| Missae_2020 | 1 | 0 | 1 | 1 | 2 | 1 | 1 | 0 | 7 |
| Zalewski_2020 | 1 | 0 | 1 | 1 | 2 | 1 | 1 | 0 | 7 |
| Lichoإ‚ai_2021 | 1 | 0 | 1 | 1 | 2 | 1 | 1 | 1 | 8 |
| Zhou_2022 | 1 | 0 | 1 | 1 | 2 | 1 | 1 | 1 | 8 |
| Wang_2022 | 1 | 0 | 1 | 1 | 2 | 1 | 1 | 1 | 8 |
| Jing_2023 | 1 | 0 | 1 | 1 | 2 | 1 | 1 | 1 | 8 |
| Thanigaimani_2032 | 1 | 0 | 1 | 1 | 2 | 1 | 1 | 1 | 8 |
| Li_2023 | 1 | 0 | 1 | 1 | 0 | 1 | 1 | 0 | 5 |
| Tian_2023 | 1 | 0 | 1 | 1 | 2 | 1 | 1 | 0 | 7 |
| Cai_2024 | 1 | 0 | 1 | 1 | 2 | 1 | 1 | 0 | 7 |
| Tasopoulou_2024 | 1 | 0 | 1 | 1 | 2 | 1 | 1 | 0 | 7 |
| Xiao_2024 | 1 | 0 | 1 | 1 | 2 | 1 | 1 | 1 | 8 |
| Leite_2025 | 1 | 0 | 1 | 1 | 2 | 1 | 1 | 1 | 8 |
| Winski_2025 | 1 | 0 | 1 | 1 | 2 | 1 | 1 | 0 | 7 |
